# Development of a Thermal Infrared Network for Volcanic and Environmental Monitoring: Hardware Design and Data Analysis Software Code

**DOI:** 10.3390/s25134141

**Published:** 2025-07-02

**Authors:** Fabio Sansivero, Giuseppe Vilardo, Ciro Buonocunto

**Affiliations:** Istituto Nazionale di Geofisica e Vulcanologia-Sezione di Napoli Osservatorio Vesuviano, 80124 Napoli, Italy; giuseppe.vilardo@ingv.it (G.V.); ciro.buonocunto@ingv.it (C.B.)

**Keywords:** thermal infrared, image processing, temperature time series, radiative heat flux, monitoring network, scientific software development, temperature data processing

## Abstract

Thermal infrared (TIR) ground observations are a well-established method for investigating surface temperature variations in thermally anomalous areas. However, commercially available technical solutions are currently limited, often offering proprietary products with minimal customization options for establishing a permanent TIR monitoring network. This work presents the comprehensive development of a thermal infrared monitoring network, detailing everything from the hardware schematics of the remote monitoring station (RMS) to the code for the final data processing software. The procedures implemented in the RMS for managing TIR sensor operations, acquiring environmental data, and transmitting data remotely are thoroughly discussed, along with the technical solutions adopted. The processing of TIR imagery is carried out using ASIRA (Automated System of InfraRed Analysis), a free software package, now developed for GNU Octave. ASIRA performs quality filtering and co-registration, and applies various seasonal correction methodologies to extract time series of deseasoned surface temperatures, estimate heat fluxes, and track variations in thermally anomalous areas. Processed outputs include binary, Excel, and CSV formats, with interactive HTML plots for visualization. The system’s effectiveness has been validated in active volcanic areas of southern Italy, demonstrating high reliability in detecting anomalous thermal behavior and distinguishing endogenous geophysical processes. The aim of this work is to enable readers to easily replicate and deploy this open-source, low-cost system for the continuous, automated thermal monitoring of active volcanic and geothermal areas and environmental pollution, thereby supporting hazard assessment and scientific research.

## 1. Introduction

Monitoring surface temperatures in diffuse degassing areas is crucial for understanding the dynamics of shallow magmatic systems and their interaction with hydrothermal systems in both open-vent and closed-conduit volcanoes [1,2,3,4,5,6,7,8,9]. Variations in surface temperatures reflect changes in the efficiency of magma or magmatic fluids transfer from the shallow magmatic system to the surface and can provide critical insights into the volcano’s state. Several studies have identified thermal precursor signals associated with significant changes in activity at open-vent volcanoes [10,11,12,13,14,15,16,17,18,19,20] and have also reported notable thermal variations in closed-conduit volcanic systems [21,22,23,24,25]. Based on these findings, the development of a permanent thermal infrared (TIR) imaging network for monitoring diffuse degassing areas would represent a significant advancement in detecting changes in volcanic behavior through multi-parameter surveillance [26,27,28,29,30].

Thermal infrared ground observations are also widely applied in monitoring slope instability [31,32,33,34,35], detecting environmental pollution [36,37,38,39], and investigating other thermally driven phenomena.

However, the continuous and reliable acquisition of high-quality TIR data in remote or hazardous environments remains technically challenging due to limitations related to logistics, power supply, and data transmission constraints. Furthermore, comprehensive software solutions for automated TIR image processing are currently available only using commercial software [28]. To address these limitations, this work presents the complete development of a thermal infrared (TIR) monitoring network. The system is centered around a Raspberry Pi-based remote monitoring station (RMS) and integrates modular and flexible hardware with custom-developed open-source operational software, enabling long-term, real-time, or scheduled acquisition in harsh and remote environments. Data processing is handled by the open-source ASIRA software package, recently restructured and ported from MATLAB to the GNU Octave platform. This transition from proprietary to open-source tools promotes transparency, reproducibility, and broader accessibility for the scientific community.

This paper details the system architecture, software implementation, and performance validation, with a focus on applications in volcanic and geothermal monitoring. It covers all aspects of system development, from the design of the RMS—which captures TIR images and uploads them to a central server—to the data analysis software (ASIRA), which extracts temperatures and heat flux time series from raw TIR data. A comprehensive description of both the hardware components and the software control modules (ICARO) of the RMS is provided, along with a configuration guide for full system deployment. Although this work focuses on the FLIR SC655 thermal infrared camera, the RMS is designed to be compatible with other TIR image sensors by modifying the connection strings within the software modules.

The functionalities of the ASIRA software are also described in a detailed user manual. ASIRA processes raw TIR imagery to create deseasoned temperature time series and estimates of radiative heat flux for the areas under investigation. 

Finally, as a practical example of the acquisition and processing workflow, we present plots obtained by applying ASIRA to a twenty-year dataset of thermal infrared images acquired at the Solfatara volcanic vent (Pozzuoli, Italy).

The objective of this work is to provide a scalable, replicable, and open-source solution to support multidisciplinary efforts in TIR monitoring within geophysical and environmental research fields.

## 2. Materials and Methods

### 2.1. Overview of the Thermal Infrared Network

The primary objective of the thermal infrared network is to investigate temperature changes, radiative heat flux, and their spatial distributions in areas affected by thermal anomalies through the acquisition and processing of TIR images of these areas. These images are systematically acquired by a TIR sensor, stored locally in the RMS, transferred to a central monitoring server, and subsequently processed by the ASIRA analysis software. Each node of the TIR network consists of one RMS, one or two TIR sensors enclosed in a stainless steel housing, and a power supply system (solar panel and battery). The RMS software module is highly flexible, allowing users to configure acquisition schedules, pre-acquisition sensor settings, data transmission methods and timing, and other parameters based on monitoring requirements.

Table 1 outlines the main customizable settings of both the TIR RMS and the ASIRA processing software, which can be adjusted by the user to meet the operational needs. 

The TIR sensor used in this work is the FLIR SC655 camera with a focal plane array (FPA) uncooled microbolometer detector. It has a resolution of 640 × 480 pixels, a spectral range of 7.5–13 µm, an accuracy of ±2 °C, and a thermal sensitivity of <0.03 °C.

### 2.2. The Remote Monitoring Station (RMS)

**Figure 1:**
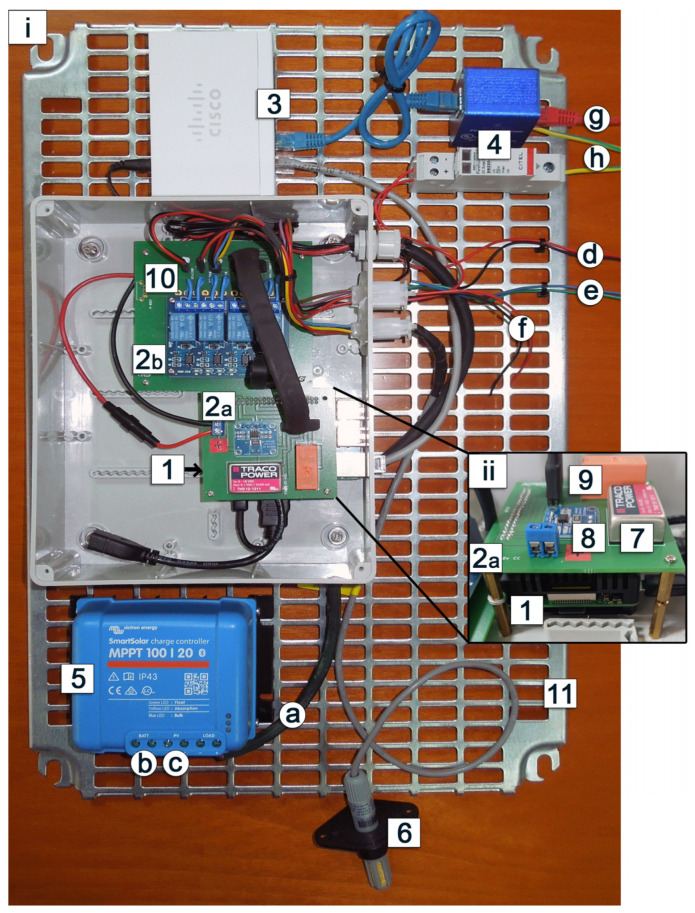
Remote monitoring station (RMS) schematic picture: 1 processing unit (RasPi), 2. power distribution modules, PDM A (2a) and PDM B (2b), 3. LAN switch, 4. surge protection module, 5. power controller, 6. air temperature and humidity probe (T-Hprobe), 7. DC-DC 12-5V converter, 8. INA219 current/power monitor module, 9. RT424012 Relay, 10. 4 Channel Relay Module, 11. fixing stainless steel grid; (a) main power cable, (b) connectors to 12V battery, (c) connectors to solar panel, (d) power wire to IR sensor, (e) power wire to shutter, (f) power wire to LTE/UMTS router, (g) LAN cable to IR sensor, (h) ground wire.

The RMS (Figure 1) is composed of several components: 1. processing unit (RaspPi), 2. power distribution modules (PDM A and B), 3. LAN switch, 4. surge protection module, 5. Solar Charge Controller (SCC), 6. air temperature and humidity probe (T-Hprobe). These components are mounted over a steel grid (Figure 1, label 11) housed inside a polyester case reinforced with fiberglass. The enclosure has an IP66 protection rating, ensuring resistance to atmospheric agents.

The RMS offers the following functionalities:

- Acquisition of TIR images, stored in the processing unit’s memory.
- Acquisition of air temperature and humidity values a few seconds before each TIR image is taken, as well as on an hourly basis; these data are also stored in memory.
- Connection to a server for data upload.
- On-demand acquisition of TIR images and air temperature and humidity values.
- Remote connectivity by LTE/UMTS router or Wi-Fi.

Basically, at scheduled times defined in custom settings, the ICARO software control module activates the TIR camera, LTE/UMTS or Wi-Fi connection, and the air temperature/humidity probe to perform the requested tasks. Once the tasks are completed, ICARO powers down the unused components to save battery energy.

#### 2.2.1. The Processing Unit

The hardware used as the processing unit (Figure 1, label 1) is a Raspberry PI 3 model B (RasPi), featuring a 64-bit quad-core 1.2GHz Broadcom BCM2837 CPU, 1GB RAM, wireless LAN and 100 Base Ethernet, Bluetooth, HDMI® output, four USB ports and 40-pin extended GPIO. The RasPi requires a 5V power supply, which is provided by PDM A mounted over the Raspberry Pi board (Figure 1, label 2). Both the CPU and main chipset are cooled by a passive aluminum heatsink case. The operating system (Raspberry Pi OS) is installed on a 16GB micro-SD card, which also stores the Python-based ICARO (Infrared Camera Automation for Remote Observations) control software. The onboard RasPi GPIO interface (General Purpose Input/Output) is used for communication with the air temperature and humidity probe (T-Hprobe, Figure 1, label 6) and the power distribution modules (PDMs, Figure 1, label 2). The RasPi must be properly configured to (a) connect to a user-defined network by setting WiFi or LAN parameters; (b) allow remote access by SSH and VNC; (c) host web pages using a web server with PHP support; (d) execute Python code by installing Python 3.x and additional required libraries; (e) enable GPIO communication with the other hardware components. During the initial setup, it may be useful to access the Raspberry Pi OS desktop environment via an external HDMI monitor, along with a USB mouse and keyboard.

A detailed guide to the configuration of RasPi is available in Appendix A.

**Figure 2:**
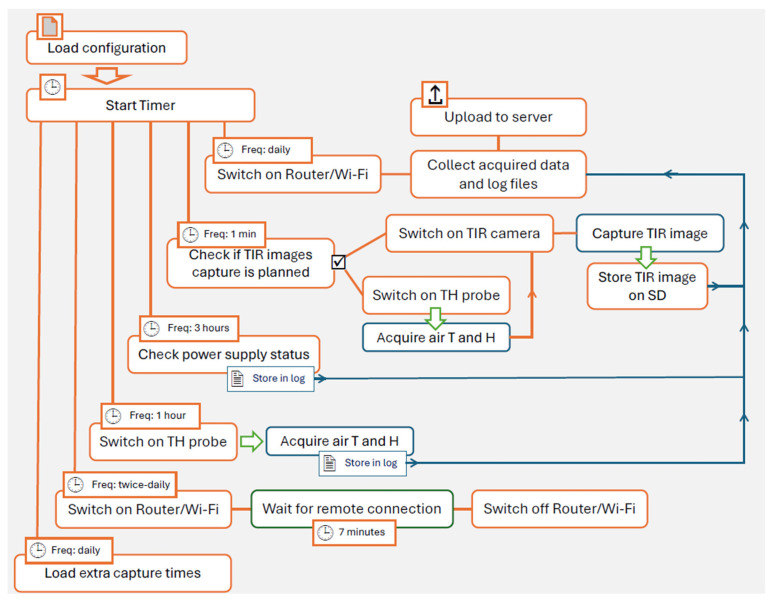
Flowchart of main tasks performed by ICARO, the RMS software control module.

The ICARO Python code is structured to perform primary and secondary tasks at scheduled times, as illustrated in the flowchart in Figure 2.

**Figure 3:**
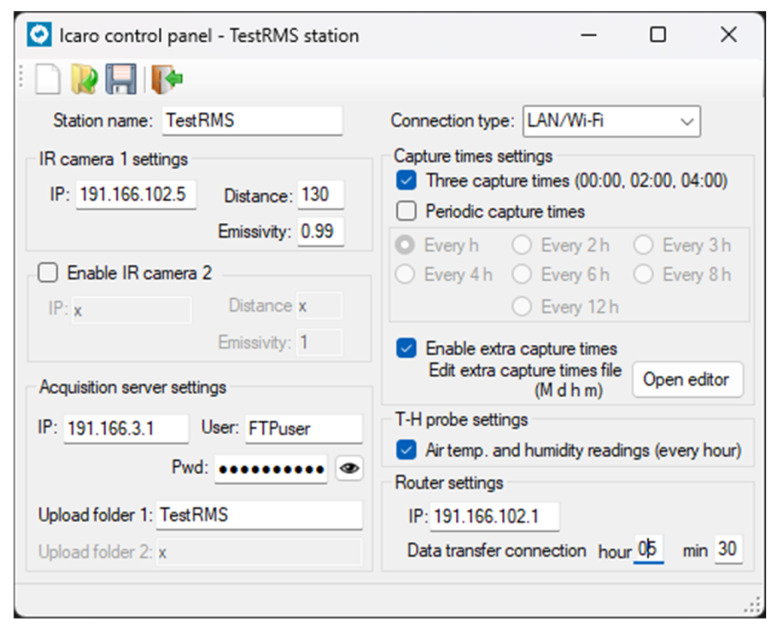
The updateCFG, MS Windows application developed in .Net, used to change parameters stored in the ICARO configuration file.

The RMS configuration can be easily set by using updateCFG, a small MS Windows application developed in .Net, which helps to change all the parameters stored in the ICARO configuration file (Figure 3).

**Figure 4:**
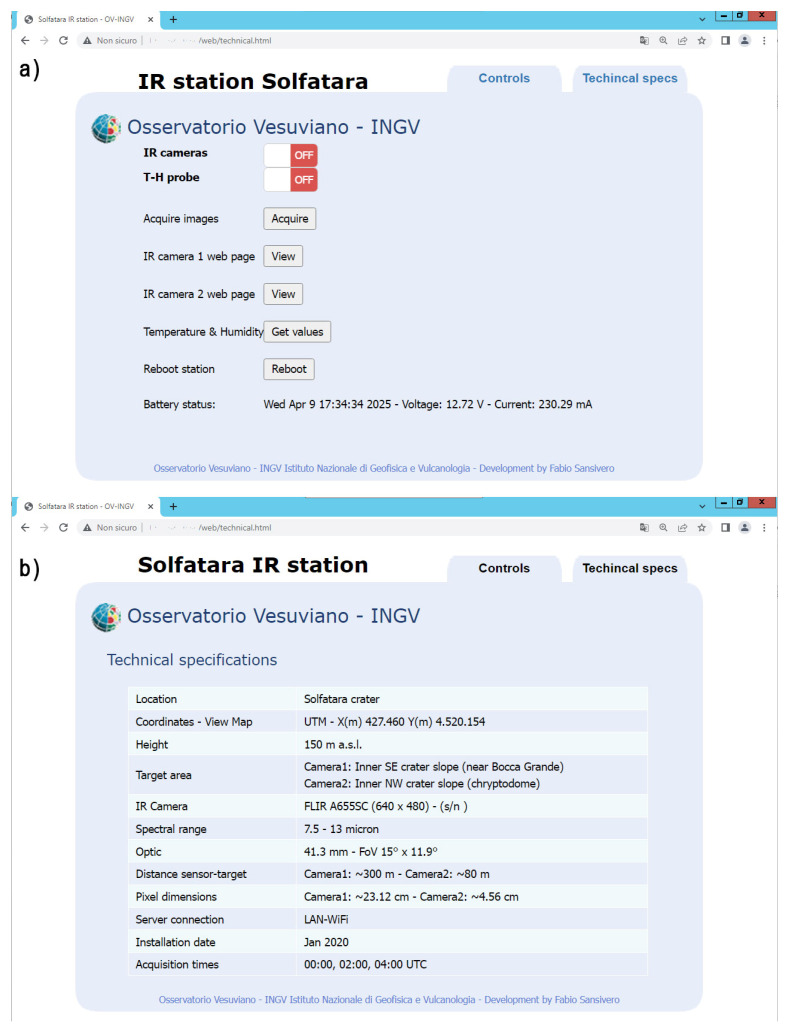
Station’s web page with real-time controls of TIR camera and T-H probe: (**a**) main remote controls; (**b**) hardware technical specifications and details of the installation site and targeting area.

ICARO also includes a basic web server with PHP functionality. If RMS is connected to a network, users can access the station’s web interface (Figure 4) to control real-time TIR image acquisition and air temperature/humidity monitoring, and to upload this data to the server. RMS’s web interface also provides information on system voltage and current consumption monitored by the INA219 module.

Both the ICARO Python code (Icaro.zip), RMS’s web interface files (RMSweb_page.zip), and the .Net updateCFG application (updateCFG.zip) are available in the Supplementary Material. Comprehensive user guides to the ICARO and updateCFG software are available in Appendix B.

#### 2.2.2. The Power Distribution Modules (PDMs)

**Figure 5:**
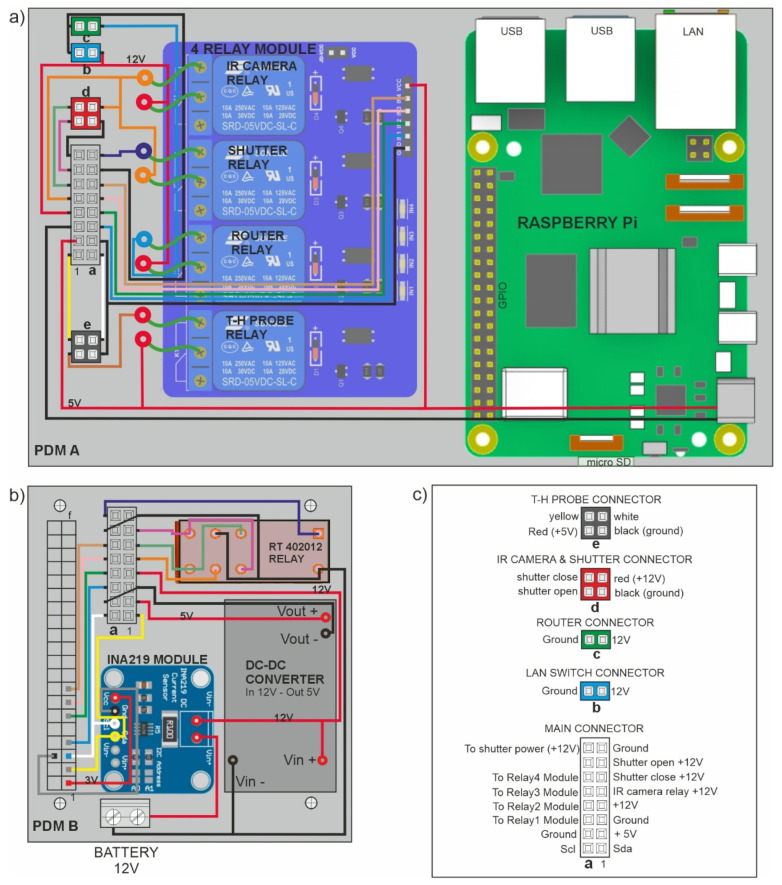
Graphical scheme of PDMs: (**a**) main board (PDM A) hosting Raspberry Pi, 4-relay module, and connectors to RMS hardware components; (**b**) secondary board (PDM B) placed over Raspberry Pi and hosting DC-DC converter, INA219 module, RT402012 Relay, and connectors to PDM A; (**c**) connector pin assignments.

PDMs serve as interfaces between the RasPi and the other system components and peripherals. PDM A (Figure 1, label 2a; Figure 5a) is the main board, which hosts RasPi, the 4-relay module, and connectors to RMS’s devices. PDM B (Figure 1, label 2b; Figure 5b) is mounted over the RasPi’s passive heat sink and is connected to RasPi’s GPIO (connector f, Figure 5b) and to the main 12 V power supply. Figure 5a,b show simplified, non-technical diagrams of PDM A and B circuit layouts, while Figure 5c provides brief descriptions of the connector pin assignments. PDM B includes the following components: 1. A DC-DC converter which steps down 12V to 5V (Figure 1, label 7); 2. An INA219 module, which monitors system voltage and current consumption (Figure 1, label 8); 3. An RT402012 relay (Figure 1, label 9), which manages power polarity inversion for opening and closing the shutter mechanism that protects the TIR camera’s Germanium lens within its stainless-steel housing.

The main connector a (Figure 5b,c) links up PDM B to PDM A, supplying 12 V and 5 V currents and connections to RasPi’s GPIO. The 12 V current is for TIR camera, LTE/UMTS router, LAN switch, and shutter mechanism; the 5 V current supplies RasPi, 5-relays module, and T-Hprobe. The INA219 module (placed on PDM B, Figure 5b) is powered by the 3 V current of RasPi’s GPIO.

RMS needs a 12 V power supply provided by a battery-solar panel system, connected to the SCC (Figure 1, label 5), or by an external AC-DC converter if a power line is available. The 12 V power supply is connected to PDM B and converted by the DC-DC converter (Figure 5b) into a 5 V current, which is required by the RasPi, the 4-relay module, and the T-Hprobe (Figure 5a). The main connector a (Figure 5b,c) transfers 12 V and 5 V currents to PDM B and connects RasPi’s GPIO and RT402012 relay outputs to the 4-relay module, mounted on PDM A. The 4-relay module powers on/off the TIR camera, T-Hprobe, LTE/UMTS router, and Germanium Lens’ shutter, depending on the ICARO software scheduled commands.

#### 2.2.3. The Surge Protection Module (SPM)

RasPi, TIR camera, and LTE/UMTS router are linked by RJ45 LAN cables which are connected to LAN switch (Figure 1, label 3). The surge protection module (SPM; Figure 1, label 4) protects RMS hardware from damage due to lightening through the external LAN cable connected to TIR camera and the external power cable connected to battery and solar panel. The LAN surge arrester should be Class E, fully shielded, Type 2/P1, tested in accordance with CEI EN 61643-21, for universal use according to EN 50173 standards, suitable for all data services up to 57 V DC, and for the protection of 4 wire pairs in data network lines via RJ45 connectors. Nominal discharge current C2 (8/20 µs) total: 10 kA. The 12 V power surge arrester must be based on varistors matched to the RMS voltage; the varistors must be equipped with thermal disconnect devices for end-of-life monitoring. It must be DIN rail compatible and connected in parallel to the line to be protected. Imax: 2 kA. Compliance with IEC 61643-11, EN 61643-11, and UL1449 ed.4.

#### 2.2.4. Air Temperature and Humidity Probe (T-Hprobe)

The T-Hprobe (Figure 1, label 6) we used in this work is a capacitive digital temperature and humidity sensor, model AM2315, with a standard I2C digital bus on the SDA pin, to be connected to the GPIO of the RasPi. Sensor air temperature resolution: 0.1 °C—air humidity resolution: 0.1%RH. Sensor accuracy: temperature: ±0.1 °C—humidity: ±2 %RH. The sensor is placed in a protective enclosure for outdoor use, resistant to weather conditions and harsh environments. The T-Hprobe is connected to RasPi via the GPIO pins SDA (yellow cable-pin3) and SCL (white cable-pin5) and is powered by 5 V current from relay n.4 of the 4-relay module located on PDMA (Figure 5a). The air temperature and humidity values, together with detector-target distance and emissivity, were sent to the FLIR camera to apply the internal proprietary FLIR atmospheric correction algorithm before TIR frame acquisition. 

### 2.3. The Data Processing Software ASIRA

In previous works [25,28], the TIR data-processing automated MATLAB software ASIRA (Automated System of InfraRed Analysis) was described in detail. It was developed by the TIRLab of INGV-Osservatorio Vesuviano and is currently used to monitor surface temperature changes in diffuse degassing volcanic areas as requested by the agreement between the National Civil Protection and INGV. 

In this work, a reviewed version of ASIRA, developed for GNU Octave free scientific programming language, is presented, and its code is available under the Creative Commons Attribution 4.0 International License as Supplementary Material (ASIRA.zip). The availability of ASIRA code in the GNU Octave free language (www.octave.org) can allow anyone to use this data processing software independently of the ownership of the expensive commercial software. 

**Figure 6:**
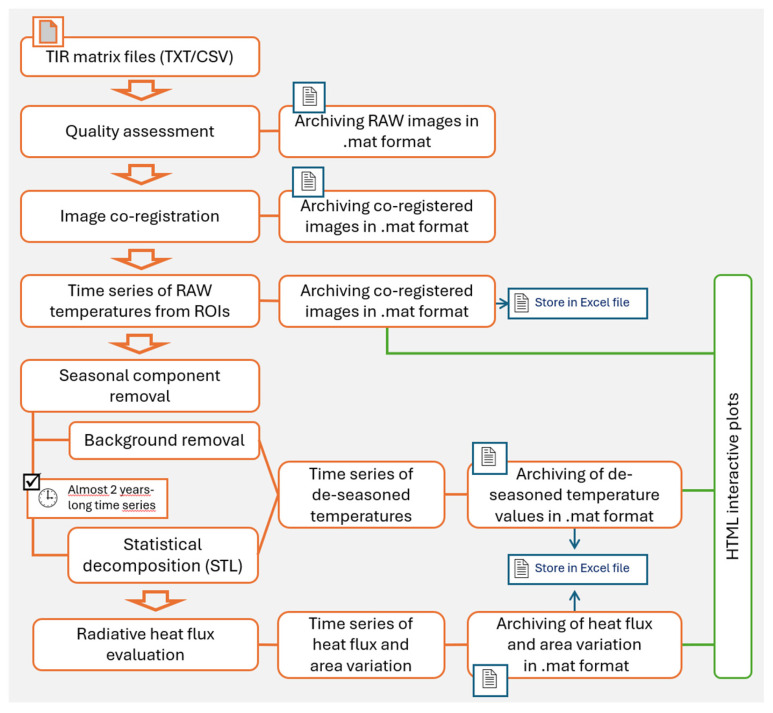
Flowchart of main tasks and products of the data processing software ASIRA (Automated System of InfraRed Analysis).

**Figure 7:**
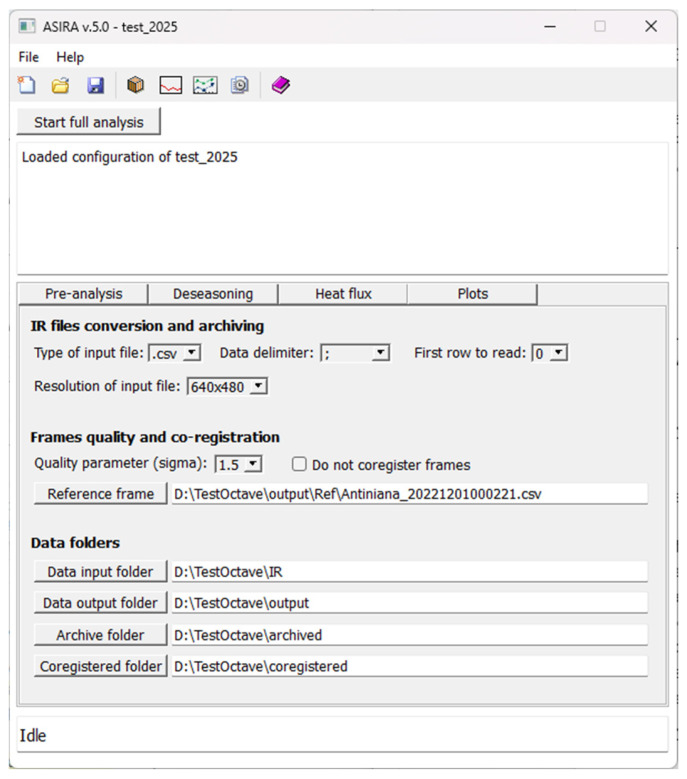
ASIRA graphic interface. A comprehensive user manual of ASIRA, with a detailed description of ASIRA’s graphic interface functions and commands, is available as Supplementary Material.

A flowchart of the functionalities of this modular program, developed with a user-friendly graphical interface, is illustrated in Figure 6. In Figure 7, a screenshot of the ASIRA graphic interface is shown. The technical user manual of ASIRA is available as Supplementary Material.

The ASIRA processing workflow consists of the following main steps: (1) quality assessment and exclusion of low-quality TIR frames and archiving of TIR input data, provided as .txt or .csv matrix, in .mat format files, split in years; (2) co-registration of all frames with respect to a reference frame to correct possible pixel misalignments; (3) extraction of continuous time series of apparent temperatures (both maximum and average values) from user selected areas (ROIs) within the TIR frames; (4) removal of seasonal components from the temperature time series via background removal or statistical decomposition methodologies; and (5) evaluation of radiative heat fluxes and area variations in thermal anomaly.

#### 2.3.1. Quality Assessment of TIR Frames

The exclusion of low-quality TIR frames is a necessary step before starting to process data. Heavy rainfall, fog, or water vapor from diffuse degassing areas may sometimes heavily degrade TIR frame quality. To filter out these low-quality data, only IR scenes meeting the following condition were selected [28]:

$${\sigma F}_{i}>m\sigma -c\;*\;{\sigma F}_{\sigma }$$

where *σF_i_* is the standard deviation (SD) of the *i*-th TIR frame, *mσ* is the median of the SD values across all TIR frame time series, *σF_σ_* is the standard deviation of all the frame SDs, and *c* is a user-defined coefficient based on the statistical distribution of the data. A value of *c* = 1 was found to be appropriate for obtaining a homogeneous dataset, which excludes frames of very low quality.

#### 2.3.2. Co-Registration of TIR Frames

The co-registration of TIR frames is essential if the TIR sensor is affected by small displacements, which can be caused by continuous ground movements, typical of active volcanic areas, or by instability of the RMS site. Co-registration is performed through the Efficient subpixel image registration algorithm code [40], which ensures pixel-to-pixel matching between frames across the entire time series. This code gives the same precision as the fast Fourier transform (FFT) upsampled cross correlation in a small fraction of the computation time and with reduced memory requirements. It obtains an initial estimate of the cross-correlation peak by an FFT and then refines the shift estimation by upsampling the discrete Fourier transforms (DFTs) only in a small neighborhood of that estimate by means of a matrix-multiply DFT [38].

#### 2.3.3. Seasonal Component Removal

One of ASIRA’s outputs is a time series of maximum surface temperatures corrected for seasonal variability, achieved through the application of two different methodologies depending on the length of the TIR time series. Removing the seasonal component is a critical step, as it isolates endogenous thermal anomalies from external exogenous effects. If the TIR time series length is less than two years, only the Background Removal Procedure (BKRp) can be applied. If the time series length is higher than two years, the STL (Seasonal and Trend decomposition using Loess) algorithm [41] can also be applied. 

##### The Background Removal Procedure

**Figure 8:**
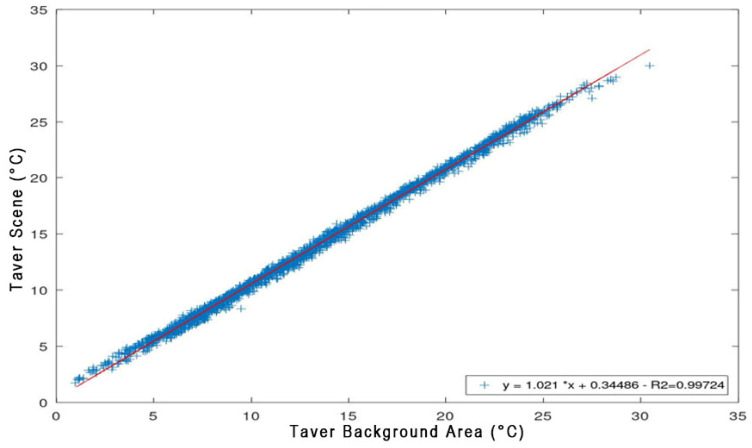
Example of average temperature of TIR scene vs. average temperature of background area plot. Linear correlation is very good with R near 1.

The BKRp involves the removal of the background temperature values from the raw TIR frame time series. Background temperatures are extracted from a specific area (background area, BKG) within the IR scene that was not influenced by any thermal anomalies. The BKRp procedure is based on the observed linear relationship between the mean (or maximum) temperature of BKG (TaverBKG) and the mean (or maximum) temperature of the entire IR scene (TaverSc), as illustrated in Figure 7 (TaverSc vs. TaverBKG plot). If BKG is correctly chosen, the linear correlation is very good and enables the application of the following Equation [28]:

$$dT\left(n\right)=T_{aver}Sc\left(n\right)-T_{fit}\left(n\right)$$

where *dT*(*n*) represents the residual deseasoned temperature value at the *n*-th TIR scene, *T_aver_Sc*(*n*) is the average temperature of the *n*-th IR scene, and *T_fit_*(*n*) is the corresponding fitted temperature value. *T_fit_*(*n*) is obtained through the linear regression model (Figure 8) established between the average background temperature *T_aver_BKG*(*n*) and the average scene temperature *T_aver_Sc*(*n*).

The wise selection of BKG is critical for ensuring the effectiveness of the procedure. The BKG must be located outside regions influenced by thermal anomalies, exhibit similar lithological characteristics to the anomaly area, and be free from vegetation and anthropogenic features. An efficient method to assess the quality of the selected BKG was to perform a linear regression analysis on the time series of average background temperatures.

**Figure 9:**
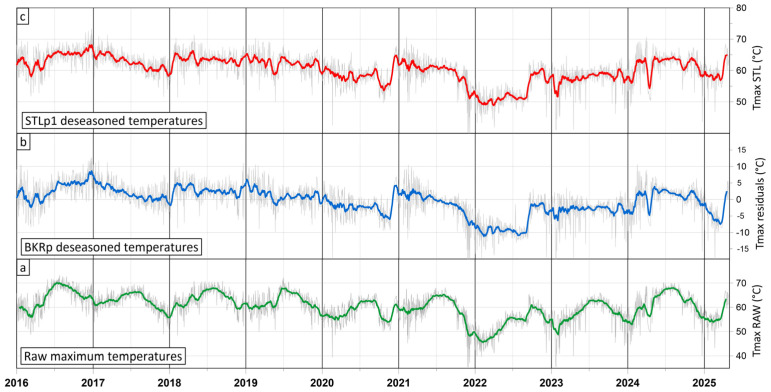
Comparison between the raw maximum temperatures plot (**a**) from a thermal anomaly area of Campi Flegrei volcano (Italy) and the deseasoned residual temperatures plot after applying the BKRp procedure (**b**) and the STLp1 procedure (**c**). Green, blue, and red colored lines are the running average (window = 21) of processed data (light gray lines).

Plots of both the raw temperature time series and the deseasoned residual temperature time series generated by applying the BKRp methodology are reported in Figure 9. In this figure, the BKRp-deseasoned temperatures plot (Figure 9b) better evidences the thermal anomaly, which is partially hidden by the seasonal component (Figure 9a).

##### The STL (Seasonal and Trend Decomposition Using Loess) Procedures

If the TIR time series length is higher than two years, the STL algorithm [41] can be applied, and two distinct STL-based procedures are executed by ASIRA as described in [28]. 

The first procedure (STLp1, [25]) applies the STL algorithm to time series of raw maximum (or average) temperature values extracted from user-defined Regions of Interest (ROIs) within TIR frames. Time series of temperature raw data is then factored into three components as follows:

*TmaxTS = TrendTS + SeasonTS + RemainTS*

where *TrendTS* is the smoothed general trend, *SeasonTS* captures the periodic variations linked to exogenous factors, and *RemainTS* contains the residuals. By combining the *TrendTS* and *RemainTS* components, a deseasoned maximum temperature time series (*TmaxSTLTS*) is obtained (Figure 9c).

The second procedure (STLp2, [28]) involves the removal of seasonality from the entire TIR frames. STL decomposition is applied to average (or maximum) temperature time series from a background area (BKGTS), yielding three different time series: BKGTrendTS, BKGSeasonTS, and BKG RemainTS, as previously discussed. The values of BKGSeasonTS are subtracted from the temperature time series of all the pixels of the TIR frames, generating new TIR frames whose seasonal component is removed. This procedure is necessary as it enables the extraction of radiative heat fluxes from user-defined ROIs characterized by thermal anomalies inside deseasoned TIR frames whose temperatures are exclusively linked to endogenous processes.

#### 2.3.4. Radiative Heat Flux Estimation

If TIR time series are almost two years long, it is possible to estimate the thermal energy radiated per unit area and per unit time (radiative heat flux) by processing the deseasoned TIR matrices created with the STLp2 procedure. Time series of radiative heat flux (HFluxTS) are determined by applying the Stefan–Boltzmann law to the selected pixels within the user-defined ROIs of areas characterized by thermal anomaly within the TIR frames. ROIs are typically thermally heterogeneous, containing both high-temperature sources and lower-temperature backgrounds, which leads to underestimate the radiative heat flux value. To address this, the pixel selection of ROI is based on a threshold criterion where only high-emittance pixels with temperatures exceeding two standard deviations above the mean of the TIR image are considered.

Once the pixel selection within the ROI has been made, it is possible to proceed with the estimation of the ROI’s radiative heat flux (HFluxROI, W/m^2^) by using the Stefan–Boltzmann equation:

$${HFlux}_{ROI}=A\sum_{i=1}^{n}\sigma \varepsilon {\left({{Tmax}_{ROI}}_{i}\right)}^{4}$$

where *σ* is the Stefan–Boltzmann constant, *ɛ* is the emissivity (for pyroclastic rocks = 0.9), *A* is the investigated area surface (m^2^), *Tmax_ROIi_* is the maximum temperature of the ROI within the *i*-th TIR scene, and n is the length of the TIR time series. 

#### 2.3.5. Area Variations in Thermal Anomaly

To monitor variations in the spatial extent of thermally anomalous areas, time series of the number of ROI pixels, whose temperatures exceed the threshold criterion used for radiative heat flux estimation, are generated. Temporal trends in the high-emittance pixel count provide additional quantitative information on the spatial dynamics of the surface thermal field, offering a complementary perspective to the analysis of temperature and heat flux variations.

#### 2.3.6. Output Files

Almost all the processed data are saved in binary format (MATLAB-compatible .mat files) during the different phases of the analysis performed by GNU Octave. Raw, co-registered, and STL-deseasoned time series of TIR matrices are saved along with extracted temperatures and heat flux values in .mat files split per year. The temperature values resulting from the TIR frame analysis are also saved in an Excel file after subdividing them into different sheets. The availability of temperature data in an Excel file ensures seamless integration with third-party visualization and analysis tools that query Excel datasheets. Co-registered TIR matrices are also exported in CSV format in a user-defined folder. 

Technical details on the specifications used to save the processed data are available in the ASIRA user manual as Supplementary Material.

#### 2.3.7. HTML Interactive Plots

**Figure 10:**
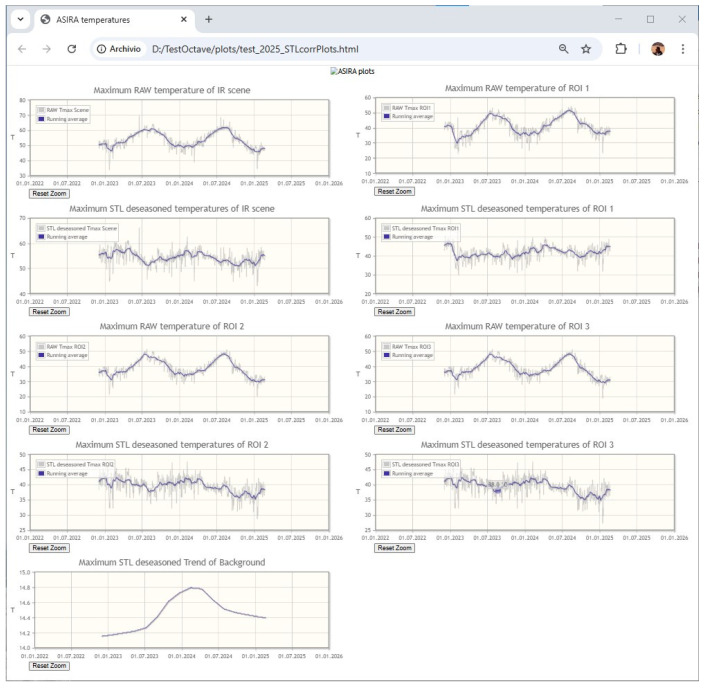
ASIRA HTML interactive plots. By passing with mouse pointer over the plot, the temperature and the date are shown. A zoom function is also available.

As the final output of the processing workflow, several HTML plots can be generated on demand. ASIRA users can set the start and end times of the plot and the plot type: BKGp- or STL-deseasoned temperatures, heat flux, and area variation values (Figure 10). HTML plots can be viewed with any Internet Browser and are made with the jqPlot libraries, a jQuery plugin to generate pure client-side JavaScript charts in web pages (www.jqplot.com; Copyright 2009–2013 Chris Leonello). jqPlot libraries are available for use in all personal or commercial projects under both the MIT and GPL version 2.0 licenses.

## 3. Testing of RMS and Data Processing Software

### 3.1. Field Testing of RMS Hardware and Software

Prototypes of the proposed hardware and software components of RMS have been extensively field-tested over several years in diffuse degassing areas at Campi Flegrei and Vesuvio volcanoes (Italy). The current version of RMS, described in this work, incorporates substantial enhancements in both performance and functionality, suggested by field experience that revealed several limitations in the original prototype. These insights guided key modifications to both hardware and software subsystems.

One of the most critical parameters identified during testing was the reliability of the RMS power supply, particularly when operating with a solar panel and battery. 

Unexpected system shutdowns due to power system inefficiencies were addressed and resolved by integrating an INA219 module (Figure 1, label 8 and Figure 5b), which enables real-time monitoring. The ICARO software module can send automated email alerts if the voltage drops below a predefined threshold. Additionally, the INA219 module detects abnormal power consumption patterns associated with hardware degradation—such as faulty wiring or electronic components—allowing preventive maintenance before system failure occurs.

Energy consumption was further optimized through improved hardware connections (see the electrical diagrams in Figure 5) and the implementation of power management procedures for peripheral devices (e.g., TIR camera, LTE/UMTS router, and T-Hprobe) when not in use. New code developed within the ICARO software module manages the 4-relay module, enabling the selective powering on/off of RMS components. This approach minimizes unnecessary energy usage while maintaining operational readiness. Table 2 summarizes power consumption measurements for the RMS during typical tasks.

As shown in Table 2, the TIR camera represents the most power-demanding component. Continuous or high-frequency image acquisition is feasible only when the RMS is connected to the main power grid. Under solar-powered operation, two options exist: reduce acquisition frequency or replace the TIR camera with a less advanced, lower-power model. For example, the FLIR VUE Pro R 640 TIR camera was evaluated as a viable low-power alternative. This compact camera, commonly used in UAV applications, operates at an input voltage between 4.8 and 6.0 V and has a tested power consumption of approximately 2 W—lower than that of the FLIR SC655 camera, which consumes over 2.8 W. However, the VUE Pro R offers lower performance in terms of accuracy and thermal sensitivity compared to the SC655. To avoid compromising data quality, a "mixed configuration" was tested. In this setup, the VUE Pro camera was used for continuous acquisition, while the SC655 camera captured a limited number of TIR frames during nighttime. This approach generated two distinct TIR time series, which were processed separately, providing both a high-quality and a continuous dataset.

The preliminary results from this short test were promising and suggested that the mixed configuration could be a feasible solution for managing high-frequency image acquisition when solar panels are used as the power source.

### 3.2. Testing of ASIRA for Octave Data Processing Software

The ASIRA software presented in this study is a revised and optimized version of the MATLAB-based ASIRA code originally described in [28], now adapted for the open-source scientific programming language GNU Octave. Rather than a simple language translation, this new version includes substantial improvements in computational structure, code optimization, and graphical user interface (GUI), along with new functionalities currently in use for volcanic monitoring by INGV–Osservatorio Vesuviano. 

**Figure 11:**
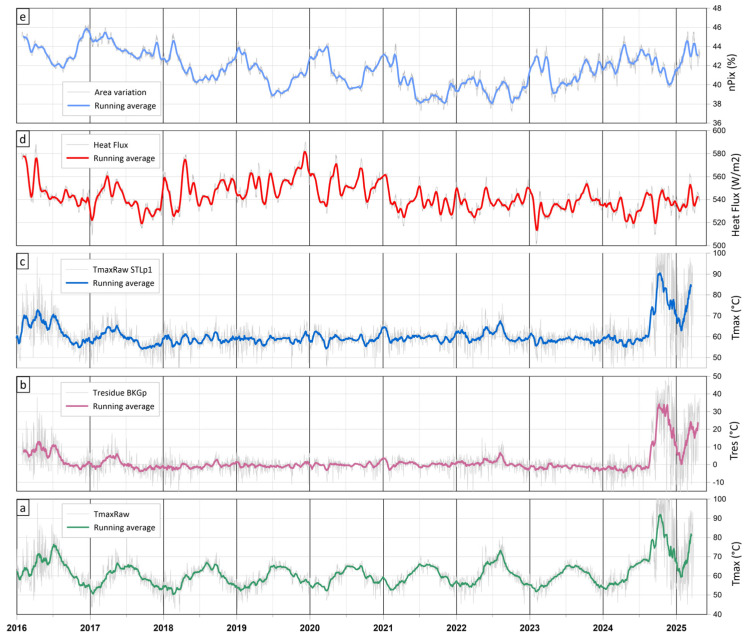
Plots of the main results after ASIRA processing of TIR time series: (**a**) maximum raw temperatures of TIR scene; (**b**) residual values of maximum temperatures of TIR scene deseasoned by applying BKRp; (**c**) maximum temperatures of TIR scene deseasoned by applying STLp1; (**d**) heat flux values of user-defined ROI expressed in W/m^2^; (**e**) area variations within user-defined ROI expressed in percentage of high-emittance pixels.

To evaluate both computational performance and output reliability, a ten-year TIR time series acquired by an RMS monitoring the Solfatara volcano (Campi Flegrei, Italy) was processed using ASIRA for Octave. Figure 11 displays the principal output plots from TIR data processing using ASIRA.

The STLp1-deseasonalized time series (Figure 11c) reveals significant thermal fluctuations associated with diffuse degassing and shallow reactivation processes. Radiative heat fluxes (Figure 11d) allow for the quantification of thermal energy release over time within the user-defined ROI. The use of statistical thresholds (exceeding two standard deviations above the scene mean) enabled the sensitive detection of high-emissivity pixels, thereby enhancing the reliability of thermal anomaly assessment. Additionally, tracking changes in the number of above-threshold pixels within each ROI over time (Figure 11e) allows for evidence of spatial variations in anomaly extent.

**Figure 12:**
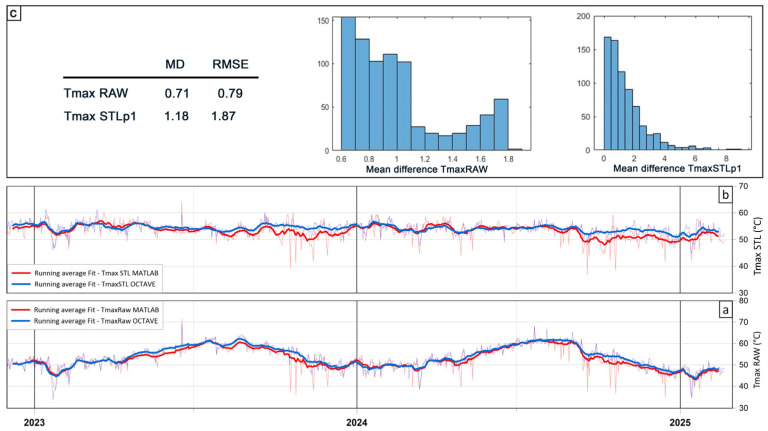
Plots of maximum raw temperatures ((**a**); TmaxRaw) and maximum STLp1-deseasoned temperatures ((**b**); TmaxSTL) resulted by ASIRA for Octave and ASIRA for MATLAB. Blue lines are for OCTAVE-processed data; red lines are for MATLAB-processed data. (**c**) histogram of Mean Difference between MATLAB- and OCTAVE-processed data (TmaxRaw and TmaxSTL) with the related table of Mean Difference (MD) and Mean Square Error (RMSE).

The same dataset used to generate Figure 11’s plots was also processed using the original MATLAB version of ASIRA for comparison. Plots Figure 12a,b compare the results obtained with the Octave and MATLAB versions, respectively. Slight differences are observable between the two outputs (blue line for Octave and red line for MATLAB). Figure 12c presents histograms of the differences between the outputs, along with calculated Mean Difference (MD) and Root Mean Square Error (RMSE) values for both the raw maximum temperatures and STLp1-deseasonalized temperatures.

The results of this comparison test also showed that ASIRA for Octave performs slightly slower than its MATLAB counterpart, requiring approximately 1.2 times longer to complete the analysis. This discrepancy is primarily attributed to differences in the efficiency of underlying software libraries, as GNU Octave is generally less optimized than MATLAB. Furthermore, some algorithms—such as image co-registration—differ in implementation between the two platforms, which may further influence execution time, penalizing GNU Octave.

The differences in temperature trends from GNU Octave and MATLAB output data, which can be observed in Figure 12a,b plots, can be attributed to several factors, including differences in co-registration algorithms, in background area selection, and in scripting or library behavior between Octave and MATLAB. Nonetheless, these discrepancies fall within the expected uncertainty associated with TIR data acquisition and are not considered significant in a monitoring context.

## 4. Discussion

The system’s effectiveness has been validated in active volcanic areas of southern Italy, demonstrating high reliability in detecting anomalous thermal behavior and distinguishing endogenous geophysical processes from exogenous signals. 

Thanks to its modular architecture, low cost, and open-source software, the hardware configuration is specifically designed for a permanent TIR network. It represents a scalable and replicable solution for environmental monitoring in high-risk scenarios, contributing to both hazard mitigation and geophysical/environmental research.

The technical solutions adopted to optimize costs and performance have proved to be robust and reliable in the field. The main limitation of the system lies in its power supply system, which, in remote installations, necessarily depends on solar energy. Therefore, the solar panel and battery setup must be expertly designed to meet the specific power demands of the remote station, which may vary significantly depending on multiple factors.

The proposed system for acquiring TIR images is subject to various sources of uncertainty and error. While some of these have been addressed and resolved, others are intrinsic to the system and must be considered when interpreting the final processed data.

Table 3 lists the main sources of uncertainty and error, along with the corresponding mitigation solutions.

Regarding the ASIRA software package for Octave, Table 4 presents a comparison with the functionalities available in the MATLAB version of ASIRA. As shown, the Octave version includes more functionalities than the MATLAB version. However, MATLAB libraries are generally faster, and its co-registration toolbox (which uses the ECC image alignment algorithm [42]) offers better accuracy due to the implementation of a ‘warping functionality’ not available in the Octave counterpart (Efficient Subpixel Image Registration Algorithms, [40]).

As also indicated in Table 4, automation features have not been implemented in ASIRA for Octave, as these can vary greatly depending on the intended use of the system. Implementing all possible usage scenarios would have been overly complex. Users can, however, add custom automation scripts tailored to their specific needs.

The entire system is under continuous development, with new technical implementations and data processing solutions being refined. Some of the ongoing and future improvements include the following:

- Integration of machine learning techniques for the enhanced detection of subtle thermal anomalies.
- Fusion of TIR data with seismic, geochemical, ground deformation (GPS), and infrasound measurements to create multi-parameter, real-time volcanic monitoring frameworks.
- Development of automated data processing methods for the detection of changes in thermal anomalies.
- Implementation of real-time alert systems using AI and image recognition technologies.
- Enhancement of wireless communication (e.g., via satellite or 5G) to enable fast, real-time remote data transfer from inaccessible areas.
- Improvement of power systems through more efficient solar panels and advanced battery management components.
- Enabling robotic repositioning or sensor adjustment for optimized TIR data acquisition.
- Refinement of the modular design of RMS to allow rapid deployment, expansion, or relocation in response to evolving monitoring needs.
- Development of lightweight, portable RMS units for temporary field campaigns or mobile surveys.

## 5. Conclusions

The development and implementation of a comprehensive, modular system for the remote acquisition and analysis of thermal infrared (TIR) images, as described in this work, represent a significant opportunity to study surface temperature anomalies across a range of environmental and geophysical contexts. The remote monitoring station (RMS), centered on a Raspberry Pi-based architecture, exhibits a robust, low-power, and flexible design capable of acquiring high-resolution TIR images and correcting them using atmospheric parameters (air temperature and humidity) in remote and harsh environments. 

The integration of custom Python software (ICARO) and modular hardware components ensures operational efficiency, ease of maintenance, and adaptability to varying monitoring requirements. For TIR data analysis, the new open-source version of the ASIRA software—now developed for the free GNU Octave platform—is proposed as an accessible and customisable alternative to the previous MATLAB counterpart. The restructured ASIRA includes a comprehensive suite of functionalities: data quality control, frames co-registration, seasonal component removal, radiative heat flux estimation, analysis of thermal anomaly area variations, and output generation in the form of HTML interactive plots. This transition from proprietary (MATLAB) to open-source (GNU OCTAVE) software enhances accessibility and reproducibility for a wider scientific community.

The dual approach implemented for deseasoning temperature time series—based on both background removal and Seasonal–Trend decomposition using Loess (STL)—enables the reliable analysis of both short and long time series. This ensures the effective separation of endogenous thermal signals from seasonal exogenous components, which is particularly critical in volcanic and geothermal monitoring, where accurate detection of subsurface thermal activity helps in hazard assessments. Moreover, the methods introduced for the spatial and temporal tracking of thermal anomalies and heat fluxes also provide valuable tools for understanding the dynamics of surface thermal processes. 

A detailed RMS configuration guide (Appendix A), along with a comprehensive ASIRA user manual provided as Supplementary Material, enables us to understand the system architecture in depth and effectively manage both hardware and software components.

Thanks to its modular design and low power consumption, the system is well suited for deployment in remote or extreme environments, supporting long-term autonomous monitoring. It can be customized in acquisition times, threshold-based anomaly alerts, and integration with remote data transmission protocols, making it especially suitable for data-scarce or logistically challenging areas.

Potential fields of application of the system include the following:

- *Geothermal energy*: Monitoring and assessment of geothermal fields through the mapping of thermal anomalies indicative of subsurface fluid dynamics or reservoir changes.
- *Landslide and rockfall hazard assessment*: Detection of subtle thermal variations potentially associated with moisture changes or incipient mass movement in unstable slopes.
- *Permafrost and glacier studies*: Observation of thermal dynamics in periglacial environments to assess thawing trends or detect basal ice melt.
- *Infrastructure and pipeline monitoring*: Detection of thermal leaks or abnormal heat signatures along pipelines and industrial infrastructure.
- *Agricultural and forestry management*: Evaluation of plant water stress, canopy temperature variability, or early detection of wildfires in unmanaged forested regions.

Overall, the system demonstrates considerable potential for long-term deployment in multi-disciplinary environmental monitoring frameworks. Future improvements may focus on integrating additional sensor types, refining automated anomaly detection algorithms, and expanding data visualization capabilities to further enhance early warning capacities and scientific insight.

## Figures and Tables

**Table 1:** Main customizable settings of TIR network.

Task	Available Settings
RMS—Acquisition	Number of TIR captures per day
	Customized regular capture times
	Customized extra capture times
RMS—Sensor	Customized communication strings
	Customized pre-acquisition correction parameter strings
	Number of sensors to manage (1 or 2)
RMS—Transmission	Hardware type (LAN, Wi-Fi, or LTE/UMTS router)
	Central acquisition server parameters
	Timing of frame upload to the server
RMS—T-H Probe	Timing of air temperature and humidity measurement by the TH probe
RMS—Power	Power supply switch-on/-off times of the sensor
	Power supply switch on/off of the transmission module
	Power usage and state monitoring with alerts
ASIRA	Processing task type and custom parameters for analysis
	Setting of the user-defined region of analysis (ROI)
	Output data type
	Output plot type

**Table 2:** Power consumption of RMS and devices during different operational tasks.

Device	Power Consumption (mA)
RMS standby mode (LAN on)	213
Wi-Fi on	+9.4
T-Hprobe on	+32
RMS capturing mode (LAN+T-Hprobe+TIR camera on)	480 (peak) *
RMS in FTP uploading mode (LAN+Router LTE/UMTS on)	326 **

* referred to FLIR SC655 camera; ** referred to generic LTE Router.

**Table 3:** List of the main sources of uncertainty and error during TIR image acquisition.

Source Uncertainty and Error	Fixed			Solution
	Yes	Not	Partially	
**Sensor calibration**—TIR cameras, if not perfectly calibrated, lead to inaccurate temperature readings.	✓			Periodic calibration
**Sensor drift**	✓			Periodic calibration/replacement
**Gas emissions**—intense water vapor, CO_2_, and other gases can absorb infrared radiation, altering T values.			✓	ASIRA quality control of TIR images excludes the most degraded ones
**Bad weather conditions**—fog, rain, and airborne particulates can significantly scatter or absorb infrared radiation.	✓			ASIRA quality control of TIR images excludes degraded ones with a high level of precision
**Ambient temperature and humidity**	✓			Values sent by ICARO to the TIR camera if it comes with an internal atmospheric correction algorithm (FLIR)
**Emissivity**—incorrect assumptions on the emissivity of target materials can lead to significant errors in temperature estimation.	✓			Emissivity sent by ICARO to the TIR camera if it comes with an internal correction algorithm (FLIR)
**Sensor–target distance**	✓			Distance value sent by ICARO to the TIR camera if it comes with an internal correction algorithm (FLIR)
**Spatial resolution limits**—small thermal anomalies may be unresolved when the sensor–target distance is high.		✓		
**Viewing angle**—high angles of the target surface introduce distortion and may affect apparent temperature and heat flux estimation.		✓		
**Parallax and terrain-induced distortion**—topographic relief can lead to misregistration or displacement in images.		✓		
**Temporal variability**—thermal rapid changes can make single snapshots potentially misleading.	✓			ICARO can be configured to capture TIR frames with the most suitable time frequency
**RMS site instability**—ground motion or instability of the installation site can lead to misregistration or displacement in images.	✓			ASIRA co-registration of TIR frames allows the optimal pixel alignment

**Table 4:** List of functionalities implemented in ASIRA for MATLAB and ASIRA for Octave.

Functionality	ASIRA for MATLAB	ASIRA for Octave
Graphic user interface (GUI)	✓	✓
Free availability of ASIRA code (CC by 4.0)	✓	✓
Free scientific programming language		✓
Cross-platform software (Windows, macOS, Linux)	✓	✓
Code optimized for parallel computing		✓
Code optimized for low-memory PC		✓
Automation	✓	
Image co-registration	✓	✓
Quality assessment of TIR images	✓	✓
Archiving RAW and co-registered TIR images as a .mat file	✓	✓
Analysis is also available for user-selected areas (ROIs)	✓	✓
Seasonal removal for time series shorter than 2 years	✓	✓
Seasonal removal for time series longer than 2 years	✓	✓
Radiative heat flux evaluation	✓	✓
Area variations in thermal anomaly		✓
Data output in Excel sheets	✓	✓
Data output as HTML interactive plots		✓

## Data Availability

To test ASIRA functionalities, raw TIR data from the stations of the permanent thermal infrared surveillance network of INGV-Osservatorio Vesuviano are available under a Creative Commons Attribution 4.0 International License at: https://www.ov.ingv.it/ov/thermolab/index_eng.html (accessed on 20 April 2025).

## Supplementary Materials

The following supporting data can be downloaded at https://www.mdpi.com/article/10.3390/s25134141/s1; Software: ICARO, ASIRA, updateCFG. Document: ASIRA user guide.

## Appendix A. RMS Configuration Guide

### Raspberry Pi OS Setup

The RasPi must be connected to a monitor via HDMI and to a mouse and keyboard via USB. Raspberry Pi OS must be installed on the micro-SD by using the Raspberry Pi Imager utility. Insert a micro-SD in the RasPi slot and power on the device. During the first boot, set the user login and password, and then connect to the internet via Wi-Fi using the graphical interface of Pi OS. Set the current date and update the system by typing the following:

sudo apt-get update

sudo apt-get upgrade

sudo pip3 install --upgrade setuptools

It will take a while. After reboot, the following task must be executed:

- Set up LAN or Wi-Fi parameters depending on the type of connection chosen for RMS by using the graphical interface of Pi OS or by modifying the file /etc/dhcpcd.conf.
- Enable SSH and VNC by using raspi-config: sudo raspi-config.
- The remote access to RasPi can be performed via SSH console with the PuTTY free utility or with VNC software. To easily manage files on RasPi, the use of an FTP client software (for example, the FileZilla free app) is kindly suggested. To use SSH, VNC, and FTP, it is necessary to define the root password: sudo passwd root, and then type the password.
- Install Apache web server: sudo apt-get install apache2-y.
- Add the line: “ServerName localhost” to the file /etc/apache2/apache2.conf.
- Restart web server: sudo service apache2 restart.
- Authorize FTP writing privileges to the web folder by typing the following:

sudo groupadd upload add the group upload

enter su command and then

sudo chown root.upload /var/www/html/

sudo chmod 775 /var/www/html/

sudo adduser pi upload add pi user to upload group

sudo chown -R pi:www-data /var/www change permission to www folder.

- Install PHP interpreter: sudo apt-get install php libapache2-mod-php.
- Customize RMS web page (RMSweb_page.zip) with station name and technical specs, and then copy the files in the folder: ‘/var/www/html/.
- Enable GPIO interfaces in the raspi-config Interface Option section:
  *enable/disable automatic loading of SPI kernel module > YES*
  *enable/disable automatic loading of I2C kernel module > YES*
  *enable/disable remote access to GPIO pins > YES*
- Install Pi OS additional libraries:
  sudo apt-get install build-essential tk-dev libncurses5-dev libncursesw5-dev libreadline6-dev libdb5.3-dev libgdbm-dev libsqlite3-dev libssl-dev libbz2-dev libexpat1-dev liblzma-dev zlib1g-dev libopenjp2-7 libtiff5 libffi-dev libjpeg-dev bluetooth libbluetooth-dev –y

- Install Python 3.10:
  sudo wget https://www.python.org/ftp/python/3.10.6/Python-3.10.6.tgz
  tar xzvf Python-3.10.6.tgz
  cd Python-3.10.6
  sudo ./configure --enable-optimizations
  sudo make -j 4
  sudo make install

- Install Python additional libraries:
  sudo pip3 install RPI.GPIO RasPi’s GPIO libraries
  sudo pip3 install adafruit-circuitpython-am2320 T-Hprobe libraries
  sudo pip3 install Adafruit-Blinka T-Hprobe libraries
  sudo pip3 install pi-ina219 INA219 libraries

## Appendix B. ICARO Software Functionalities

### Appendix B.1. Installation on RasPi

Create the folder *home/pi/icaro* by using an FTP client to install ICARO in the RasPi microSD. Then copy into this folder the content of the zipped file “*Icaro_v.4.5.zip*” available as Supplementary Material of this work. Remember to set the file permission to *executable*. Finally, to execute Icaro at every RasPi boot, the following line must be added to the file */etc/rc.local* (just before the raw containing the command exit 0):

python3 /home/pi/Icaro/watch.py &  P.S. don’t miss & char

### Appendix B.2. Functionalities

- icaro.py—Python script that performs all main tasks at scheduled times after loading the configuration. In detail:

- Execute the script *IRacq.py* at scheduled times to perform TIRs capture. If the RMS connection is by LAN or Wi-Fi, the TIR images are uploaded by FTP to the acquisition server at the same time as capture. If the RMS connection is by an LTE/UMTS router, the TIR images are all uploaded once a day at the scheduled time, after establishing the router connection. The user can customize TIR image capture times by setting the configuration with the *updateCFG* software and by enabling extra capture times and adding capture times in the file *icaro/cfg/extraAcq.cfg*, which is daily reloaded by the system.
- Execute the script THacq.py every hour to read air temperature and humidity, and store the values in a file, which is uploaded daily to the acquisition server.
- Execute the script powerReport.py every 3 h to read power voltage and current, and store the values in a log file, which is uploaded daily to the acquisition server.
- If the RMS connection is by the LTE/UMTS router, turn on the router at hours 06:03, 12:03, 18:03, and turn it off after 7 min to provide remote access to RMS.
- If the RMS connection is by LTE/UMTS router, turn on the router at the scheduled time (set by the user in the configuration) and transfer TIR files via FTP protocol to the acquisition server.
- Upload power and system log files and air T-H values via FTP to the acquisition server at 23:55. If the RMS connection is by LTE/UMTS router, the scripts turn on the router at the scheduled time (set by the user in the configuration) and then perform the FTP upload.

### Appendix B.3. System Remote Accessibility by LTE/UMTS Router

When the RMS connection is established by a router, the system’s remote accessibility is allowed by connection windows at hours 06:03, 12:03, 18:03, lasting 7 min. The user can override the router shutdown after 7 min by changing the string “off” to “on” in the file *icaro*/*cfg/router.cfg*. In this way, the router will stay on until the user changes the string to “off”. Times of the connection window can be changed/added by modifying the *time_range_router* parameter (raw 76 of the *icaro.py* file).

- **IRacq.py**—Python script that performs image capture procedures according to the following sequence of operations:

- Switches on the TIR camera.
- Switches on the T-H probe and acquire air temperature and humidity values.
- Sends air temperature and humidity values, target distance, and emissivity to the FLIR TIR camera to perform capture atmospheric corrections.
- Sends Telnet command to the FLIR IR camera to acquire images.
- Copies the IR file from the IR camera to the RasPi micro-SD card.
- Switches off the TIR camera and T-H probe.

- **THacq.py**—Python script that switches on T-H probe, then reads air temperature and humidity values from T-H probe, writes them in the daily .txt file stored in icaro/airTHvalues folder, and finally switches off T-H probe.
- **powerReport.py**—Python script that reads power voltage and current values by querying the INA219 module and writes them in the monthly .log file stored in the icaro/log folder. The script also sends an alert mail if the voltage is lower than 11.5 Volts or the current is higher than 0.8 Ampere. Mail parameters can be set in the function send_mail al raw 95 of the powerReport.py file. Mail will not be sent if the RMS connection is through an LTE/UMTS router.
- **ina219.py** is a Texas Instruments library supporting the INA219 module.
  All the major operations and any errors are reported in the log file icaro/log/icaro.log.

### Appendix B.4. Configuration Settings

The configuration of Icaro is a simple string set in the .txt file *icaro/cfg/icaro.cfg* according to the settings reported in Table A1. The user can change the configuration by editing this file with a text editor or by using the Windows program *updateCFG*.

**Table A1:** Icaro configuration parameters.

ID	Parameter	Type	Variable	Setting
0	TIR Camera IP	IP number	*IP_IRcamera*	
1	*free*			
2	Distance target area	integer	*distance*	
3	*free*	integer		
4	Station Name	string	*station_name*	
5	Emissivity target area	double	*emissivity*	
6	*free*			
7	Server IP	IP number	*ftp_server*	
8	Server Username	string	*ftp_user*	
9	Server User password	string	*ftp_pwd*	
10	Server upload folder	string	*ftp_folder*	
11	*free*			
12	TIR capture times	integer	*acq_mode*	0 = 3 times at night; n=every n hour
13	Extra capture times	integer	*extra_acq*	1 = active; 0 = inactive
14	Connection type	integer	*conn_type*	1 = LAN/WiFi; 2 = LTE/UMTS
15	Air T and H readings	integer	*airTHvalues_acq*	1 = active; 0 = inactive
16	Router IP	IP number	*router_ip*	
17	Router connection hour	string	*router_conn_h*	
18	Router connection min	string	*router_conn_min*	
