# Supplementary material for: Development of a Thermal Infrared Network for Volcanic and Environmental Monitoring: Hardware Design and Data Analysis Software Code
Source: Sensors (Basel). 2025 Jul 2;25(13):4141. doi: 10.3390/s25134141 (PMC12252109; doi:10.3390/s25134141)
Supplement: Supplementary file 1 [file sensors-25-04141-s001.zip › ASIRA User Guide v.1.pdf]

ASIRA for GNU Octave

User Manual

Automated System of InfraRed Analysis

Fabio Sansivero

fabio.sansivero@ingv.it

June 2025

Version 1.0

ISTITUTO NAZIONALE
DI GEOFISICA E VULCANOLOGIA
OSSERVATORIO VESUVIANO

Table of Contents

1 General description of ASIRA 1

1.1 ... Key features and workflow *1*

1.2 ... Outputs *3*

2 Installation of ASIRA and configuration of GNU Octave 7

3 Software usage and functionalities 8

3.1 ... The main GUI *8*

3.2 ... The "Pre-analysis" configuration panel *9*

3.3 ... The "Deseasoning" configuration panel *9*

3.4 ... The "Heat flux" configuration panel *11*

3.5 ... The "Plots" configuration panel *12*

APPENDIX A 14

1 General description of ASIRA

ASIRA (Automated System of InfraRed Analysis) is a software developed by the TIRLab of INGV-Osservatorio Vesuviano to process thermal infrared (TIR) data for monitoring surface temperature changes in volcanic areas or for environmental purpose. Originally created in MATLAB, the revised version presented in this guide has been developed in GNU Octave, a free programming language. This open-source version is available under the Creative Commons Attribution 4.0 License.

The current User Manual refers to ASIRA for Octave version 5.0.

ASIRA includes a user-friendly graphical interface (GUI) to support user in customizing the setting of analysis parameters.

The ASIRA GUI can be executed by launching the `asira_gui.m` file in the OCTAVE IDE.

1.1 Key features and workflow

1. **Data quality assessment:** filters out low-quality TIR frames affected by environmental conditions using a statistical threshold.
2. **Frames co-registration:** corrects pixel misalignments caused by ground movements or instability of the RMS site using an efficient subpixel registration algorithm (co-registration).
3. **Temperature time series extraction:** derives maximum and average temperatures from TIR scene and from user-defined Regions of Interest (ROIs).
4. **Seasonal component removal:** remove seasonal component from the temperature time series using two methods:
    - **BKRp** (Background Removal Procedure) for short time series.
    - **STL** (Seasonal and Trend decomposition using Loess) for almost two years long time series.
5. **Radiative heat flux estimation**: calculates heat flux using the Stefan–Boltzmann law, focusing on high-temperature pixels within ROIs (filtered using statistical threshold).
6. **Thermal anomaly area variations:** tracks changes in the spatial extent of anomalous temperature ROIs by counting high-emittance pixels.

In the Figure 1.1 the main workflow of ASIRA is schematically shown starting from the RAW data (on the top) to the different final products.

A short description of functionalities of the files belonging to the software package is reported in the following Table 1.1.

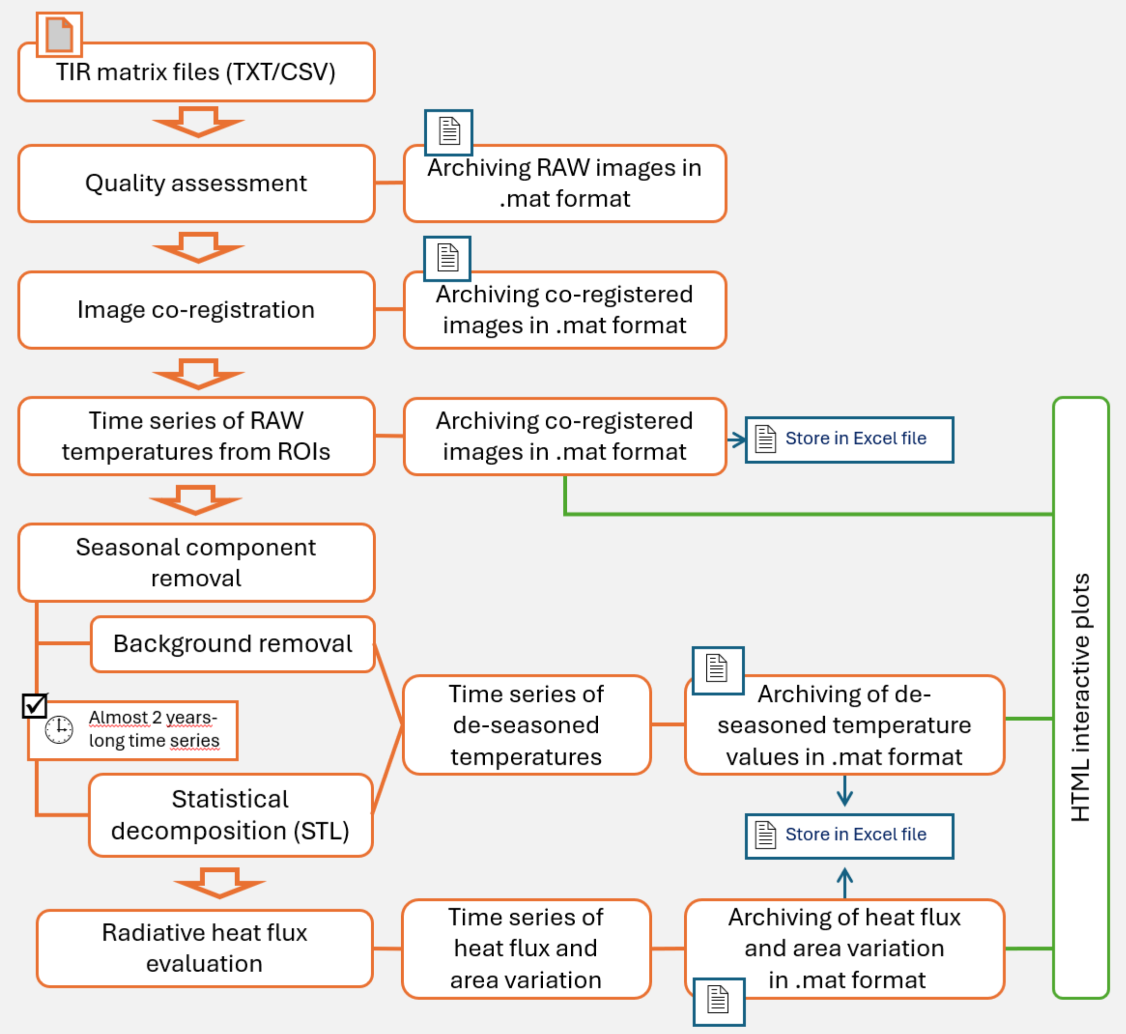

Figure 1.1 Flowchart of main tasks and products of ASIRA

The approach used to design the processing workflow of ASIRA is discussed in the following papers:

Sansivero F., Vilardo G. and Buonocunto C. 2025. Development of a Thermal Infrared Network for Volcanic and Environmental Monitoring: Hardware Design and Data Analysis Software code. Sensors

Sansivero F., Vilardo, G. 2019. Processing thermal infrared imagery time-series from volcano permanent ground-based monitoring network. Latest methodological improvements to characterize surface temperatures behavior of thermal anomaly areas. Remote Sens. 11, 553.

Table 1.1 List of files and functionalities of ASIRA package

File name	*Task performed*
asira_gui.m	Launches main graphical interface (GUI) to start the processing step and save the configuration parameters
packageCheck.m	Checks if required Octave packages are installed
dateInput.m	Manage the input of date in the configuration
archiveData.m	Saves TIR matrix files in .mat archives after performing the quality check
coregFrames.m	Corrects pixel misalignments between TIR frames of the time series (co-registration)
dftregistration.m	Octave library to perform co-registration. © Manuel Guizar
loadRefFile.m	Manages reference file for co-registration procedure
bkgCorrection.m	Removes seasonal component by BKRp
manageBKG.m	Manages the selection and saving of background area
NewImgArea.m	Manages the selection and saving of user-defined regions of interest (ROIs)
createExcelOutputFile.m	Creates the Excel output file, and prepares the sheets in which to insert the data output
fitExcelCols.m	Format the Excel data tables
STLcorrection.m	Removes seasonal component by STLp1 and STLp2
processSTL.m	Manage the R script (deseasonTXT.R) to perform STL correction
RunRcode.m	Calls R statistical package to run R script (deseasonTXT.R) © Weirong Chen
deseasonTXT.R	R script to be launched in R statistical package
HeatFlux.m	Estimates heat flux according to Stefan-Boltzmann law and area variations (percentage of pixels with high emittance)
HFProcessing.m	Performs the procedure to estimate heat flux of different ROIs
webPlot.m	Performs the procedure to generate web plots of TIR data using HTML templates and Jqplot libraries. © Chris Leonello
writeHTMLplotFile.m	Writes plot data to HTML template files

A typical example of processing sequence is:

- ASIRA controls the quality of RAW TIR matrixes and saves them into .mat files split per years (archiveData.m).
- ASIRA corrects pixel misalignments between TIR frames (co-registration) and saves the co-registered frames into .mat files split per years (coregFrames.m).
- ASIRA extracts RAW temperatures from TIR scenes or ROIs and saves results into .mat file split per years and into Excel sheets (bkgCorrection.m).

- ASIRA removes seasonal component by BKRp from TIR scenes or ROIs and save deseasoned temperatures into .mat files split per years and into Excel sheets (bkgCorrection.m).
- ASIRA removes seasonal component by STLp1 and STLp2 from TIR scenes or ROIs and save deseasoned temperatures into .mat files split per years and into Excel sheets (processSTL.m, STLcorrection.m).
- ASIRA estimates heat flux from TIR scenes or ROIs and save values into .mat files split per years and into Excel sheets (HFProcessing.m, HeatFlux.m).
- ASIRA estimates area variations (percentage of pixels with high emittance) from TIR scenes or ROIs and save values into .mat files split per years and into Excel sheets (HeatFlux.m).
- ASIRA generates HTML interactive plots using template files (webPlot.m, writeHTMLplotFile.m).

User can access to the output data by reading the Excel output file which is organized in thematic sheets.

1.2 Outputs

Processed data is saved in MATLAB-compatible .mat files, in Excel spreadsheets (*Output-station_name.xlsx*) and as HTML plots, allowing integration with third-party tools.

Output files are archives of TIR frames (Table 2) or matrixes of temperature values, heat flux values and other parameters extracted by TIR data (Table 3). Detailed description of variables and data type stored in the output files is reported in Appendix A.

Basically, TIR frames can be subdivided in **RAW**, **coregistered** and **deseasoned** ones. RAW frames belong to the initial dataset and the temperature data extracted by these frames is not processed in any way. By evaluating the quality of data, low-quality RAW frames are *excluded*, and high-quality ones are *selected* for the next analysis. Coregistered frames are the *selected* TIR frames whose pixels were aligned to the pixels of a reference frame. Between all coregistered frames, those with the highest maximum temperature values were chosen as representative of the day they were acquired (OneDay frames). The deseasoned frames are those in which the seasonal component has been removed by BKRp and by STLp (if dataset is almost two years long).

Based on this subdivision the different TIR frames are saved as .mat (Matlab archive) or as CSV files as shown in Table 2.

Table 2. List of output files storing processed TIR frames

Frame type	*Description*	*Storage folder*	*File type*	*File name*
RAW all	Initial dataset	Data output	.mat	yyyy-rawIRdata
RAW selected	High quality RAW frames to analyse	Archive	CSV	stn-yyyymmddhhmmss.csv
RAW selected	High quality RAW frames to analyse	Data output	.mat	YYYY-selectedIRdata.mat

RAW excluded	Low quality RAW frames to exclude	Data output	.mat	yyyy-excludedIRdata.mat
Coregistered	RAW frames whose pixels were aligned	Coregistered	CSV	stn-yyyymmddhhmmss.csv
Coregistered	RAW frames whose pixels were aligned	Data output	.mat	yyyy-coregOutputData.mat
OneDay	Coregistered frames rep. of the day they were acquired	Data output	.mat	yyyy-oneDayData.mat
OneDay RAW	Coregistered frames rep. of the day they were acquired	Data output	.mat	AllYearsOneDayRAWData.mat
OneDay Deseasoned	Frames corrected of seasonal component	Data output	.mat	AllYearsSTLcorrData.mat

yyyy = year; mm = month; dd = day; hh = hour; mm = minute; stn = station name

The temperature and heat flux values extracted from the processed TIR frames in the different steps of ASIRA, are saved in .mat files as shown in Table 3.

Finally, interactive HTML plots are generated using jqPlot libraries for easy visualization in any web browser. The HTML plots show the following data extracted from TIR scene and ROIs:

- maximum RAW temperatures
- maximum deseasoned temperature
- heat flux values
- area variations

The HTML plots are saved in the user-defined **Plots output folder.**

Table 3. List of files with processed TIR temperature and heat flux values, saved in Data output folder

Frame type	*Data type*	*File type*	*File name*
Coregistered selected	Tmax, Taver of selected coregistered frames, spit per year	.mat	yyyy-BkgCorrData.mat
Coregistered selected<br>BKRp deseasoned	Tmax, Taver of all selected coregistered frames<br>Tmax, Taver of all BKRp deseasoned frames	.mat	AllYearsCorrData.mat
RAW OneDay<br>BKRp deseasoned OneDay	Tmax, Taver of all RAW selected frames rep. of the day they were acquired<br>Tmax, Taver of BKRp deseasoned frames rep. of the day they were acquired	.mat	AllYearsOneDayCorrData .mat

2 Installation of ASIRA and configuration of GNU Octave

ASIRA code is written for the free programming package GNU Octave which is downloadable at www.octave,org. You must install GNU Octave and then launch the Octave GUI and follow the next simple configuration procedure.

1. Unzip the content of the file Asira.zip. in the folder you prefer.
2. Open Octave and select the folder Asira/IAT_v0.9.3. This folder contains the Image Alignment Toolbox (IAT)[1]. You must permanently install it by executing the following line in the Octave Command Window: `iat_setup('forever')`
3. Install Octave additional packages by executing the following lines in the Octave Command Window:
   - `pkg install -forge io`
   - `pkg install -forge windows`
   - `pkg install -forge parallel`
   - `pkg install -forge image`
   - `pkg install -forge statistics`

   If you encountered problems in downloading packages from forge repositories you can select the folder Asira/OCTAVE packages in the Octave File Browser and then install the needed packages by executing the following line:

   `pkg install` *`package_name`*
4. Download and install the free R statistical software that you can download at https://www.r-project.org/. Additional R libraries must be installed: launch RGui and open the menu '*Packages>Load packages*', then select the following packages: foreign, hms, pkgconfig, R6, Rcpp, readr, rlang. The *RunRcode.m*[2] function calls in Octave the statistical software R to run R script (.r file) used to perform STL seasonal correction.

ASIRA also needs the following folders to manage the input/output data:

Data input folder = folder containing the IR files (TXT or CSV).

Data output folder = folder containing the output files generated by ASIRA.

Archive folder = folder to archive IR files used for analysis.

Coregistered folder = folder to save IR co-registered files.

Plots output folder = folder containing the output HTML plots.

The jqPlot libraries to generate HTML Plots of TIR processed data are included in the folder Asira/template.

ASIRA saves the user-defined configuration files in the folder Asira/CFG.

---

1 IAT, G. Evangelidis - https://sites.google.com/site/imagealignment/

2 Copyright (c) 2017, Wei-Rong Chen

3 Software usage and functionalities

3.1 The main GUI

To start ASIRA launch asira_gui.m in Octave Command Window. The main GUI will appear as shown below in Figure 3.1:

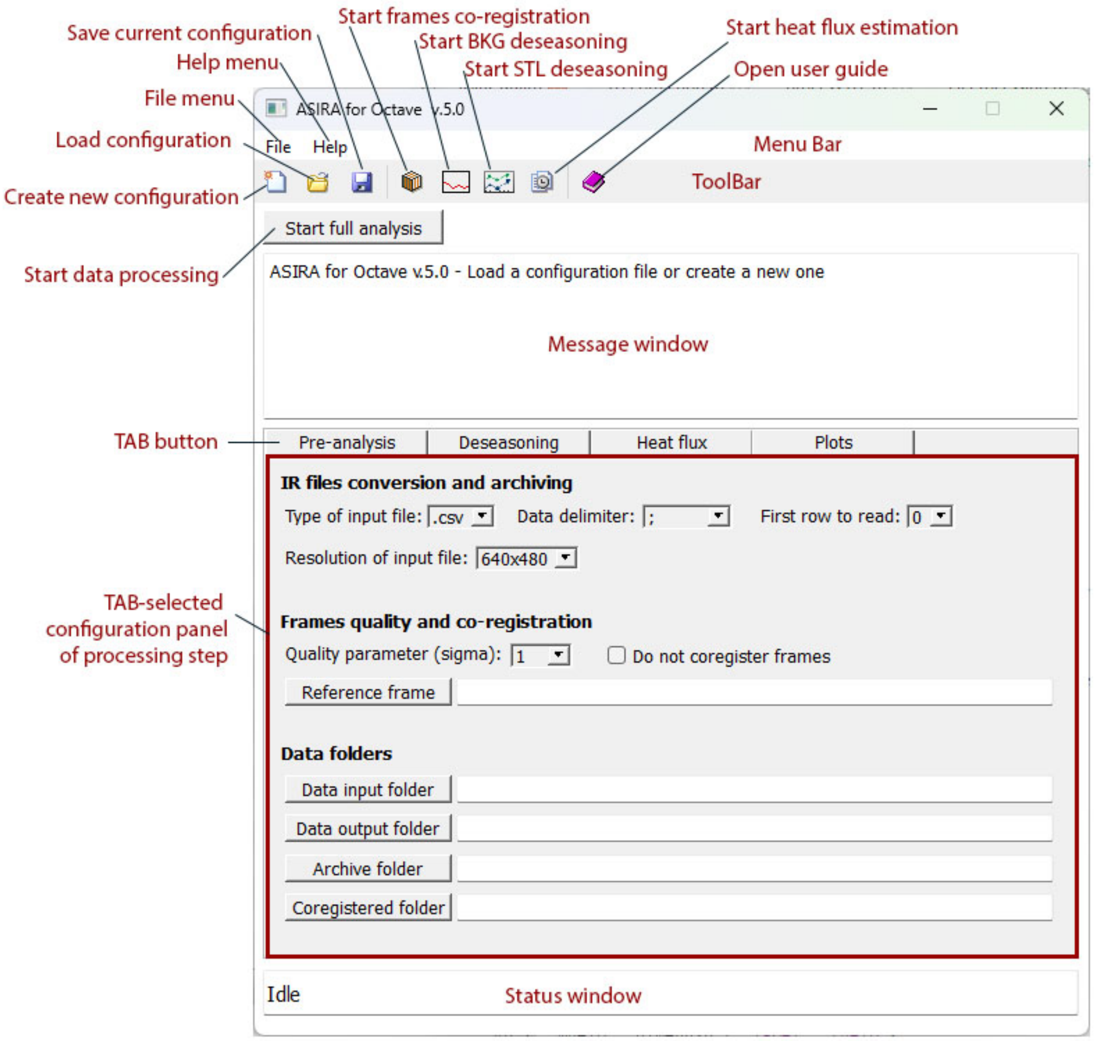

Figure 3.1 Main GUI of ASIRA and short descriptions of its structure and common tools.

The Gui is composed of a Menu Bar, a Tool Bar, a Message Window, four Tab Buttons, four Configuration Panels and a Status Window (Figure 3.1).

TAB buttons allow to manage the different processing steps and configuration parameters in the related configuration panels.

Before processing data, they must be set all configuration parameters in the four configuration panels that are accessed by clicking on the TAB buttons. The configuration can be saved by clicking on the menu "File>Save configuration" or by clicking on the "Save current configuration" button of ToolBar. Previously saved configuration can be loaded by

clicking on the menu “File>Load configuration” or by clicking on the “Load configuration file” button of ToolBar,

To start complete data processing the “Start full analysis” button must be clicked.

User can also process single steps on previously analysed data by clicking on related buttons of the ToolBar.

3.2 The “Pre-analysis” configuration panel

This panel allows to set the main folders and the configuration parameters needed to archive, control the quality and align the pixels of TIR frames (Figure 3.1). You must set all the parameters of this panel before proceeding to the other configuration panels.

Data input folder = Folder containing TXT or CSF temperature matrix files

Data output folder = Folder to which ASIRA will write .mat and Excel output files

Archive folder = Folder to which ASIRA will save the processed .csv TIR files

Coregistrated folder = Folder to which ASIRA will save the .csv TIR files whose pixels were aligned

Almost all the parameters to be set in this panel are intuitive and do not require further explanation except for the “Quality parameter” and “Reference frame”. The “Quality parameter” is used for quality control of TIR frames, higher is the value higher is the quality of selected TIR frames and higher are the number of TIR frames excluded to further analysis. The scientific approach used to evaluate this parameter is discussed in the papers *Sansivero et al., 2025* and *Sansivero F. and Vilardo G., 2019*. The selection of a TIR file as “Reference frame” is needed to the pixel-to-pixel matching between TIR frames across the entire time series (simply called “coregistration”). The “Reference frame” is also necessary to perform the selection of regions of interest (ROIs) and background area.

3.3 The “Deseasoning” configuration panel

This panel is used to configure the parameters required for seasonal component removal.

As discussed in *Sansivero et al, 2025* two different methods are used to remove seasonal component, depending on the length of the time series: BKRp and STLp1&2. The STL method is not applied to time series shorter than two years.

Both methods require the definition of a background area (BKG), which can be selected by clicking the “New” button in the “Background Area Setting” section (Figure 3.2). A dialog box will appear prompting you to enter a name for the new background area. Once confirmed, a TIR image will be displayed. You can define the area by left-clicking to draw a polygon and right-clicking to close it (Figure 3.3). The selected background area can be viewed by clicking the “Show” button in the same section.

Note: You must first select a “Reference Frame” file in the “Pre-analysis Configuration Panel” before performing any of these operations. Otherwise, an error message will appear.

By default, seasonal component removal is applied to the entire TIR scene. However, it can also be performed on user-defined Regions of Interest (ROIs). Up to three ROIs can be created by clicking the “New ROI” buttons in the “Additional Areas of Analysis” section, following the

same procedure used for selecting the background area. Existing ROIs can be viewed by clicking the “Show ROI” buttons. To enable seasonal component removal on a specific ROI, simply check the corresponding “ROI x enabled” box.

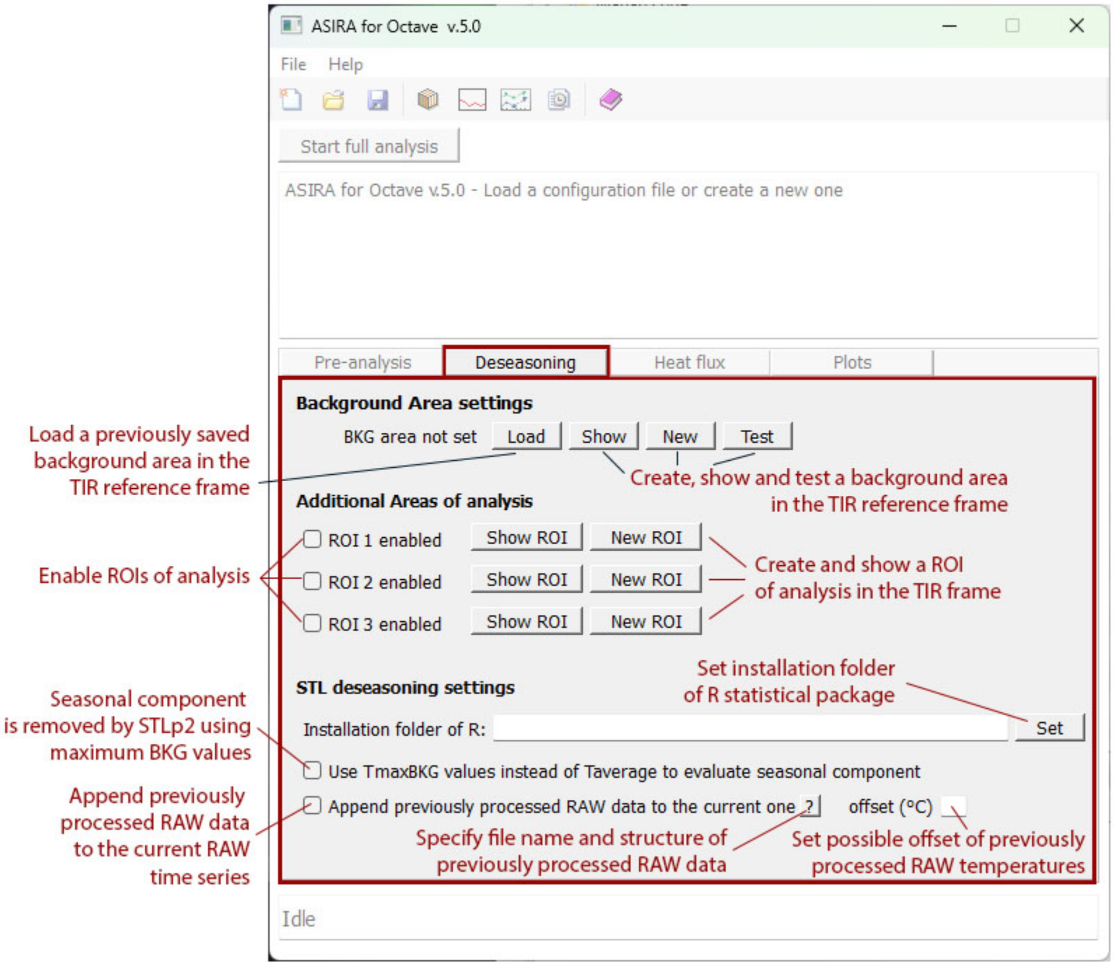

Figure 3.2 The deseasoning configuration panel of ASIRA: short descriptions of settings.

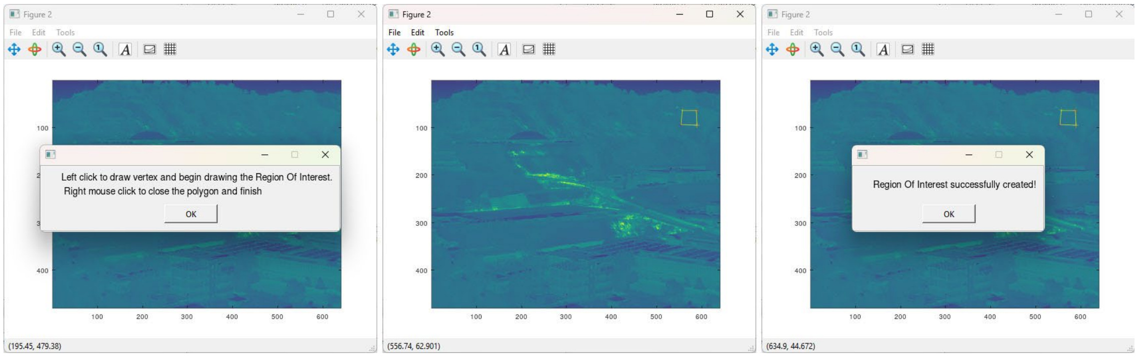

Figure 3.3 The background area selection procedure.

In the "STL Deseasoning Settings" section, you must specify the installation folder of the R statistical package. This is required for ASIRA to perform STL-based seasonal component removal on the TIR temperature values.

The panel also allows you to choose whether to use the average or maximum background temperature for deseasoning when using the STLp2 method. Switching to the maximum value may be beneficial when analyzing areas with thermal anomalies that do not exhibit very high temperatures.

Finally, if you wish to apply seasonal removal to previously processed data, you can append these RAW temperature data to the current dataset by selecting the corresponding checkbox. To view the expected structure and naming convention of the .mat file to append, click the "?" button next to the checkbox. A popup window will provide the necessary information. User can also define an offset between the temperature values of current dataset and the previously processed one.

3.4 The "Heat flux" configuration panel

This panel allows the user to enable or disable heat flux estimation from the entire TIR scene or from user-defined Regions of Interest (ROIs), and to configure the related parameters (Figure 3.4).

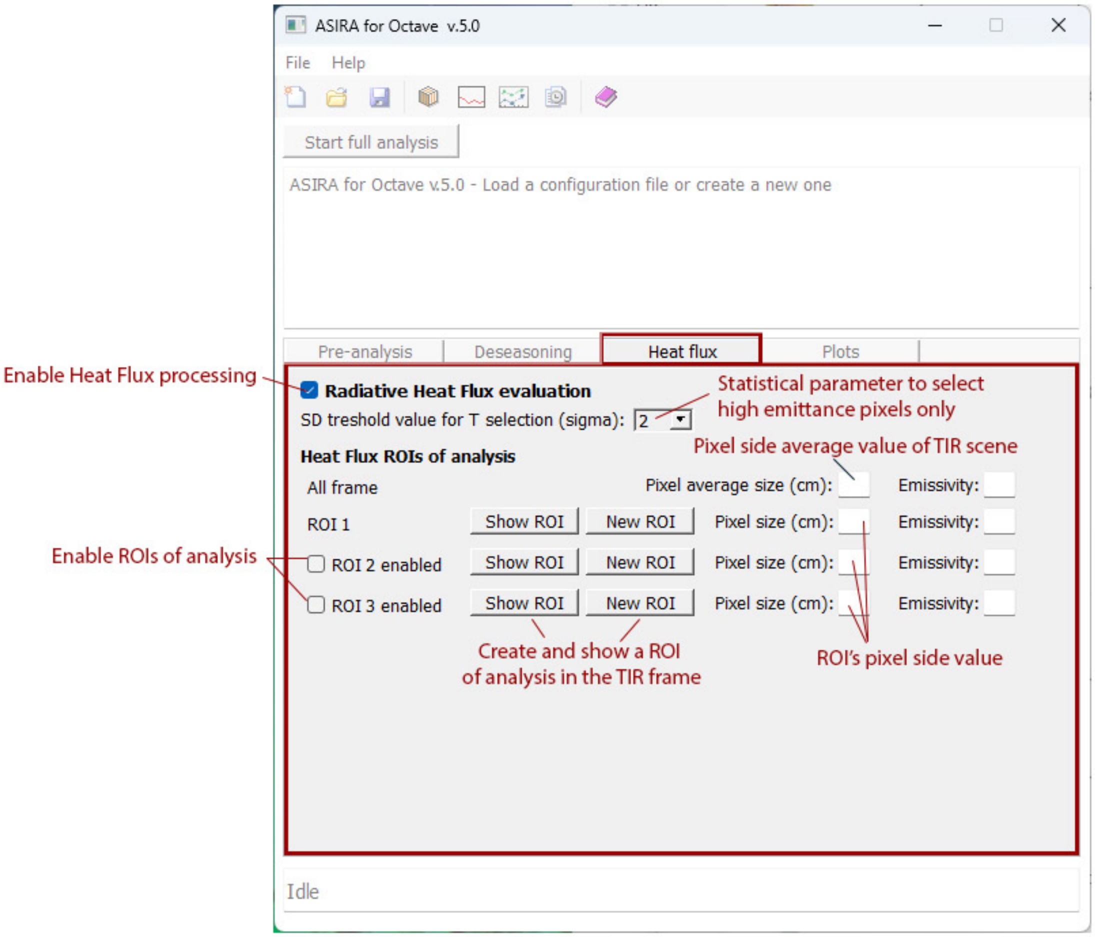

Figure 3.4 The Heat flux configuration panel.

The pixel side length (in cm) and the emissivity values for the selected areas must be specified. Heat flux estimation for Area 2 and Area 3 can be disabled if not needed.

Up to three ROIs can be defined in this panel by clicking the "New ROI" buttons in the "Heat Flux ROIs of Analysis" section, following the same procedure used for selecting the background area (see Paragraph 3.3). Existing ROIs can be viewed by clicking the "Show ROI" buttons.

To ensure accurate heat flux estimation, low-temperature pixels are excluded from the analysis. This is done by setting a threshold value for the standard deviation of temperatures, which the user can easily adjust in this panel.

3.5 The "Plots" configuration panel

As final processing step of ASIRA, this panel allow to enable or disable HTML plots creation and set parameters related to them. HTML plots can be viewed with any Internet Browser and are made with the jqPlot libraries (www.jqplot.com).

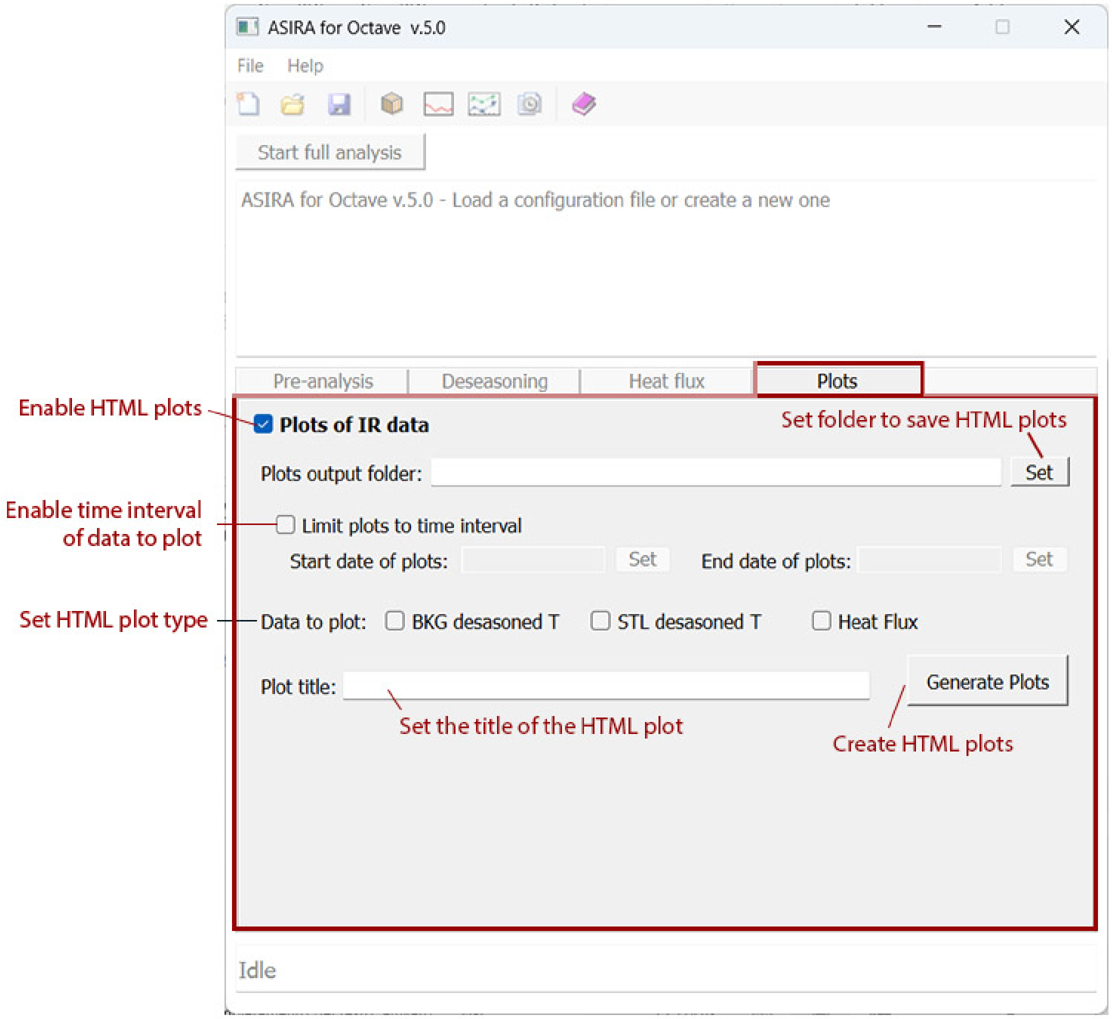

Figure 3.5 The HTML plots configuration panel.

Users can configure the output folder, title, and type of HTML plot to be generated. Additionally, a specific time interval for the plotted data can be defined. If no time interval is specified, the plots will include the entire dataset.
The plots are interactive: hovering with the mouse on a data point displays its temperature value and corresponding date (Figure 3.6a). Users can also zoom in on specific portions of the plot for more detailed view (Figure 3.6b).
By default, HTML plot filenames follow the structure below:

- ***station_name_TrawSTLcorrPlots.html***
  Displays plots of:
  - Maximum RAW temperatures
  - STLp1 deseasoned temperatures (Trend + Residual)
  - STLp1 Trend component
  - STLp1 Seasonal component
    For both the entire TIR scene and the defined ROIs.
- ***station_name_STLcorrPlots.html***
  Displays plots of:
  - Maximum RAW temperatures
  - STLp2 deseasoned temperatures
  - STL Trend (maximum) of the background area
    For both the TIR scene and ROIs.
- ***station_name_HeatFluxPlots.html***
  Displays plots of:
  - Heat flux
  - Percentage of high-emittance pixels (anomaly area variations)
    For both the TIR scene and ROIs.

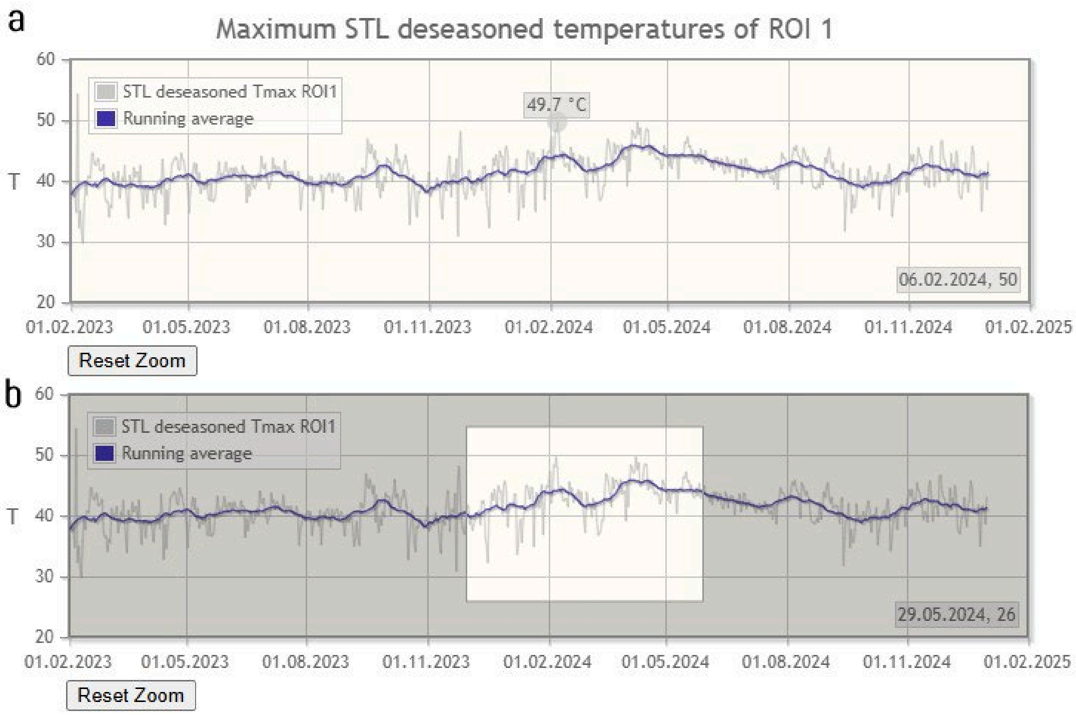

Figure 3.5 Examples of HTML plots. a) hovering with the mouse on a data point displays its temperature valu; b) user can select a portion of the plot to zoom in.

APPENDIX A

Table A1. Output .mat files and related variables and arrays saved inside

Octave .mat file	***Variable/array name***	***n,Type***
YYYY-rawIRdata.mat	RAWFileNames - File names of all RAW TIR frames	1, cell
	RAWframes - 3d array of all RAW TIR frames	3D, double
	RAWSerDate - Serial date of all RAW TIR frames	1, double
	SDrawIR - Standard Deviation of all RAW TIR frames	1, double
YYYY-selectedIRData.mat	SelFileNames - names of the RAW TIR frames which passed the quality control	1, cell
	SelIRframes - 3d array of the RAW TIR frames which passed the quality control	3D, double
	SelSerialDate - Serial date of the RAW TIR frames which passed the quality control	1, double
YYYY-excludedIRData.mat	BadFileNames - File names of the RAW TIR frames which have not passed the quality control	1, cell
	BadIRframes - 3d array of the RAW TIR frames which have not passed the quality control	3D, double
	BadSerialDate - Serial date of the RAW TIR frames which have not passed the quality control	1, double
YYYY-coregOutputData.mat	coRegFileNames - File names of the co-registered frames	1, cell
	coRegFrames - 3d array of the co-registered frames	3D, double
	coRegSerialDate Serial date of the co-registered frames	1, double
YYYY-BkgCorrData.mat	Coords - Coordinates of Tmax pixel inside the TIR frames	2, double
	coRegFileNames - File names of co-registered TIR frames	1, cell
	oneDayFileName - File names of TIR frames that have been chosen as representative of the daily acquisition	1, cell
	oneDayCoords - Coordinates of Tmax pixel inside the TIR frames representative of the daily acquisition	2, double
	Tvalues - Temperatures array of the selected TIR frames*	22, double
	oneDayTvalues - Temperatures array of the TIR frames representative of the daily acquisition*	22, double
YYYY-oneDayData.mat	oneDayCoRegScenes - 3d array of the co-registered frames representative of the daily acquisition	3D, double
	oneDayFileName- File names of TIR frames that have been chosen as representative of the daily acquisition	1, cell
	oneDaySerialDate - Serial date of TIR frames that have been chosen as representative of the daily acquisition	1, double
AllYearsOneDayRawData.mat	totOneDayCoRegScenes - 3d array of the co-registered TIR frames of all the years in the dataset	3D, double
	totOneDayFileName - File names of selected TIR frames of all the years in the dataset	1, cell
	totOneDaySerialDate - Serial date of the co-registered TIR frames of all the years in the dataset	1, double
AllYearsCorrData.mat	totCoords - Coordinates of Tmax pixel inside the TIR co-registered frames of all the years in the dataset	1, double
	totalCoregFileNames - File names of co-registered TIR frames of all the years in the dataset	1, cell

	totTvalues - temperatures array of co-registered TIR frames of all the years in the dataset*	22, double
AllYearsOneDayCorrData.mat	totOneDayCoords - Coordinates of Tmax pixel inside the TIR co-registered frames representative of the daily acquisition of all the years in the dataset	2, double
	totOneDayFileName- File names of selected TIR frames of all the years in the dataset	1, double
	totOneDayTvalues - Temperatures array of the TIR co-registered frames representative of the daily acquisition of all the years in the dataset *	22, double
	totOneDayResampTvalues – Resampled temperatures array of the TIR co-registered frames representative of the daily acquisition of all the years in the dataset *	22, double
AllYearsSTLcorrData.mat	oneDaySerialDateResamp - Serial date of de-seasoned TIR frames of all the years in the dataset	1, double
	oneDaySTLscenes - 3d array of STL de-seasoned TIR frames of all the years in the dataset	3D, double
AllYearsSTLcorrValues.mat	allYearsSTLcorrTvalues - T values extracted by STLp1 method*	14, double
	TmaxRAWSTL - T values extracted by STLp2 method*	13, double
HeatFluxData.mat	HeatFluxData - Heat Flux and area variation values*	13, double
IRDataStats.mat	RAWdataLenght	2, double
	SelDataLenght	2, double
	TotSDrawIR	1, double

YYYY = four digits year. Data stored in .mat files beginning with year digits are related to a specific year

** see Table A2*

Table A2. Arrays and related variables saved in .mat files

Arrays (in red) and related variables				
	**Tvalues totTvalues totOneDayTvalues totOneDayResampTvalues**	**allYearsSTLcorrTvalues**	**TmaxRAWSTL**	**HeatFluxData**
1	SerialDate	SerialDate	resampSerialDate	SerialDate
2	TmaxRawScene	TmaxSTLcorr	TrendScene	HFarea1
3	TmaxCorrScene	TaverSTLcorr	ResidueScene	TotPixA1
4	TaverRawScene	TmaxBKG	SeasonalityScene	HFarea2
5	TaverCorrScene	TaverBKG	TrendA1	TotPixA2
6	TminRawScene	TrendBKG	ResidueA1	HFarea3
7	TmaxBKG	ResidueBKG	SeasonalityA1	TotPixA2
8	TaverBKG	SeasonBKG	TrendA2	HFscene
9	TminBKG	TmaxA1STLcorr	ResidueA2	TotPixScene
10	TmaxRawArea1	TaverA1STLcorr	SeasonalityA2	
11	TmaxCorrArea1	TmaxA2STLcorr	TrendA3	
12	TaverRawArea1	TaverA2STLcorr	ResidueA3	
13	TaverCorrArea1	TmaxA3STLcorr	SeasonalityA3	
14	TmaxRawArea2	TaverA3STLcorr		
15	TmaxCorrArea2			
16	TaverRawArea2			
17	TaverCorrArea2			

18	TmaxRawArea3			
19	TmaxCorrArea3			
20	TaverRawArea3			
21	TaverCorrArea3			
22	AcqHour			

Tmax = maximum temperature
Taver = average temperature
Tmin = minimum temperature
BKG = background area
Raw = RAW temperature
SerialDate = date expressed as integer (timestamp)
Corr= temperature de-seasoned by BKGr method
Scene = TIR frame
AreaX/AX = User-defined ROI to extract temperatures
HFarea = User-defined ROI to compute heat flux
STL = temperature corrected by STLd method
Trend = STL trend
Residue = STL remainer
Seasonality = STL seasonality
TotPix = percentage of high emittance pixels inside a ROI or the Scene
AcqHour = acquisition hour of IR frame
