# Supplementary material for: Development of a Thermal Infrared Network for Volcanic and Environmental Monitoring: Hardware Design and Data Analysis Software Code
Source: Sensors (Basel). 2025 Jul 2;25(13):4141. doi: 10.3390/s25134141 (PMC12252109; doi:10.3390/s25134141)
Supplement: Supplementary file 1 [file sensors-25-04141-s001.zip › Asira.zip!/Asira/template/HFPlotTemplate.html]

PLOT TITLE


|  |  |  |
| --- | --- | --- |
|  | | |
| Reset Zoom | Reset Zoom |  |
| Reset Zoom | Reset Zoom |  |
| Reset Zoom | Reset Zoom |  |
| Reset Zoom | Reset Zoom |  |
| Reset Zoom | Reset Zoom |  |
| Reset Zoom | Reset Zoom |  |
