# Supplementary material for: Development of a Thermal Infrared Network for Volcanic and Environmental Monitoring: Hardware Design and Data Analysis Software Code
Source: Sensors (Basel). 2025 Jul 2;25(13):4141. doi: 10.3390/s25134141 (PMC12252109; doi:10.3390/s25134141)
Supplement: Supplementary file 1 [file sensors-25-04141-s001.zip › Asira.zip!/Asira/OCTAVE packages/io-2.6.4.tar.gz!/io-2.6.4/doc/Spreadsheet-IO-in-Octave.html]

README for spreadsheet file r/w access scripts for Octave (> 3.8.x)  
  
Copyright (C) 2009 - 2020 Philip Nienhuis <prnienhuis at users.sf.net>  
  
Permission is granted to copy, distribute and/or modify this document
under the terms of the GNU Free Documentation License, Version 1.3
or any later version published by the Free Software Foundation;
with no Invariant Sections, no Front-Cover Texts, and no Back-Cover Texts.  
  
February 22, 2020

**SPREADSHEET I/O SUPPORT FOR OCTAVE**


***> > > > AN IMPORTANT THING TO KEEP IN MIND < < < <*** The spreadsheet I/O functions merely serve for **data** I/O, or in other words, just transferring data from or to spreadsheet files. More sophisticated stuff like formatting or overhauling worksheet setup is simply out of scope.

***General overview of functions and files*** As of io-2.6.0, the separate functions for .ods and .xls(x) formats have been unified into the xls\* functions. The ods\* functions still live on as wrappers for their xls\* siblings, although they are now formally deprecated. This means that from now on all spreadsheet formats that used to be divided over the ods\* and xls\* functions can be read / written with either ods\* or xls\* functions. In the overview below the ods\* siblings are indicated between parentheses after their xls\* counterparts. This move was badly needed to be able to cut down on maintenance, as the various ods\* functions did largely the same as their xls\* siblings (think of input validation, preprocessing, input argument handling, file handling, etc.). The choice for keeping xls\* functions and deprecating the ods\* functions is a logical consequence of Octave striving to be Matlab-compatible: Matlab doesn't have ods\* functions, only xls\* functions.

**doc/Spreadsheet-IO-in-Octave.html** This file.

**xlsread.m (odsread.m)** All-in-one functions for reading data from one specific worksheet in a spreadsheet file. This script has Matlab-compatible functionality.

**xlswrite.m (odswrite.m)** All-in-one function for writing data to one specific worksheet in a spreadsheet file. This script has Matlab-compatible functionality.

**xlsfinfo.m (odsfinfo.m)** All-in-one function for exploring basic properties of a spreadsheet file. This script has Matlab-compatible functionality.

**xlsopen.m (odsopen.m)** Function for "opening" (= providing a handle to) a spreadsheet file ("workbook"). This function sorts out which interface to use for .xls(x), .ods, .gnumeric or other file format access (i.e., COM; Java & Apache POI; jOpendocument; JexcelAPI; OpenXLS; etc.), but it's choice can be overridden.

**xls2oct.m (ods2oct.m)** Function for reading data from a specific worksheet pointed to in a struct created by xlsopen.m. xls2oct can be called multiple times consecutively using the same pointer struct, each time allowing to read data from different ranges and/or worksheets. Data are returned in the form of a 2D heterogeneous cell array that can be parsed by parsecell.m. xls2oct is a mere wrapper for (hidden, "private") interface-dependent scripts that do the actual low-level reading.

**oct2xls.m (oct2ods.m)** Function for writing data to a specific worksheet pointed to in a struct created by xlsopen.m. octxls can be called multiple times consecutively using the same pointer struct, each time allowing to write data to different ranges and/or worksheets. oct2xls is a mere wrapper for (hidden, "private") interface-dependent scripts that do the actual low-level writing.

**xlsclose.m (odsclose.m)** Function for closing (the handle to) a spreadsheet file. When data have been written to the workbook in memory, xlsclose will write the workbook to disk. Otherwise, the file pointer is simply closed and possibly used interfaces for spreadsheet access (LibreOffice, COM/ActiveX/Excel.exe) will be shut down properly.

**parsecell.m** Function for separating the data in raw arrays returned by xls2oct, into numerical/logical and text (cell) arrays.

**chk\_spreadsheet\_support.m** Internal function for (1) checking, (2) setting up, (3) debugging spreadsheet support. While not specifically meant for direct invocation from the Octave prompt (it is more useful during initialization of Octave itself) it can be very helpful when hunting down issues with spreadsheet support in Octave

**calccelladdress.m** Support function called by the script functions; calculates spreadsheet type row/column address based on 1-based row/column numbers.

**test\_spsh.m, io\_testscript.m** Undocumented scripts for testing basic features of the spreadsheet scripts. Meant for testers and developers.

***Private utility functions*** Most of the above functions delegate actual file and data handling to "interface"-specific functions that are "private" (= hidden from the user and not directly callable by users).

***Templates*** The io package installation directory contains a "template" subdirectory. This contains empty spreadsheet "frameworks" that are only used as templates for writing data to new spreadsheet files. I do not think they can be read as-is by spreadsheet programs, so don't try that.

**SUPPORT SOFTWARE**

**For the native Octave interface (OCT)** (read/write support for OOXML (Excel 2007+), ODS 1.2 (LibreOffice/OpenOffice.org Calc), and Gnumeric) **NO** external support software is required!

**For the Java / Apache POI / JExcelAPI / OpenXLS / LibreOffice | OpenOffice.org interfaces (general):** - Octave 3.7.2 or later with Java support compiled in; - Java JRE or JDK > 1.8.0 (hasn't been tested with earlier versions); **.ods file format** *ODF Toolkit / ODFDOM specific:* - class .jars **odfdom-java-<version>-incubating.jar**, **xml-apis.jar** and **xercesImpl-<version>.jar**. The preferred versions are odfdom-0.8.8 and xercesImpl-2.11.0. Get them here: http://odftoolkit.org/projects/odfdom/pages/Home http://incubator.apache.org/odftoolkit/downloads.html (preferred) Google for xerces-<version> download *jOpendocument specific:* - class .jar **jOpendocument-<version>.jar**. The best version is 1.4.1-rc2. Get it here: http://www.jopendocument.org **.xls / .xlsx / .xlsm file formats** *Apache POI specific:* - Basic support for .xls: class .jars: **poi-3.5-FINAL-<date>.jar** & **poi-ooxml-3.5-<date>.jar** (or later versions) in javaclasspath. Get them here: http://poi.apache.org/download.html - OOXML (.xlsx, .xlsm) support with Apache POI: **poi-ooxml-schemas-<version>.jar**, + either **xbean.jar** or **xmlbeans.jar** and **dom4j-1.6.1.jar** in javaclasspath. Get them here: http://poi.apache.org/download.html ("xmlbeans" and "poi-ooxml-schemas") http://sourceforge.net/projects/dom4j/files ("dom4j-<version>") Starting with POI-3.15, another Java class lib is required: **commons-collections4-.jar** Recent releases of Apache POI provide zipped / tar.gz'd archives with all required files inside. *JExcelAPI specific (just .xls):* - class .jar: **jxl.jar** in classpath. Get it here: http://sourceforge.net/projects/jexcelapi/files/ *OpenXLS specific (just .xls):* - class .jar: **OpenXLS.jar** in classpath. Get it here: http://sourceforge.net/projects/openxls/ (Be sure to get version 10), and - class .jar: **gwt-servlet-deps.jar** in classpath. Get it from this archive here: http://www.gwtproject.org/download.html ("Download GWT SDK") These class libs must be referenced with full pathnames in your javaclasspath. When the io package gets loaded, a utility function (\_\_init\_io\_\_.m) invokes an initiaization function that tries to automatically find the Java class libs and adds the ones it found to the javaclasspath; When the io package gets unloaded, these same class libs will be removed from the javaclasspath. On MinGW the required Java class libs had best be put in /<libdir>/java (where <libdir> on MinGW is usually /lib); on Linux system supplied Java class libs usually reside in /usr/share/java. Alternatively, you can put them in your HOME directory in a subdirectory java (mind case!) - on \*nix that would be ~./java, on Windows %USERPROFILE%/java (same level as My Documents). The PKG\_ADD routine, that gets run each time the io package is loaded, expects the class libs there; if they are elsewhere, add them in ./share/octave/<version>/m/startup/octaverc using appropriate javaaddpath statements or a chk\_spreadsheet\_support() call. In addition, you can specify a subdirectory using the environment variable OCTAVE\_IO\_JAVALIBS. Once a particular Java class lib has been added to the javaclasspath, it won't be searched anymore nor reloaded from the next search location. The search order is: 1. Specified by the environment variable OCTAVE\_IO\_JAVALIBS 2. <HOME\_DIR>/java 3. /usr/share/java (\*nix) or /lib/java (MinGW) If you do not want to automatically load the Java class libs, specify a value of "no", "false" or "0" for the OCTAVE\_IO\_JAVALIBS environment variable before starting Octave. **.ods, .xls, .xlsx, .xlsm, .sxc + several other file formats** *UNO specific (invoking OpenOffice.org (or clones) behind the scenes)*: NOTE: EXPERIMENTAL!!- A working OpenOffice.org installation. Be aware that OpenOffice.org/LibreOffice arch type (32-bit or 64-bit) must match Octave's arch type. The utility function chk\_spreadsheet\_support had best be used to add the needed entries to the javaclasspath. The relevant Java class libs are **unoil.jar**, **unoloader.jar**, **jurt.jar**, **juh.jar** and **ridl.jar** (which are scattered around the OOo installation directory), while also the **<OOo>/program/** directory needs to be in the classpath. **For the Excel/COM interface:** - A windows computer with MS-Excel installed; - Octave-forge Windows package, version > 1.2.1. Don't forget to load the package.

**USAGE**

**xlsread** (odsread) and **xlswrite** (odswrite) are mere wrappers for **xlsopen** (odsopen) - **xls2oct** (ods2oct) - **xlsclose** (odsclose) - **parsecell** and **xlsopen** (odsopen) - **oct2xls** (oct2ods) - **xlsclose (odsclose)** sequences, resp. They exist for the sake of Matlab compatibility.  
  
**xlsfinfo** (odsfinfo) can be used for finding out what worksheet names exist in the file.  
  
Invoking **xlsopen**/..../**xlsclose** directly provides for much more flexibility, speed, and robustness than **xlsread** / **xlswrite**. Indeed, using the same file handle (pointer struct) you can mix reading & writing before writing the workbook out to disk using xlsclose.  
And: **xlsopen** / **xlsclose** hide the gory interface details from the user.  
  
When using **xlsopen**....**xlsclose** be sure to keep track of the file handle struct.  
  
A possible scenario:  
  
 **xlh = xlsopen (<spreadsheet\_filename> , [rw], [<requested interface>])**   
 *# Set rw to 1 if you want to write to a workbook immediately.  
# In that case the check for file existence is skipped and  
# -if needed- a new workbook created.  
# If you really want an other interface than auto-selected  
# by xlsopen you can request that. But xlsopen still checks  
# proper support for your choice.*   
  
 *# Read some data*   
 **[ rawarr1, xlh ] = xls2oct (xlh, <SomeWorksheet>, <Range>)**   
 *# Be sure to specify xlh as output argument as xls2oct keeps  
# track of changes and the need to write the workbook to disk   
# in the xlhstruct. And the origin range is conveyed through  
# the xlh pointer struct.*   
  
 *# Separate data into numeric and text data*   
 **[ numarr1, txtarr1, lim1 ] = parsecell (rawarr1)**   
  

# Get more data from another worksheet in the same workbook
  
 **[ rawarr2, xlh ] = xls2oct (xlh, <SomeOtherWorksheet>, <Range>)**   
 **[ numarr2, txtarr2, lim2 ] = parsecell (rawarr2)**   
  
# <... Analysis and preparation of new data in cell array Newdata....>  
  
 *# Add new data to spreadsheet*   
 **xlh = oct2xls (Newdata, xlh, <AnotherWorksheet>, <Range>)**   
  
 *# Close the workbook and write it to disk; then clear the handle*   
 **xlh = xlsclose (xlh)**   
 **clear xlh**   

When not using the COM interface, specify a value of 'POI' for parameter REQINTF when accessing OOXML files in xlsread, xlswrite, xlsopen, xlsfinfo (and be sure the complete Apache POI interface is installed). If you haven't got ActiveX installed (i.e., not having MS-Excel under Windows) specifying 'POI' may not be needed as in such cases Apache POI is the next default interface.

When using JExcelAPI (JXL), after writing into a worksheet you MUST save the file � adding data to the same or another worksheet is no more possible after the first call to oct2xls(). This is a limitation of JExcelAPI.

**SPREADSHEET FORMULA SUPPORT, STRIPPING AND ENCODING** 


When using the COM, POI, JXL, OXS, UNO and OCT interfaces you can:

- (When reading, xls2oct) either read evaluated spreadsheet formula results, or the literal formula text strings;
- (When writing, oct2xls) either enter text strings in the form of spreadsheet formulas in the worksheet as formulas, or enter them as literal text strings.

In short, you can enter spreadsheet formulas and in a later stage read them back, change them and re-enter them in the worksheet.

The behaviour is controlled by an option structure **options** which has some fields ("flags") that can be set to TRUE or FALSE:

- options.**formulas\_as\_text** = 0 (the default) implies enter formulas as formulas and read back formula result

  options.**formulas\_as\_text** =1 (or any positive integer) means enter formulas as text strings and read them back as text strings.

  Be aware that there's no formula evaluator in JExcelAPI (JXL), OpenXLS, Odf Toolkit or jOpendocument. So if you create formulas in your spreadsheet using oct2xls or xlswrite with e.g., 'JXL', do not expect meaningful results when reading those files later on **unless** you first open them in LibreOffice, Excel or Apache POI and write them back to disk.

  While both Apache POI and JExcelAPI feature a formula validator, not all spreadsheet functions present in Excel have been implemented (yet).

  Worse, older Excel versions feature less functions than newer versions. So be wary as this may make for interesting confusion.
- options.**strip\_array** = 1 (the default) instructs Octave to strip the output arrays resulting from reading a spreadsheet from empty outer rows and columns.

  options.**strip\_array** = 0 will return the complete requested output range.
- options.**convert\_utf** = 0 (the default) leave UTF-8 encoded text strings read from a spreadsheet untouched. Usually this works well as the Octave terminal usually knows how to display UTF-8 encoded strings - but note that Octve itself has limited support for **processing** multibyte-character strings. Windows 10 has proper support for UTF-8 in the cmd.exe terminal (used by Octave); but older Windows versions may need a conversion step:

  options.**convert\_utf** = 1 currently invokes conversion functions utf82unicode.m (when reading from spreadsheet) or unicode2utf8 (when writing to spreadsheet). This can be useful when the strings in Octave that are intended to be written to spreadsheet file contain characters outside the range [32:127]; Excel and LibreOffice cannot process spreadsheets containing single characters outside that range.


**MATLAB COMPATIBILITY AND SOME GOTCHAS** 


**xlsread**, **xlswrite** and **xlsfinfo** are for the most part Matlab-compatible. Some small differences are mentioned below. When using the Java interfaces Octave supplies some formula manipulation support.  
  
 **xlsread**   

Matlab's **xlsread** flags some spreadsheet errors, Octave's just returns blank cells. Individual spreadsheet cells containing erroneous stuff are transferred to Octave as NaNs. But not all errors can be catched. E.g., spreadsheet cells showing #Value# in LibreOffice Calc often contain invalid formulas but may have a 0 (null) value stored in the value fields. It is rarely possible to catch this as there is no run-time formula evaluator (yet) in ODF Toolkit nor jOpenDocument (like there is in Apache POI for Excel). Octave's **xlsread** (and for that matter, **xlsfinfo** as well) returns info about the actual (rather than the requested) cell range where the data came from. Personally I find it very useful to know from what part of a worksheet the data originate so I've put quite some effort in it :-)

Matlab can't, due to Excel automatically trimming returned arrays from empty outer columns and rows. Octave is more clever but the Visual Basic call used for determining the actually used range has some limitations: (1) it relies on cached range values and thus may be out-of-date, and (2) it counts empty formatted cells too. When using ActiveX/COM, if octave's **xlsfinfo**.m returns wrong data ranges it is most often an overestimation.

Matlab's **xlsread** ignores all non-numeric data values outside the smallest rectangle encompassing all numerical values. Octave's **xlsread** doesn't. This means that Matlab ignores all row/column headers, not very user-friendly IMO.

Matlab's **xlsread** returns strings for cells containing date values. This makes for endless if-then-elseif-else-end constructs to catch all expected date formats. Internally date are stored as doubles with an epoch of 31 Dec. 1899. Octave's xlsread and xls2oct just return those numerical data (where 0 = 1/1/1900 � you can easily transfer them into proper Octave date values yourself by adding 693960. Note that only dates after March 1, 1900 make proper sense because Excel erroneously assumes 1900 to be a leap year.

In .ods file dates are stored as text strings rather than numerical values. Octave reads them into datenums with an epoch of Jan. 1, 0000. Note that you can get an interesting confusing with regard to dates when reading .ods files and then writing them to disk s .xls or .xlsx files, or vice versa.
 **xlswrite**   

Octave's **xlswrite** works on systems w/o Excel support, Matlab's doesn't (properly).

When specifying a sheet number larger than the number of existing sheets in an .xls file, Matlab's **xlswrite** adds empty sheets until the new sheet number is created; Octave's **xlswrite** only adds one sheet called "Sheet<number>" where <number> is the specified sheet number.

Even better (IMO) while M's **xlswrite** always creates Sheet1/Sheet2/Sheet3 when creating a new spreadsheet, Octave's **xlswrite** only creates the requested worksheet. (Did you know that you can instruct Excel to create spreadsheets with just one, or any number of, worksheets? Look in Tools | Options, General tab.)

Oh and octave doesn't touch the "active sheet" - but that's not automatically an advantage.

If the specified write range is larger than the actual data array, Matlab's **xlswrite** adds #N/A cells to fill up the lowermost rows and rightmost columns; octave-forge's **xlswrite** doesn't.

When writing datenums to disk, xlswrite (and oct2xls) doesn't morph them into proper Excel date values. The reason is that neither Octave nor Matlab have date types and conversely, Excel only has double, logical or text types and distinguishes dates from double values solely through formatting. And writing formatting isn't implemented for xlswrite.
 **xlsfinfo**   

When invoking Excel/COM interface, Octave's **xlsfinfo** also echoes the type of sheet (worksheet, chart), not just the sheet names. Using Java I haven't found similar functionality (yet). Octave's **xlsfinfo** also shows (and returns) the range of the smallest rectangle encompassing all occupied data ranges in each sheet.

**COMPARISON OF INTERFACES & USAGE**

The **OCT** or **native Octave** interface is completely under control of Octave (-Forge) developers. Currently it only offers support for .ods (relatively slow), .gnumeric (faster) and OOXML (relatively fast). An immense advantage is that no other external software is required. However, all this comes at a price. Parsing xml trees into rectangular arrays is not quite straightforward and the other way round can be a real nightmare. - **OOXML (.xlx, .xlsm) files** are merely g-zipped directory trees containing XML files. But that's the easy part. Internally all these XML files are cross-linked by various XML tags and especially the XML files that contain the data are extremely complicated. Octave reads those XML files using regular expressions; XML gurus will frown here but using regexps is extremely fast. OTOH it is also fragile as the order of XML tags and subnodes may differ from file to file. However, until now we've seen no problems. - **.ods files** which are also (zipped) archives are different yet equally complicated. Unlike OOXML or gnumeric, .ods spreadsheet cells internally have no cell address tags so developers have to set up their own cell address bookkeeping; regular expressions are of little use here. While reading ODS is doable, writing implies checking whether cells already exist explicitly (in table:table-cells) or implicitly (in number-columns-repeated or number-rows-repeated nodes) or not at all yet in which case you'll need to add various types of parent nodes. Inserting new cells (�nodes�) or deleting nodes implies rebuilding possibly large parts of the tree in memory - nothing for the faint-of-heart. This is the reason that I/O to/from .ods with the OCT interface is quite slow. - **Gnumeric files** are the easiest to read and write and like OOXML regular expressions work well and fast. A particular gotcha with gnumeric is that no cached data values are stored in the file. That means that Octave can't read formula results as hey just don't exist in the file; you really need Gnumeric itself for that. The reason is (as far as I could deduce from their support mailing lists) that the Gnumeric developers figured that files might be 30 % larger and they deemed that too much at the time. So, after reading the above you might appreciate that the io package can also invoke pre-baked libraries that simply shield away the gory details. Most if not all open-source ones are Java based and therefore platform-independent. Read on:

**ODF Toolkit** (ODFDOM) is the one that gives the best (but slow) results at present for .ods. ODF Toolkit up til 0.7.5 did little to hide the gory details for the developers. Only with ODFToolkit (odfdom) 0.8.6, 0.8.7 and 0.8.8 things have been simplified for developers. Unfortunately, with odftoolkit-0.6.0-incubating and odftoolkit-0.6.1-incubating (corresponding to odfdom-0.8.9 and 0.8.10) unresolved dependencies ("jenasin") have been introduced that break their functionality for Octave.

The **jOpenDocument** interface for .ods is more promising (and currently the fastest for not too big spreadsheets), as it does shield the XML tree details and presents to developers something which looks like a spreadsheet model. However, unfortunately the developers decided to shield essential methods by making them 'protected' (e.g. the vital getCellType). jOpenDocument does support writing. But OTOH many obvious methods are still lacking and formula support is absent (although announced for future version 1.4). And last (but not least) the jOpenDocument developers state that their development is primarily driven by requests from customers who pay for support. I do sympathize with this business model but for Octave needs this may hamper progress for a while. The last (and recommended) release was 1.4-rc2 from 2014.

**Apache POI** is based on the OpenOffice.org I/O Excel r/w routines. It is more versatile than JExcelAPI (below); while it doesn't support BIFF5 it does support BIFF8 (Excel 97 � 2003) and OOXML (Excel 2007). It is slower than native JXL let alone Excel & COM but it features active formula evaluation, although still not \*all\* Excel functions have been implemented (a daunting task for the POI devs, as it is hard to keep up with MS here). I've made the relevant subfunction (xls2jpoi2oct) fall back to cached formula results (and yield a suitable warning) for non-implemented Excel functions while reading Excel files.

**JExcelAPI** (for .xls) is proven technology but switching between reading and writing is quite involved and memory-hungry when processing large spreadsheets. As the docs state, JExcelAPI is optimized for reading and it does do that well - but still slower than e.g., OpenXLS. The fact that upon a switch from reading to writing the existing spreadsheet on disk is overwritten **in place** by a blank one and that you can only get the contents back wen writing out all of the changes is worrying - and any change after the first write() is lost as a next write() doesn't seem to work, worse yet, you may completely loose the spreadsheet in question. The first is by JExcelAPI design, the second is probably a bug (in octave-forge/Java or JExcelAPI ? I don't know). Adding data to existing spreadsheets does work, but IMO undue user confidence is needed. JExcelAPI supports BIFF5 (only reading) and BIFF8 (Excel 95 and Excel 97-2003, respectively). Upon overwriting, BIFF5 spreadsheets are converted silently to BIFF8. JexcelAPI, unlike ApachePOI, doesn't evaluate functions while reading but instead relies on cached results (i.e. results computed by Excel itself). Depending on Excel settings ("Automatic calculation" ON or OFF) this may or may not yield incorrect (or expected) results.

**OpenXLS** (an open source version of Extentech's commercial Java-xls product) is still a bit experimental. It seems to work faster than JExcelAPI, but it has other issues - i.e., processing of OOXML files is still unreliable. In addition OpenXLS scatters Extentech.tmp files here and there. For .xls (BIFF8) it works OK; in fact it is the fastest Java-based option for not too big .xls (BIFF8) file I/O.

**UNO** (invoking OpenOffice.org (OOo) or LibreOffice (LO) or clones behind the scenes, a la ActiveX) is experimental. It works FAST (i.e., once OOo itself is loaded and initialized which can take some time) and can process much larger spreadsheets than the other Java-based interfaces because the data are not entered in the JVM but in OOo's own memory. A big stumbling block is that odsclose() on a UNO xls or ods struct will kill ALL OpenOffice.org invocations, also those that were not related to Octave! This is due to UNO-Java limitations. In fact this is the reason UNO is still considered experimental The underlying issue is that after Octave started an Libreoffice invocation, Libreoffice must be closed for Octave to be able to exit; otherwise Octave will wait for LO to shut down before it can terminate itself. So Octave must kill LO to be able to terminate. A way out hasn't been found yet and may even not exist.

Using Excel itself (through **COM / ActiveX** on Windows systems) is probably the most robust and versatile and especially FAST option. There's one gotcha: in case of some type of COM errors Excel will keep running invisibly and turn into a hidden "zombie" process; you can only end it through Task Manager. A tiny problem is that one cannot find out easily through COM what file types are supported; xls, wks, wk1, xlsx, etc.; modern Excel versions can read .ods. Another -obvious- limitation is that COM Excel access only works on Windows systems where Excel is installed.

All in all, of the three Java options I'd prefer Apache POI rather than OpenXLS or JexcelAPI. But the latter is indispensable for BIFF5 formats. Once UNO is stable it is to be preferred as it can read ALL file formats supported by OOo (viz. wk1, ods, xlsx, sxc, ...). If you need to process really large spreadsheets, UNO is by far the fastest option (behind COM on Windows systems), but for smaller spreadsheets you'll find that the other interfaces are more efficient. The **OCT** interface (native Octave calls) is by far the fastest for OOXML, the only Octave option for gnumeric, but for ODS it is still slower than COM/ActiveX or UNO. OCT write support is available for OOXML, ODS 1.2 and gnumeric. Some notes on the choice for Java: 1. It saves a LOT of development time to use ready-baked Java classes rather than developing your own routines and thus effectively reinvent the wheel. 2. A BIG advantage is that a Java-based solution is platform-independent ("portable"). 3. The Java classes offer much more options than just reading and writing. Formatting, recalculation options, hiding/merging cell ranges, etc. 4. But Java is known to be not very conservative with resources, especially not when processing XML-based formats. So Java is a compromise between portability and rapid development time versus capacity (and speed). But IMO data sets larger than 5.10^6^ cells should not be kept in spreadsheets anyway. Better use real databases for such data sets.

**A NOTE ON JAVA MEMORY USAGE**

**Java memory pool allocation size** 

The Java virtual machine (JVM), when initialized by octave, reserves one big chunk of your computer's RAM in which all Java classes and methods etc. are to be loaded: the java memory pool. It does this because Java has a very sophisticated "garbage collection" system. This part of memory is where the Java-based XLS/ODS octave routines live and keep their variables etc.

For transferring large pieces of information to and from spreadsheets you might hit the limits of this pool. E.g. to be able to handle I/O of an array of around 500,000 cells a memory pool size of 512 MB is needed.

The memory size can be increased by inserting a file called "java.opts" (without quotes) in the directory ./share/octave//java (where the script file javaclasspath.m is located; try "which javaclasspath" in an Octave terminal to get the proper location), containing just the following lines:

 **-Xms16m -Xmx512m** 


(where 16 = initial size, 512 = maximum size (in this example), m stands for Megabyte. This maximum is system-dependent. E.g., I have a 1 GB setting). For further details consult the Octave manual, "Java Interface", "FAQ", "How can I handle memory limitations?"

After processing a large chunk of spreadsheet information you might notice that octave's memory footprint does not shrink so it looks like Java's memory pool does not shrink back; but rest assured, the memory footprint is the *allocated* (reserved) memory size, not the actual used size. After the JVM has done its garbage collection, only the so-called "working set" of the memory allocation is really in use and that is a trimmed-down part of the memory allocation pool. On Windows systems it often suffices to minimize the octave terminal for a few seconds to get a more reasonable memory footprint.

**TROUBLESHOOTING**

Some hints for troubleshooting spreadsheet support are contained in this thread (for Excel): http://sourceforge.net/mailarchive/forum.php?thread\_name=4C61B649.9090802%40hccnet.nl&forum\_name=octave-dev dated August 10, 2010. Since that time a lot has changed.

As of April 2011 a special purpose setup file has been included in the io package (**chk\_spreadsheet\_support.m**) in which error checking and troubleshooting have been automated; it has evolved over time with core Octave itself. When running it with the second input argument (debug level) set to 3 a lot of useful, hopefully self-explanatory diagnostic output will be printed to screen.

**DEVELOPMENT**

**xlsopen** / **xlsclose** and friends have been written so that adding other interfaces (Perl? ...?) should be very easily accomplished. **xlsopen.m** merely needs two stanzas, **xlsfinfo.m**, **xls2oct.m**, **oct2xls.m** and **getusedrange.m** each need an additional elseif stanza, and **xlsclose.m** needs a small stanza for closing the pointer struct and writing to disk.

The real work lies in creating the relevant ***\_\_<INTF>\_spsh\_open\_\_.m*** & ***\_\_<INTF>\_spsh\_close\_\_.m***, ***\_\_<INTF>\_spsh2oct\_\_.m*** & ***\_\_<INTF>\_oct2spsh\_\_.m***, ***\_\_<INTF>\_spsh\_info\_\_.m***, and ***\_\_<INTF>\_getusedrange\_\_.m*** subfunction scripts in the ./private subdir, but that need not be really hard, depending on the interface support libraries' quality and documentation. The function scripts in the ./private subdir provide for ample examples. Separating the file access functions and the actual reading/writing from/to the workbook in memory has made developer's life (I mean: my time developing this stuff) much easier.

Some other options for development (who?): - Speeding up, especially Java worksheet/cell access. For cracks, not me. Presently each spreadheet cell is read or written individually; maybe there's a way to treat several cells at once. But the problem is undoubtedly how to translate possibly heterogeneous Java array structures into Octave heterogeneous cell arrays. Using ActiveX/COM an entire array is handed as an object from Excel to Octave and parsed in the Octave world; using OCT a worksheet is scanned using a regular expression for each data type in one fell swoop, and spreadsheets usually only have a limited number of data types (just double, text, logical, and some derived types). - Automatic conversion of spreadsheet date/time values into Octave ones and vice versa (adding or subtracting 636960 in case of ActiveX/COM). But then again Excel's dates are 01-01-1900 based ("epoch"; Octave's epoch is 0-0-0000) and buggy (Excel thinks 1900 is a leap year), and I sometimes have to use dates from before 1900. Maybe as an option? The again each spreadsheet interface support SW has its own date representation and epoch which makes Matlab compatibility over all interfaces an elusive goal. - Creating spreadsheet graphs (a significant enterprise to write from scratch).

Enjoy!

Philip Nienhuis, February 22, 2020
