# Supplementary material for: Development of a Thermal Infrared Network for Volcanic and Environmental Monitoring: Hardware Design and Data Analysis Software Code
Source: Sensors (Basel). 2025 Jul 2;25(13):4141. doi: 10.3390/s25134141 (PMC12252109; doi:10.3390/s25134141)
Supplement: Supplementary file 1 [file sensors-25-04141-s001.zip › Asira.zip!/Asira/OCTAVE packages/windows-1.6.3.tar.gz!/windows-1.6.3/doc/windows.html]

Octave Windows - Windows Toolkit for GNU octave


# Octave Windows - Windows Toolkit for GNU octave

Next: Installing and loading   [Contents][Index]

# Introduction

The Windows toolkit provides a COM interface and additional functionality on Windows for GNU Octave

## Table of Contents

- 1 Installing and loading
  - 1.1 Windows install
  - 1.2 Online Direct install
  - 1.3 Off-line install
  - 1.4 Loading
- 2 Basic Usage Overview
  - 2.1 COM objects
    - 2.1.1 Creating a COM object
    - 2.1.2 Using a COM object
    - 2.1.3 Releasing COM object
  - 2.2 Windows Utility functions
- 3 Function Reference
  - 3.1 Windows Utilities
    - 3.1.1 clipboard
    - 3.1.2 grab
    - 3.1.3 win32\_DeleteRegistry
    - 3.1.4 win32\_MessageBox
    - 3.1.5 win32\_ReadRegistry
    - 3.1.6 win32\_RegEnumKey
    - 3.1.7 win32\_RegEnumValue
    - 3.1.8 win32\_WriteRegistry
    - 3.1.9 win32api
    - 3.1.10 winopen
  - 3.2 COM Interface
    - 3.2.1 @octave\_com\_object/delete
    - 3.2.2 @octave\_com\_object/get
    - 3.2.3 @octave\_com\_object/invoke
    - 3.2.4 @octave\_com\_object/methods
    - 3.2.5 @octave\_com\_object/release
    - 3.2.6 @octave\_com\_object/set
    - 3.2.7 actxserver
    - 3.2.8 com\_atexit
    - 3.2.9 com\_delete
    - 3.2.10 com\_get
    - 3.2.11 com\_invoke
    - 3.2.12 com\_release
    - 3.2.13 com\_set
    - 3.2.14 iscom
  - 3.3 Features
    - 3.3.1 windows\_feature
  - 3.4 Examples
    - 3.4.1 mat2xls
- Appendix A GNU General Public License
- Index

---

Next: Basic Usage Overview, Previous: Introduction, Up: Introduction   [Contents][Index]

## 1 Installing and loading

The Windows toolkit must be installed and then loaded to be used.

It can be installed in GNU Octave directly from octave-forge,
or can be installed in an off-line mode via a downloaded tarball.

The toolkit must be then be loaded once per each GNU Octave session in order to use its functionality.

- Windows install
- Online Direct install
- Off-line install
- Loading

### 1.1 Windows install

If running in Windows, the package may already be installed, to check run:

```
pkg list windows
```

Otherwise it can be installed by installing the requirements and then using the online or offline install method.

### 1.2 Online Direct install

With an internet connection available, the Windows package can be installed from
octave-forge using the following command within GNU Octave:

```
pkg install -forge windows
```

The latest released version of the toolkit will be downloaded and installed.

### 1.3 Off-line install

With the Windows toolkit package already downloaded, and in the current directory when running
GNU Octave, the package can be installed using the following command within GNU Octave:

```
pkg install windows-1.6.3.tar.gz
```

### 1.4 Loading

Regardless of the method of installing the Windows toolkit, in order to use its functions,
the toolkit must be loaded using the pkg load command:

```
pkg load windows
```

The toolkit must be loaded on each GNU Octave session.

---

Next: Function Reference, Previous: Installing and loading, Up: Introduction   [Contents][Index]

## 2 Basic Usage Overview

The Windows package must be loaded each time a GNU Octave session is started:

```
pkg load windows
```

The Windows toolkit provides 2 main types of functionality:

COM Interface functions
:   These are functions that allow interfacing to COM objects.

Windows Utilities
:   Functions that provide additional functions for windows

- COM objects
- Windows Utility functions

### 2.1 COM objects

- Creating a COM object
- Using a COM object
- Releasing COM object

#### 2.1.1 Creating a COM object

To create a COM object, you use the actxserver function with the name of the object to create.

```
wshell = actxserver ("WScript.Shell");
```

A octave\_com\_object type will be returned that provides an interface to the functions and properties of the object.

To get a list of properties for the object, use the get function. Assuming we have the wshell object from the previous example:

```
get(wshell)
```

A list of the properties of the object will be displayed.

To get a list of the methods available for the object, use the methods function.

```
methods(wshell)
```

#### 2.1.2 Using a COM object

Once a object is created using the actxserver function, and you know the methods and properties available, call the
properties of methods using the dot notation.

The following example will display the current directory.

```
wshell = actxserver ("WScript.Shell");
wshell.CurrentDirectory
```

Calling functions is performed in the same way.

```
wshell = actxserver ("WScript.Shell");
wshell.Exec("notepad.exe");
```

#### 2.1.3 Releasing COM object

A COM object should be released when it is not going to be used anymore to free any resources it may have allowed.

```
release(wshell);
```

### 2.2 Windows Utility functions

A number of utility functions are available. See the reference for their usage.

---

Next: GNU General Public License, Previous: Basic Usage Overview, Up: Introduction   [Contents][Index]

## 3 Function Reference

The functions currently available in the Windows toolkit are described below:

- Windows Utilities
- COM Interface
- Features
- Examples

---

Next: COM Interface, Up: Function Reference   [Contents][Index]

### 3.1 Windows Utilities

- clipboard
- grab
- win32\_DeleteRegistry
- win32\_MessageBox
- win32\_ReadRegistry
- win32\_RegEnumKey
- win32\_RegEnumValue
- win32\_WriteRegistry
- win32api
- winopen

#### 3.1.1 clipboard

Loadable Function: *clipboard* **('copy',** *data)* ¶

Loadable Function: *txt* **=** *clipboard ('paste')* ¶
:   Insert or get data from the clipboard.

    ’copy’ or ’paste’ is the required operation to perform.
    where ’copy’ will copy data to the clipboard, and paste will paste data from
    the clipboard to a variable.

    data is the data to copy to the clipboard.

    txt is the text from the clipboard or an empty string it it can not convert to text..

    Examples:

    Copy a string to the clipboard:

    ```
    clipboard('copy', 'hello world');
    ```

    Get a string from the clipboard:

    ```
    txt = clipboard('paste');
    ```

#### 3.1.2 grab

Loadable Function: *[x,y] =* **grab** *(axis)* ¶
:   Grab positions of landmarks on the screen.

    x is the x coordinates of the points.

    y is the y coordinates of the points.

    axis (optional) if specified then the first 2 clicks
    must be on the appropriate axes. x and y (or just x
    if only 2 points specified ) will then be normalised.

    for example:

    ```
    x=grab([1 10])
    ```

    the first two clicks should correspond to x=1 and x=10
    subsequent clicks will then be normalized to graph units.

    for example:

    ```
    [x,y]=grab;
    ```

    gives x and y in screen pixel units (upper left = 0,0 )

    Select points by positioning the cursor over the points
    and clicking <SPACE>. ’q’ or <ESC> quits

#### 3.1.3 win32\_DeleteRegistry

Loadable Function: *code =* **win32\_DeleteRegistry** *(key, subkey, valuename)* ¶
:   Delete a value from the Windows registry.

    Example:

    ```
    key='test\\\\temp';
    # create key
    win32_WriteRegistry('HKLM',key,'test_value', 0)
    # delete it
    win32_DeleteRegistry('HKLM',key,'test_value')
    ```

    key must be one of the following strings:

    HKCR
    :   HKEY\_CLASSES\_ROOT

    HKCU
    :   HKEY\_CURRENT\_USER

    HKLM
    :   HKEY\_LOCAL\_MACHINE

    HKU
    :   HKEY\_USERS

    subkey is the subkey to the registry value.

    valuename is the name of the value to delete from the registry.

    code is the success code. Values correspond to the
    codes in the winerror.h header file. The code of 0 is
    success, while other codes indicate failure

#### 3.1.4 win32\_MessageBox

Loadable Function: *rv =* **win32\_MessageBox** *(title, text)* ¶

Loadable Function: *rv =* **win32\_MessageBox** *(title, text, MboxType)* ¶
:   Display a message box using the win32 API.

    title MessageBox title string

    text MessageBox text string

    MBoxType can be an integer or a string.

    For integer values, consult <windows.h>

    The following string values are recognized:

    - `MB_OK`
    - `MB_OKCANCEL`
    - `MB_ABORTRETRYIGNORE`
    - `MB_YESNOCANCEL`
    - `MB_YESNO`
    - `MB_RETRYCANCEL`

    Default is MB\_OK

    Returns a value rv:

    1
    :   User Clicked OK

    2
    :   User Clicked Cancel

    3
    :   User Clicked Abort
        i

    4
    :   User Clicked Retry

    5
    :   User Clicked Ignore

    6
    :   User Clicked Yes

    7
    :   User Clicked No

    10
    :   User Clicked Try Again

    11
    :   User Clicked Continue

#### 3.1.5 win32\_ReadRegistry

Loadable Function: *[ rv, code ] =* **win32\_ReadRegistry** *(key, subkey, value)* ¶
:   Read a value from the Windows registry.

    Example:

    ```
    key='SOFTWARE\\\\Cygnus Solutions\\\\Cygwin\\\\mounts v2';
    win32_ReadRegistry('HKLM',key,'cygdrive prefix')
    ```

    key must be one of the following strings:

    HKCR
    :   HKEY\_CLASSES\_ROOT

    HKCU
    :   HKEY\_CURRENT\_USER

    HKLM
    :   HKEY\_LOCAL\_MACHINE

    HKU
    :   HKEY\_USERS

    rv is an octave string of the returned bytes.
    This is a natural format for REG\_SZ data; however,
    if the registry data was in another format, REG\_DWORD
    then the calling program will need to process them

    code is the success code. Values correspond to the
    codes in the winerror.h header file. The code of 0 is
    success, while other codes indicate failure
    In the case of failure, ’rv’ will be empty

#### 3.1.6 win32\_RegEnumKey

Loadable Function: *[ rv, code ] =* **win32\_RegEnumKey** *(key, subkey)* ¶
:   Read the keys of a given subkey from the Windows registry.

    Example:

    ```
    key='SOFTWARE\\\\Cygnus Solutions\\\\Cygwin\\\\mounts v2';
    win32_RegEnumKey('HKLM',key)
    ```

    key must be one of the following strings:

    HKCR
    :   HKEY\_CLASSES\_ROOT

    HKCU
    :   HKEY\_CURRENT\_USER

    HKLM
    :   HKEY\_LOCAL\_MACHINE

    HKU
    :   HKEY\_USERS

    rv is an array of value strings for the name of keys
    for a given key and subkey.

    code is the success code. Values correspond to the
    codes in the winerror.h header file. The code of 0 is
    success, while other codes indicate failure
    In the case of failure, ’rv’ will be empty

    **See also:** winqueryreg.

#### 3.1.7 win32\_RegEnumValue

Loadable Function: *[ rv, code ] =* **win32\_RegEnumValue** *(key, subkey)* ¶
:   Read value names from from the Windows registry.

    Example:

    ```
    key='SOFTWARE\\\\Cygnus Solutions\\\\Cygwin\\\\mounts v2';
    win32_RegEnumValue('HKLM',key)
    ```

    key must be one of the following strings:

    HKCR
    :   HKEY\_CLASSES\_ROOT

    HKCU
    :   HKEY\_CURRENT\_USER

    HKLM
    :   HKEY\_LOCAL\_MACHINE

    HKU
    :   HKEY\_USERS

    rv is an array of value strings for the name of values
    for a given key and subkey.

    code is the success code. Values correspond to the
    codes in the winerror.h header file. The code of 0 is
    success, while other codes indicate failure
    In the case of failure, ’rv’ will be empty

    **See also:** winqueryreg.

#### 3.1.8 win32\_WriteRegistry

Loadable Function: *code =* **win32\_WriteRegistry** *(key, subkey, valuename, value)* ¶
:   Write a value to the Windows registry.

    Example:

    ```
    key='test\\\\temp';
    win32_WriteRegistry('HKLM',key,'test_value', 0)
    ```

    key must be one of the following strings:

    HKCR
    :   HKEY\_CLASSES\_ROOT

    HKCU
    :   HKEY\_CURRENT\_USER

    HKLM
    :   HKEY\_LOCAL\_MACHINE

    HKU
    :   HKEY\_USERS

    subkey is the subkey to the registry value.

    valuename is the name of the value to write to the registry.

    value is the value to write. It must be a a string or an integer value.

    code is the success code. Values correspond to the
    codes in the winerror.h header file. The code of 0 is
    success, while other codes indicate failure

#### 3.1.9 win32api

Loadable Function: *rv =* **win32\_MessageBox** *(title, text)* ¶

Loadable Function: *rv =* **win32\_MessageBox** *(title, text, MboxType)* ¶
:   Display a message box using the win32 API.

    title MessageBox title string

    text MessageBox text string

    MBoxType can be an integer or a string.

    For integer values, consult <windows.h>

    The following string values are recognized:

    - `MB_OK`
    - `MB_OKCANCEL`
    - `MB_ABORTRETRYIGNORE`
    - `MB_YESNOCANCEL`
    - `MB_YESNO`
    - `MB_RETRYCANCEL`

    Default is MB\_OK

    Returns a value rv:

    1
    :   User Clicked OK

    2
    :   User Clicked Cancel

    3
    :   User Clicked Abort
        i

    4
    :   User Clicked Retry

    5
    :   User Clicked Ignore

    6
    :   User Clicked Yes

    7
    :   User Clicked No

    10
    :   User Clicked Try Again

    11
    :   User Clicked Continue

#### 3.1.10 winopen

Loadable Function: *winopen* **(name)** ¶
:   Open the file or directory name in the windows registered
    application for the file, using shell open command.

    Examples:

    Open file document.docx in the docx viewer:

    ```
    winopen ("document.docx");
    ```

    Open the current directory in explorer:

    ```
    winopen (pwd);
    ```

---

Next: Features, Previous: Windows Utilities, Up: Function Reference   [Contents][Index]

### 3.2 COM Interface

- @octave\_com\_object/delete
- @octave\_com\_object/get
- @octave\_com\_object/invoke
- @octave\_com\_object/methods
- @octave\_com\_object/release
- @octave\_com\_object/set
- actxserver
- com\_atexit
- com\_delete
- com\_get
- com\_invoke
- com\_release
- com\_set
- iscom

#### 3.2.1 @octave\_com\_object/delete

: **delete** *(obj)* ¶
:   A delete override for octave\_com\_object objects.

    Release interfaces from COM object obj and then delete the COM server.

    **See also:** com\_delete delete.

#### 3.2.2 @octave\_com\_object/get

: *S =* **get** *(obj)* ¶

: *S =* **get** *(obj, propertynames)* ¶
:   A get override for octave\_com\_object objects.

    When specifying just obj, the function will return a list of property names in S.
    When also providing propertynames, the function return the values of the properties.

    **See also:** com\_get, get.

#### 3.2.3 @octave\_com\_object/invoke

: **invoke** *(obj)* ¶

: *S =* **invoke** *(obj, methodname)* ¶

: *S =* **invoke** *(obj, methodname, arg1, …, argN)* ¶
:   Invoke a method on a COM object.

    When called with just the single obj, invoke displays the methods available to the object.
    When called with methodname, invoke will invoke the method with optional args and return
    the result in S.

    **See also:** com\_invoke, methods.

#### 3.2.4 @octave\_com\_object/methods

: **methods** *(obj)* ¶

: *mtds =* **methods** *(obj)* ¶
:   List the names of the public methods for the object octave\_com\_object
    obj.

    When called with no output arguments, `methods` prints the list of
    method names to the screen. Otherwise, the output argument mtds
    contains the list in a cell array of strings.

    **See also:** methods.

#### 3.2.5 @octave\_com\_object/release

: **release** *(obj)* ¶
:   Release the COM object obj and all of its resources.

    **See also:** com\_release, delete.

#### 3.2.6 @octave\_com\_object/set

: *S =* **set** *(obj, propname, value)* ¶
:   A set override for octave\_com\_object objects.

    Call set function on COM object obj to set property propname to value value. Returns any result in S.

    **See also:** com\_set.

#### 3.2.7 actxserver

Loadable Function: *h =* **actxserver** *(progid)* ¶
:   Create a COM server using the progid identifier.

    Returns h, a handle to the default interface of the COM server.

    Example:

    ```
    # create a COM server running Microsoft Excel
    app = actxserver ('Excel.Application');
    # free the object
    destroy (app);
    ```

#### 3.2.8 com\_atexit

Loadable Function: **com\_atexit** *()* ¶
:   Close down all GNU Octave managed COM handles.

    Called during pkg unload.

#### 3.2.9 com\_delete

Loadable Function: **com\_delete** *(obj)* ¶
:   Release interfaces from COM object obj and then delete the COM server

#### 3.2.10 com\_get

Loadable Function: *S =* **com\_get** *(obj)* ¶
:   Call get function on COM object obj. Returns any result in S

#### 3.2.11 com\_invoke

Loadable Function: *result =* **com\_invoke** *(obj)* ¶

Loadable Function: *result =* **com\_invoke** *(obj, method )* ¶
:   Call invoke on obj to run a method, or obtain a list of all methods.

    `com_invoke (obj)` returns a list of all methods available for object obj in result.

    `com_invoke (obj, method )` invokes method method for object obj and returns result result.

#### 3.2.12 com\_release

Loadable Function: **com\_release** *(obj)* ¶
:   Release interfaces from COM object obj

#### 3.2.13 com\_set

Loadable Function: *S =* **com\_set** *(obj, propname, value)* ¶
:   Call set function on COM object obj to set property propname to value value. Returns any result in S

#### 3.2.14 iscom

Function File: *tf =* **iscom** *(h)* ¶
:   Determine whether h is a COM object.

    if h is a COM object, returns true, otherwise returns false.

    **See also:** actxserver.

---

Next: Examples, Previous: COM Interface, Up: Function Reference   [Contents][Index]

### 3.3 Features

- windows\_feature

#### 3.3.1 windows\_feature

Loadable Function: **windows\_feature** *(name)* ¶

Loadable Function: **windows\_feature** *(name, value)* ¶
:   Set or get a feature value.

    name - name of feature to get or set.  
    value - value to set for feature.

---

Previous: Features, Up: Function Reference   [Contents][Index]

### 3.4 Examples

- mat2xls

#### 3.4.1 mat2xls

Function File: **mat2xls** *(obj,filename)* ¶
:   Save obj as an Excel sheet into the file filename. The
    object obj must be either a cell matrix or a real matrix, that
    is a 2-dimensional object. All elements of the matrix are converted
    to Excel cells and put into the first worksheet, starting at cell A1.
    Supported types are real values and strings.

    If filename does not contain any directory, the file is saved
    in the current directory.

    This function is intended to demonstrate the use of the COM interface
    within octave. You need Excel installed on your computer to make this
    function work properly.

    Examples:

    ```
       mat2xls (rand (10, 10), 'test1.xls');
       mat2xls ({'This', 'is', 'a', 'string'}, 'test2.xls');
    ```

---

Next: Index, Previous: Function Reference, Up: Introduction   [Contents][Index]

## Appendix A GNU General Public License


Version 3, 29 June 2007

```
Copyright © 2007 Free Software Foundation, Inc. http://fsf.org/

Everyone is permitted to copy and distribute verbatim copies of this
license document, but changing it is not allowed.
```

### Preamble

The GNU General Public License is a free, copyleft license for
software and other kinds of works.

The licenses for most software and other practical works are designed
to take away your freedom to share and change the works. By contrast,
the GNU General Public License is intended to guarantee your freedom
to share and change all versions of a program—to make sure it remains
free software for all its users. We, the Free Software Foundation,
use the GNU General Public License for most of our software; it
applies also to any other work released this way by its authors. You
can apply it to your programs, too.

When we speak of free software, we are referring to freedom, not
price. Our General Public Licenses are designed to make sure that you
have the freedom to distribute copies of free software (and charge for
them if you wish), that you receive source code or can get it if you
want it, that you can change the software or use pieces of it in new
free programs, and that you know you can do these things.

To protect your rights, we need to prevent others from denying you
these rights or asking you to surrender the rights. Therefore, you
have certain responsibilities if you distribute copies of the
software, or if you modify it: responsibilities to respect the freedom
of others.

For example, if you distribute copies of such a program, whether
gratis or for a fee, you must pass on to the recipients the same
freedoms that you received. You must make sure that they, too,
receive or can get the source code. And you must show them these
terms so they know their rights.

Developers that use the GNU GPL protect your rights with two steps:
(1) assert copyright on the software, and (2) offer you this License
giving you legal permission to copy, distribute and/or modify it.

For the developers’ and authors’ protection, the GPL clearly explains
that there is no warranty for this free software. For both users’ and
authors’ sake, the GPL requires that modified versions be marked as
changed, so that their problems will not be attributed erroneously to
authors of previous versions.

Some devices are designed to deny users access to install or run
modified versions of the software inside them, although the
manufacturer can do so. This is fundamentally incompatible with the
aim of protecting users’ freedom to change the software. The
systematic pattern of such abuse occurs in the area of products for
individuals to use, which is precisely where it is most unacceptable.
Therefore, we have designed this version of the GPL to prohibit the
practice for those products. If such problems arise substantially in
other domains, we stand ready to extend this provision to those
domains in future versions of the GPL, as needed to protect the
freedom of users.

Finally, every program is threatened constantly by software patents.
States should not allow patents to restrict development and use of
software on general-purpose computers, but in those that do, we wish
to avoid the special danger that patents applied to a free program
could make it effectively proprietary. To prevent this, the GPL
assures that patents cannot be used to render the program non-free.

The precise terms and conditions for copying, distribution and
modification follow.

### TERMS AND CONDITIONS

0. Definitions.

   “This License” refers to version 3 of the GNU General Public License.

   “Copyright” also means copyright-like laws that apply to other kinds
   of works, such as semiconductor masks.

   “The Program” refers to any copyrightable work licensed under this
   License. Each licensee is addressed as “you”. “Licensees” and
   “recipients” may be individuals or organizations.

   To “modify” a work means to copy from or adapt all or part of the work
   in a fashion requiring copyright permission, other than the making of
   an exact copy. The resulting work is called a “modified version” of
   the earlier work or a work “based on” the earlier work.

   A “covered work” means either the unmodified Program or a work based
   on the Program.

   To “propagate” a work means to do anything with it that, without
   permission, would make you directly or secondarily liable for
   infringement under applicable copyright law, except executing it on a
   computer or modifying a private copy. Propagation includes copying,
   distribution (with or without modification), making available to the
   public, and in some countries other activities as well.

   To “convey” a work means any kind of propagation that enables other
   parties to make or receive copies. Mere interaction with a user
   through a computer network, with no transfer of a copy, is not
   conveying.

   An interactive user interface displays “Appropriate Legal Notices” to
   the extent that it includes a convenient and prominently visible
   feature that (1) displays an appropriate copyright notice, and (2)
   tells the user that there is no warranty for the work (except to the
   extent that warranties are provided), that licensees may convey the
   work under this License, and how to view a copy of this License. If
   the interface presents a list of user commands or options, such as a
   menu, a prominent item in the list meets this criterion.
1. Source Code.

   The “source code” for a work means the preferred form of the work for
   making modifications to it. “Object code” means any non-source form
   of a work.

   A “Standard Interface” means an interface that either is an official
   standard defined by a recognized standards body, or, in the case of
   interfaces specified for a particular programming language, one that
   is widely used among developers working in that language.

   The “System Libraries” of an executable work include anything, other
   than the work as a whole, that (a) is included in the normal form of
   packaging a Major Component, but which is not part of that Major
   Component, and (b) serves only to enable use of the work with that
   Major Component, or to implement a Standard Interface for which an
   implementation is available to the public in source code form. A
   “Major Component”, in this context, means a major essential component
   (kernel, window system, and so on) of the specific operating system
   (if any) on which the executable work runs, or a compiler used to
   produce the work, or an object code interpreter used to run it.

   The “Corresponding Source” for a work in object code form means all
   the source code needed to generate, install, and (for an executable
   work) run the object code and to modify the work, including scripts to
   control those activities. However, it does not include the work’s
   System Libraries, or general-purpose tools or generally available free
   programs which are used unmodified in performing those activities but
   which are not part of the work. For example, Corresponding Source
   includes interface definition files associated with source files for
   the work, and the source code for shared libraries and dynamically
   linked subprograms that the work is specifically designed to require,
   such as by intimate data communication or control flow between those
   subprograms and other parts of the work.

   The Corresponding Source need not include anything that users can
   regenerate automatically from other parts of the Corresponding Source.

   The Corresponding Source for a work in source code form is that same
   work.
2. Basic Permissions.

   All rights granted under this License are granted for the term of
   copyright on the Program, and are irrevocable provided the stated
   conditions are met. This License explicitly affirms your unlimited
   permission to run the unmodified Program. The output from running a
   covered work is covered by this License only if the output, given its
   content, constitutes a covered work. This License acknowledges your
   rights of fair use or other equivalent, as provided by copyright law.

   You may make, run and propagate covered works that you do not convey,
   without conditions so long as your license otherwise remains in force.
   You may convey covered works to others for the sole purpose of having
   them make modifications exclusively for you, or provide you with
   facilities for running those works, provided that you comply with the
   terms of this License in conveying all material for which you do not
   control copyright. Those thus making or running the covered works for
   you must do so exclusively on your behalf, under your direction and
   control, on terms that prohibit them from making any copies of your
   copyrighted material outside their relationship with you.

   Conveying under any other circumstances is permitted solely under the
   conditions stated below. Sublicensing is not allowed; section 10
   makes it unnecessary.
3. Protecting Users’ Legal Rights From Anti-Circumvention Law.

   No covered work shall be deemed part of an effective technological
   measure under any applicable law fulfilling obligations under article
   11 of the WIPO copyright treaty adopted on 20 December 1996, or
   similar laws prohibiting or restricting circumvention of such
   measures.

   When you convey a covered work, you waive any legal power to forbid
   circumvention of technological measures to the extent such
   circumvention is effected by exercising rights under this License with
   respect to the covered work, and you disclaim any intention to limit
   operation or modification of the work as a means of enforcing, against
   the work’s users, your or third parties’ legal rights to forbid
   circumvention of technological measures.
4. Conveying Verbatim Copies.

   You may convey verbatim copies of the Program’s source code as you
   receive it, in any medium, provided that you conspicuously and
   appropriately publish on each copy an appropriate copyright notice;
   keep intact all notices stating that this License and any
   non-permissive terms added in accord with section 7 apply to the code;
   keep intact all notices of the absence of any warranty; and give all
   recipients a copy of this License along with the Program.

   You may charge any price or no price for each copy that you convey,
   and you may offer support or warranty protection for a fee.
5. Conveying Modified Source Versions.

   You may convey a work based on the Program, or the modifications to
   produce it from the Program, in the form of source code under the
   terms of section 4, provided that you also meet all of these
   conditions:

   1. The work must carry prominent notices stating that you modified it,
      and giving a relevant date.
   2. The work must carry prominent notices stating that it is released
      under this License and any conditions added under section 7. This
      requirement modifies the requirement in section 4 to “keep intact all
      notices”.
   3. You must license the entire work, as a whole, under this License to
      anyone who comes into possession of a copy. This License will
      therefore apply, along with any applicable section 7 additional terms,
      to the whole of the work, and all its parts, regardless of how they
      are packaged. This License gives no permission to license the work in
      any other way, but it does not invalidate such permission if you have
      separately received it.
   4. If the work has interactive user interfaces, each must display
      Appropriate Legal Notices; however, if the Program has interactive
      interfaces that do not display Appropriate Legal Notices, your work
      need not make them do so.

   A compilation of a covered work with other separate and independent
   works, which are not by their nature extensions of the covered work,
   and which are not combined with it such as to form a larger program,
   in or on a volume of a storage or distribution medium, is called an
   “aggregate” if the compilation and its resulting copyright are not
   used to limit the access or legal rights of the compilation’s users
   beyond what the individual works permit. Inclusion of a covered work
   in an aggregate does not cause this License to apply to the other
   parts of the aggregate.
6. Conveying Non-Source Forms.

   You may convey a covered work in object code form under the terms of
   sections 4 and 5, provided that you also convey the machine-readable
   Corresponding Source under the terms of this License, in one of these
   ways:

   1. Convey the object code in, or embodied in, a physical product
      (including a physical distribution medium), accompanied by the
      Corresponding Source fixed on a durable physical medium customarily
      used for software interchange.
   2. Convey the object code in, or embodied in, a physical product
      (including a physical distribution medium), accompanied by a written
      offer, valid for at least three years and valid for as long as you
      offer spare parts or customer support for that product model, to give
      anyone who possesses the object code either (1) a copy of the
      Corresponding Source for all the software in the product that is
      covered by this License, on a durable physical medium customarily used
      for software interchange, for a price no more than your reasonable
      cost of physically performing this conveying of source, or (2) access
      to copy the Corresponding Source from a network server at no charge.
   3. Convey individual copies of the object code with a copy of the written
      offer to provide the Corresponding Source. This alternative is
      allowed only occasionally and noncommercially, and only if you
      received the object code with such an offer, in accord with subsection
      6b.
   4. Convey the object code by offering access from a designated place
      (gratis or for a charge), and offer equivalent access to the
      Corresponding Source in the same way through the same place at no
      further charge. You need not require recipients to copy the
      Corresponding Source along with the object code. If the place to copy
      the object code is a network server, the Corresponding Source may be
      on a different server (operated by you or a third party) that supports
      equivalent copying facilities, provided you maintain clear directions
      next to the object code saying where to find the Corresponding Source.
      Regardless of what server hosts the Corresponding Source, you remain
      obligated to ensure that it is available for as long as needed to
      satisfy these requirements.
   5. Convey the object code using peer-to-peer transmission, provided you
      inform other peers where the object code and Corresponding Source of
      the work are being offered to the general public at no charge under
      subsection 6d.

   A separable portion of the object code, whose source code is excluded
   from the Corresponding Source as a System Library, need not be
   included in conveying the object code work.

   A “User Product” is either (1) a “consumer product”, which means any
   tangible personal property which is normally used for personal,
   family, or household purposes, or (2) anything designed or sold for
   incorporation into a dwelling. In determining whether a product is a
   consumer product, doubtful cases shall be resolved in favor of
   coverage. For a particular product received by a particular user,
   “normally used” refers to a typical or common use of that class of
   product, regardless of the status of the particular user or of the way
   in which the particular user actually uses, or expects or is expected
   to use, the product. A product is a consumer product regardless of
   whether the product has substantial commercial, industrial or
   non-consumer uses, unless such uses represent the only significant
   mode of use of the product.

   “Installation Information” for a User Product means any methods,
   procedures, authorization keys, or other information required to
   install and execute modified versions of a covered work in that User
   Product from a modified version of its Corresponding Source. The
   information must suffice to ensure that the continued functioning of
   the modified object code is in no case prevented or interfered with
   solely because modification has been made.

   If you convey an object code work under this section in, or with, or
   specifically for use in, a User Product, and the conveying occurs as
   part of a transaction in which the right of possession and use of the
   User Product is transferred to the recipient in perpetuity or for a
   fixed term (regardless of how the transaction is characterized), the
   Corresponding Source conveyed under this section must be accompanied
   by the Installation Information. But this requirement does not apply
   if neither you nor any third party retains the ability to install
   modified object code on the User Product (for example, the work has
   been installed in ROM).

   The requirement to provide Installation Information does not include a
   requirement to continue to provide support service, warranty, or
   updates for a work that has been modified or installed by the
   recipient, or for the User Product in which it has been modified or
   installed. Access to a network may be denied when the modification
   itself materially and adversely affects the operation of the network
   or violates the rules and protocols for communication across the
   network.

   Corresponding Source conveyed, and Installation Information provided,
   in accord with this section must be in a format that is publicly
   documented (and with an implementation available to the public in
   source code form), and must require no special password or key for
   unpacking, reading or copying.
7. Additional Terms.

   “Additional permissions” are terms that supplement the terms of this
   License by making exceptions from one or more of its conditions.
   Additional permissions that are applicable to the entire Program shall
   be treated as though they were included in this License, to the extent
   that they are valid under applicable law. If additional permissions
   apply only to part of the Program, that part may be used separately
   under those permissions, but the entire Program remains governed by
   this License without regard to the additional permissions.

   When you convey a copy of a covered work, you may at your option
   remove any additional permissions from that copy, or from any part of
   it. (Additional permissions may be written to require their own
   removal in certain cases when you modify the work.) You may place
   additional permissions on material, added by you to a covered work,
   for which you have or can give appropriate copyright permission.

   Notwithstanding any other provision of this License, for material you
   add to a covered work, you may (if authorized by the copyright holders
   of that material) supplement the terms of this License with terms:

   1. Disclaiming warranty or limiting liability differently from the terms
      of sections 15 and 16 of this License; or
   2. Requiring preservation of specified reasonable legal notices or author
      attributions in that material or in the Appropriate Legal Notices
      displayed by works containing it; or
   3. Prohibiting misrepresentation of the origin of that material, or
      requiring that modified versions of such material be marked in
      reasonable ways as different from the original version; or
   4. Limiting the use for publicity purposes of names of licensors or
      authors of the material; or
   5. Declining to grant rights under trademark law for use of some trade
      names, trademarks, or service marks; or
   6. Requiring indemnification of licensors and authors of that material by
      anyone who conveys the material (or modified versions of it) with
      contractual assumptions of liability to the recipient, for any
      liability that these contractual assumptions directly impose on those
      licensors and authors.

   All other non-permissive additional terms are considered “further
   restrictions” within the meaning of section 10. If the Program as you
   received it, or any part of it, contains a notice stating that it is
   governed by this License along with a term that is a further
   restriction, you may remove that term. If a license document contains
   a further restriction but permits relicensing or conveying under this
   License, you may add to a covered work material governed by the terms
   of that license document, provided that the further restriction does
   not survive such relicensing or conveying.

   If you add terms to a covered work in accord with this section, you
   must place, in the relevant source files, a statement of the
   additional terms that apply to those files, or a notice indicating
   where to find the applicable terms.

   Additional terms, permissive or non-permissive, may be stated in the
   form of a separately written license, or stated as exceptions; the
   above requirements apply either way.
8. Termination.

   You may not propagate or modify a covered work except as expressly
   provided under this License. Any attempt otherwise to propagate or
   modify it is void, and will automatically terminate your rights under
   this License (including any patent licenses granted under the third
   paragraph of section 11).

   However, if you cease all violation of this License, then your license
   from a particular copyright holder is reinstated (a) provisionally,
   unless and until the copyright holder explicitly and finally
   terminates your license, and (b) permanently, if the copyright holder
   fails to notify you of the violation by some reasonable means prior to
   60 days after the cessation.

   Moreover, your license from a particular copyright holder is
   reinstated permanently if the copyright holder notifies you of the
   violation by some reasonable means, this is the first time you have
   received notice of violation of this License (for any work) from that
   copyright holder, and you cure the violation prior to 30 days after
   your receipt of the notice.

   Termination of your rights under this section does not terminate the
   licenses of parties who have received copies or rights from you under
   this License. If your rights have been terminated and not permanently
   reinstated, you do not qualify to receive new licenses for the same
   material under section 10.
9. Acceptance Not Required for Having Copies.

   You are not required to accept this License in order to receive or run
   a copy of the Program. Ancillary propagation of a covered work
   occurring solely as a consequence of using peer-to-peer transmission
   to receive a copy likewise does not require acceptance. However,
   nothing other than this License grants you permission to propagate or
   modify any covered work. These actions infringe copyright if you do
   not accept this License. Therefore, by modifying or propagating a
   covered work, you indicate your acceptance of this License to do so.
10. Automatic Licensing of Downstream Recipients.

    Each time you convey a covered work, the recipient automatically
    receives a license from the original licensors, to run, modify and
    propagate that work, subject to this License. You are not responsible
    for enforcing compliance by third parties with this License.

    An “entity transaction” is a transaction transferring control of an
    organization, or substantially all assets of one, or subdividing an
    organization, or merging organizations. If propagation of a covered
    work results from an entity transaction, each party to that
    transaction who receives a copy of the work also receives whatever
    licenses to the work the party’s predecessor in interest had or could
    give under the previous paragraph, plus a right to possession of the
    Corresponding Source of the work from the predecessor in interest, if
    the predecessor has it or can get it with reasonable efforts.

    You may not impose any further restrictions on the exercise of the
    rights granted or affirmed under this License. For example, you may
    not impose a license fee, royalty, or other charge for exercise of
    rights granted under this License, and you may not initiate litigation
    (including a cross-claim or counterclaim in a lawsuit) alleging that
    any patent claim is infringed by making, using, selling, offering for
    sale, or importing the Program or any portion of it.
11. Patents.

    A “contributor” is a copyright holder who authorizes use under this
    License of the Program or a work on which the Program is based. The
    work thus licensed is called the contributor’s “contributor version”.

    A contributor’s “essential patent claims” are all patent claims owned
    or controlled by the contributor, whether already acquired or
    hereafter acquired, that would be infringed by some manner, permitted
    by this License, of making, using, or selling its contributor version,
    but do not include claims that would be infringed only as a
    consequence of further modification of the contributor version. For
    purposes of this definition, “control” includes the right to grant
    patent sublicenses in a manner consistent with the requirements of
    this License.

    Each contributor grants you a non-exclusive, worldwide, royalty-free
    patent license under the contributor’s essential patent claims, to
    make, use, sell, offer for sale, import and otherwise run, modify and
    propagate the contents of its contributor version.

    In the following three paragraphs, a “patent license” is any express
    agreement or commitment, however denominated, not to enforce a patent
    (such as an express permission to practice a patent or covenant not to
    sue for patent infringement). To “grant” such a patent license to a
    party means to make such an agreement or commitment not to enforce a
    patent against the party.

    If you convey a covered work, knowingly relying on a patent license,
    and the Corresponding Source of the work is not available for anyone
    to copy, free of charge and under the terms of this License, through a
    publicly available network server or other readily accessible means,
    then you must either (1) cause the Corresponding Source to be so
    available, or (2) arrange to deprive yourself of the benefit of the
    patent license for this particular work, or (3) arrange, in a manner
    consistent with the requirements of this License, to extend the patent
    license to downstream recipients. “Knowingly relying” means you have
    actual knowledge that, but for the patent license, your conveying the
    covered work in a country, or your recipient’s use of the covered work
    in a country, would infringe one or more identifiable patents in that
    country that you have reason to believe are valid.

    If, pursuant to or in connection with a single transaction or
    arrangement, you convey, or propagate by procuring conveyance of, a
    covered work, and grant a patent license to some of the parties
    receiving the covered work authorizing them to use, propagate, modify
    or convey a specific copy of the covered work, then the patent license
    you grant is automatically extended to all recipients of the covered
    work and works based on it.

    A patent license is “discriminatory” if it does not include within the
    scope of its coverage, prohibits the exercise of, or is conditioned on
    the non-exercise of one or more of the rights that are specifically
    granted under this License. You may not convey a covered work if you
    are a party to an arrangement with a third party that is in the
    business of distributing software, under which you make payment to the
    third party based on the extent of your activity of conveying the
    work, and under which the third party grants, to any of the parties
    who would receive the covered work from you, a discriminatory patent
    license (a) in connection with copies of the covered work conveyed by
    you (or copies made from those copies), or (b) primarily for and in
    connection with specific products or compilations that contain the
    covered work, unless you entered into that arrangement, or that patent
    license was granted, prior to 28 March 2007.

    Nothing in this License shall be construed as excluding or limiting
    any implied license or other defenses to infringement that may
    otherwise be available to you under applicable patent law.
12. No Surrender of Others’ Freedom.

    If conditions are imposed on you (whether by court order, agreement or
    otherwise) that contradict the conditions of this License, they do not
    excuse you from the conditions of this License. If you cannot convey
    a covered work so as to satisfy simultaneously your obligations under
    this License and any other pertinent obligations, then as a
    consequence you may not convey it at all. For example, if you agree
    to terms that obligate you to collect a royalty for further conveying
    from those to whom you convey the Program, the only way you could
    satisfy both those terms and this License would be to refrain entirely
    from conveying the Program.
13. Use with the GNU Affero General Public License.

    Notwithstanding any other provision of this License, you have
    permission to link or combine any covered work with a work licensed
    under version 3 of the GNU Affero General Public License into a single
    combined work, and to convey the resulting work. The terms of this
    License will continue to apply to the part which is the covered work,
    but the special requirements of the GNU Affero General Public License,
    section 13, concerning interaction through a network will apply to the
    combination as such.
14. Revised Versions of this License.

    The Free Software Foundation may publish revised and/or new versions
    of the GNU General Public License from time to time. Such new
    versions will be similar in spirit to the present version, but may
    differ in detail to address new problems or concerns.

    Each version is given a distinguishing version number. If the Program
    specifies that a certain numbered version of the GNU General Public
    License “or any later version” applies to it, you have the option of
    following the terms and conditions either of that numbered version or
    of any later version published by the Free Software Foundation. If
    the Program does not specify a version number of the GNU General
    Public License, you may choose any version ever published by the Free
    Software Foundation.

    If the Program specifies that a proxy can decide which future versions
    of the GNU General Public License can be used, that proxy’s public
    statement of acceptance of a version permanently authorizes you to
    choose that version for the Program.

    Later license versions may give you additional or different
    permissions. However, no additional obligations are imposed on any
    author or copyright holder as a result of your choosing to follow a
    later version.
15. Disclaimer of Warranty.

    THERE IS NO WARRANTY FOR THE PROGRAM, TO THE EXTENT PERMITTED BY
    APPLICABLE LAW. EXCEPT WHEN OTHERWISE STATED IN WRITING THE COPYRIGHT
    HOLDERS AND/OR OTHER PARTIES PROVIDE THE PROGRAM “AS IS” WITHOUT
    WARRANTY OF ANY KIND, EITHER EXPRESSED OR IMPLIED, INCLUDING, BUT NOT
    LIMITED TO, THE IMPLIED WARRANTIES OF MERCHANTABILITY AND FITNESS FOR
    A PARTICULAR PURPOSE. THE ENTIRE RISK AS TO THE QUALITY AND
    PERFORMANCE OF THE PROGRAM IS WITH YOU. SHOULD THE PROGRAM PROVE
    DEFECTIVE, YOU ASSUME THE COST OF ALL NECESSARY SERVICING, REPAIR OR
    CORRECTION.
16. Limitation of Liability.

    IN NO EVENT UNLESS REQUIRED BY APPLICABLE LAW OR AGREED TO IN WRITING
    WILL ANY COPYRIGHT HOLDER, OR ANY OTHER PARTY WHO MODIFIES AND/OR
    CONVEYS THE PROGRAM AS PERMITTED ABOVE, BE LIABLE TO YOU FOR DAMAGES,
    INCLUDING ANY GENERAL, SPECIAL, INCIDENTAL OR CONSEQUENTIAL DAMAGES
    ARISING OUT OF THE USE OR INABILITY TO USE THE PROGRAM (INCLUDING BUT
    NOT LIMITED TO LOSS OF DATA OR DATA BEING RENDERED INACCURATE OR
    LOSSES SUSTAINED BY YOU OR THIRD PARTIES OR A FAILURE OF THE PROGRAM
    TO OPERATE WITH ANY OTHER PROGRAMS), EVEN IF SUCH HOLDER OR OTHER
    PARTY HAS BEEN ADVISED OF THE POSSIBILITY OF SUCH DAMAGES.
17. Interpretation of Sections 15 and 16.

    If the disclaimer of warranty and limitation of liability provided
    above cannot be given local legal effect according to their terms,
    reviewing courts shall apply local law that most closely approximates
    an absolute waiver of all civil liability in connection with the
    Program, unless a warranty or assumption of liability accompanies a
    copy of the Program in return for a fee.

### END OF TERMS AND CONDITIONS

### How to Apply These Terms to Your New Programs

If you develop a new program, and you want it to be of the greatest
possible use to the public, the best way to achieve this is to make it
free software which everyone can redistribute and change under these
terms.

To do so, attach the following notices to the program. It is safest
to attach them to the start of each source file to most effectively
state the exclusion of warranty; and each file should have at least
the “copyright” line and a pointer to where the full notice is found.

```
one line to give the program's name and a brief idea of what it does.  
Copyright (C) year name of author

This program is free software: you can redistribute it and/or modify
it under the terms of the GNU General Public License as published by
the Free Software Foundation, either version 3 of the License, or (at
your option) any later version.

This program is distributed in the hope that it will be useful, but
WITHOUT ANY WARRANTY; without even the implied warranty of
MERCHANTABILITY or FITNESS FOR A PARTICULAR PURPOSE.  See the GNU
General Public License for more details.

You should have received a copy of the GNU General Public License
along with this program.  If not, see http://www.gnu.org/licenses/.
```

Also add information on how to contact you by electronic and paper mail.

If the program does terminal interaction, make it output a short
notice like this when it starts in an interactive mode:

```
program Copyright (C) year name of author 
This program comes with ABSOLUTELY NO WARRANTY; for details type ‘show w’.
This is free software, and you are welcome to redistribute it
under certain conditions; type ‘show c’ for details.
```

The hypothetical commands ‘`show w`’ and ‘`show c`’ should show
the appropriate parts of the General Public License. Of course, your
program’s commands might be different; for a GUI interface, you would
use an “about box”.

You should also get your employer (if you work as a programmer) or school,
if any, to sign a “copyright disclaimer” for the program, if necessary.
For more information on this, and how to apply and follow the GNU GPL, see
http://www.gnu.org/licenses/.

The GNU General Public License does not permit incorporating your
program into proprietary programs. If your program is a subroutine
library, you may consider it more useful to permit linking proprietary
applications with the library. If this is what you want to do, use
the GNU Lesser General Public License instead of this License. But
first, please read http://www.gnu.org/philosophy/why-not-lgpl.html.

---

Previous: GNU General Public License, Up: Introduction   [Contents][Index]

## Index

|  |  |
| --- | --- |
| Jump to: | **A**   **B**   **C**   **D**   **E**   **F**   **G**   **I**   **L**   **M**   **O**   **R**   **S**   **W** |

|  |  |  |  |
| --- | --- | --- | --- |
|  | Index Entry |  | Section |
| --- | | | |
| A |  |  |  |
|  | actxserver: |  | COM Interface |
| --- | | | |
| B |  |  |  |
|  | Basic Usage Overview: |  | Basic Usage Overview |
| --- | | | |
| C |  |  |  |
|  | clipboard: |  | Windows Utilities |
|  | COM Interface: |  | COM Interface |
|  | com\_atexit: |  | COM Interface |
|  | com\_delete: |  | COM Interface |
|  | com\_get: |  | COM Interface |
|  | com\_invoke: |  | COM Interface |
|  | com\_release: |  | COM Interface |
|  | com\_set: |  | COM Interface |
|  | copyright: |  | Copying |
| --- | | | |
| D |  |  |  |
|  | delete: |  | COM Interface |
| --- | | | |
| E |  |  |  |
|  | Examples: |  | Examples |
| --- | | | |
| F |  |  |  |
|  | Features: |  | Features |
|  | Function Reference: |  | Function Reference |
| --- | | | |
| G |  |  |  |
|  | get: |  | COM Interface |
|  | grab: |  | Windows Utilities |
| --- | | | |
| I |  |  |  |
|  | Installing and loading: |  | Installing and loading |
|  | invoke: |  | COM Interface |
|  | iscom: |  | COM Interface |
| --- | | | |
| L |  |  |  |
|  | Loading: |  | Installing and loading |
| --- | | | |
| M |  |  |  |
|  | mat2xls: |  | Examples |
|  | methods: |  | COM Interface |
| --- | | | |
| O |  |  |  |
|  | Off-line install: |  | Installing and loading |
|  | Online install: |  | Installing and loading |
| --- | | | |
| R |  |  |  |
|  | release: |  | COM Interface |
| --- | | | |
| S |  |  |  |
|  | set: |  | COM Interface |
| --- | | | |
| W |  |  |  |
|  | warranty: |  | Copying |
|  | win32api: |  | Windows Utilities |
|  | win32\_DeleteRegistry: |  | Windows Utilities |
|  | win32\_MessageBox: |  | Windows Utilities |
|  | win32\_ReadRegistry: |  | Windows Utilities |
|  | win32\_RegEnumKey: |  | Windows Utilities |
|  | win32\_RegEnumValue: |  | Windows Utilities |
|  | win32\_WriteRegistry: |  | Windows Utilities |
|  | Windows install: |  | Installing and loading |
|  | Windows Utilities: |  | Windows Utilities |
|  | windows\_feature: |  | Features |
|  | winopen: |  | Windows Utilities |
| --- | | | |

|  |  |
| --- | --- |
| Jump to: | **A**   **B**   **C**   **D**   **E**   **F**   **G**   **I**   **L**   **M**   **O**   **R**   **S**   **W** |
