# Supplementary material for: Development of a Thermal Infrared Network for Volcanic and Environmental Monitoring: Hardware Design and Data Analysis Software Code
Source: Sensors (Basel). 2025 Jul 2;25(13):4141. doi: 10.3390/s25134141 (PMC12252109; doi:10.3390/s25134141)
Supplement: Supplementary file 1 [file sensors-25-04141-s001.zip › Asira.zip!/Asira/OCTAVE packages/parallel-4.0.2.tar.gz!/parallel-4.0.2/doc/html/netcellfun.html]

netcellfun (parallel\_doc)


Next: netarrayfun, Previous: network\_set, Up: Cluster execution   [Index]

---


### 3.8 Function netcellfun


Function File: **netcellfun** *(connections, fun, …)*
:   Evaluates function fun in a parallel cluster and collects results.

    This function handles arguments and options equivalently to
    `parcellfun` and returnes equivalent output. Differently, the
    first argument specifies server machines for parallel remote
    execution, see `pconnect` for a description of the
    connections variable. A further difference is that the option
    "ChunksPerProc" is ignored and instead the chunk size can be
    specified directly with an option "ChunkSize".

    This function can only be successfully called at the client machine
    of the parallel cluster. connections can contain all
    connections of the network, a subset of them, or a single connection.
    The local machine (client), if contained in connections, is
    ignored. However, one of the servers can run at the local machine
    under certain conditions (see `pconnect`) and will not be
    ignored in this case, so that the local machine can take part in
    parallel execution with `netcellfun`.

    As a second level of parallelism, fun is executed at each
    server machine (using `parcellfun or pararrayfun`) by default in
    as many local processes in parallel as the server has processor cores
    available. The number of local parallel processes can be configured
    for each server with the "nlocaljobs" option (see
    `network_set`), a value of `0` means that the default value
    will be used, a value of `1` means that execution is not
    parallel within the server (but still parallel over the cluster).

    See `parallel_doc ("limitations")` for possible limitations
    e.g. on how fun can be defined.

    Cluster execution incurs a considerable overhead. A speedup is
    likely if the computation time of fun is long. To speed up
    execution of a large set of arguments with short computation times
    of fun, increase "ChunkSize", possibly use "Vectorize" (see
    `pararrayfun`), and possibly experiment with increasing
    "nlocaljobs" from the default.

    **See also:** netarrayfun, pconnect, pserver, sclose, rfeval, install\_vars.

---

Next: netarrayfun, Previous: network\_set, Up: Cluster execution   [Index]
