# Supplementary material for: Development of a Thermal Infrared Network for Volcanic and Environmental Monitoring: Hardware Design and Data Analysis Software Code
Source: Sensors (Basel). 2025 Jul 2;25(13):4141. doi: 10.3390/s25134141 (PMC12252109; doi:10.3390/s25134141)
Supplement: Supplementary file 1 [file sensors-25-04141-s001.zip › Asira.zip!/Asira/OCTAVE packages/parallel-4.0.2.tar.gz!/parallel-4.0.2/doc/html/Cluster-execution.html]

Cluster execution (parallel\_doc)


Next: Further functions, Previous: Local execution, Up: Top   [Index]

---


## 3 Functions for parallel execution over a cluster of machines

There are high level functions, similar to parcellfun and
pararrayfun, which only require the user to provide a function to
be called and a series of argument sets. Parallel function calls are
then internally distributed over the cluster, and over the local
processors (or processor cores) within the single machines of the
cluster.

Medium and low level functions are also provided. These require the user
to program the scheduling of parallel execution.

Data transfer is possible between the client machine and each server
machine, but also directly between server machines. The latter feature
is exploited e.g. by the install\_vars function.

For limitations, see Limitations.

|  |  |  |
| --- | --- | --- |
| • Security |  | Security considerations. |
| • Authentication |  | Generating authentication keys. |
| • pserver |  | Starting servers. |
| ``` Connection-related functions ``` | | |
| • pconnect |  | Establishing cluster connections. |
| • sclose |  | Closing cluster connections. |
| • network\_get\_info |  | Get network information. |
| • network\_set |  | Set network properties. |
| ``` High level functions ``` | | |
| • netcellfun |  | Function netcellfun. |
| • netarrayfun |  | Function netarrayfun. |
| • install\_vars |  | Distribute Octave variables. |
| ``` Medium level functions ``` | | |
| • rfeval |  | Single remote function evaluation. |
| ``` Low level functions ``` | | |
| • psend |  | Sending Octave variables. |
| • precv |  | Receiving Octave variables. |
| • reval |  | Remote command evaluation. |
| • select\_sockets |  | Call Unix `select` for data connections. |
|  | | |
| • Example |  | Example |

---

Next: Further functions, Previous: Local execution, Up: Top   [Index]
