# Supplementary material for: Development of a Thermal Infrared Network for Volcanic and Environmental Monitoring: Hardware Design and Data Analysis Software Code
Source: Sensors (Basel). 2025 Jul 2;25(13):4141. doi: 10.3390/s25134141 (PMC12252109; doi:10.3390/s25134141)
Supplement: Supplementary file 1 [file sensors-25-04141-s001.zip › Asira.zip!/Asira/OCTAVE packages/parallel-4.0.2.tar.gz!/parallel-4.0.2/doc/html/XREFnetwork_005fset.html]

XREFnetwork\_set (parallel\_doc)


The node you are looking for is at XREFnetwork\_set.
