# Supplementary material for: Development of a Thermal Infrared Network for Volcanic and Environmental Monitoring: Hardware Design and Data Analysis Software Code
Source: Sensors (Basel). 2025 Jul 2;25(13):4141. doi: 10.3390/s25134141 (PMC12252109; doi:10.3390/s25134141)
Supplement: Supplementary file 1 [file sensors-25-04141-s001.zip › Asira.zip!/Asira/OCTAVE packages/parallel-4.0.2.tar.gz!/parallel-4.0.2/doc/html/parcellfun_005fset_005fnproc.html]

parcellfun\_set\_nproc (parallel\_doc)


Previous: pararrayfun, Up: Local execution   [Index]

---


### 2.3 Function parcellfun\_set\_nproc


Loadable Function: *nproc =* **parcellfun\_set\_nproc** *(nproc\_arg)*
:   Set current number of background processes for parcellfun.

    The number of used background processes is determined by the first argument
    to `parcellfun`. If necessary, `parcellfun` will spawn new
    background processes, but it will never remove any of them, even if it
    does not use all of them.
    `parcellfun_set_nproc` can be used to set the number of currently spawned
    processes to nproc\_arg. This may be used to remove all background
    processes between calls to `parcellfun` by specifying an nproc\_arg
    of zero.

    The real number of spawned processes will be limited to the available
    processor cores and is returned.

    **See also:** parcellfun.
