# Supplementary material for: Development of a Thermal Infrared Network for Volcanic and Environmental Monitoring: Hardware Design and Data Analysis Software Code
Source: Sensors (Basel). 2025 Jul 2;25(13):4141. doi: 10.3390/s25134141 (PMC12252109; doi:10.3390/s25134141)
Supplement: Supplementary file 1 [file sensors-25-04141-s001.zip › Asira.zip!/Asira/OCTAVE packages/parallel-4.0.2.tar.gz!/parallel-4.0.2/doc/html/Function-index.html]

Function index (parallel\_doc)


Next: Concept index, Previous: Documentation, Up: Top   [Index]

---


## Index of functions in parallel

|  |  |
| --- | --- |
| Jump to: | **F**   **I**   **N**   **P**   **R**   **S** |

|  |  |  |  |
| --- | --- | --- | --- |
|  | Index Entry |  | Section |
| --- | | | |
| F |  |  |  |
|  | `fload`: |  | fsave fload |
|  | `fsave`: |  | fsave fload |
| --- | | | |
| I |  |  |  |
|  | `install_vars`: |  | install\_vars |
| --- | | | |
| N |  |  |  |
|  | `netarrayfun`: |  | netarrayfun |
|  | `netcellfun`: |  | netcellfun |
|  | `network_get_info`: |  | network\_get\_info |
|  | `network_set`: |  | network\_set |
| --- | | | |
| P |  |  |  |
|  | `parallel_doc`: |  | Documentation |
|  | `parallel_generate_srp_data`: |  | Authentication |
|  | `pararrayfun`: |  | pararrayfun |
|  | `parcellfun`: |  | parcellfun |
|  | `parcellfun_set_nproc`: |  | parcellfun\_set\_nproc |
|  | `pconnect`: |  | pconnect |
|  | `precv`: |  | precv |
|  | `psend`: |  | psend |
|  | `pserver`: |  | pserver |
| --- | | | |
| R |  |  |  |
|  | `reval`: |  | reval |
|  | `rfeval`: |  | rfeval |
| --- | | | |
| S |  |  |  |
|  | `sclose`: |  | sclose |
|  | `select`: |  | select |
|  | `select sockets`: |  | select\_sockets |
| --- | | | |

|  |  |
| --- | --- |
| Jump to: | **F**   **I**   **N**   **P**   **R**   **S** |
