# Supplementary material for: Development of a Thermal Infrared Network for Volcanic and Environmental Monitoring: Hardware Design and Data Analysis Software Code
Source: Sensors (Basel). 2025 Jul 2;25(13):4141. doi: 10.3390/s25134141 (PMC12252109; doi:10.3390/s25134141)
Supplement: Supplementary file 1 [file sensors-25-04141-s001.zip › Asira.zip!/Asira/OCTAVE packages/parallel-4.0.2.tar.gz!/parallel-4.0.2/doc/html/precv.html]

precv (parallel\_doc)


Next: reval, Previous: psend, Up: Cluster execution   [Index]

---


### 3.13 Receiving Octave variables


Loadable Function: **precv** *(single\_connection)*
:   Receive a data value from the parallel cluster machine specified by single\_connection.

    This function can be called both at the client machine and (with
    `reval`) at a server machine. single\_connection must be a
    single connection obtained by indexing the connections
    variable. Please see `pconnect` for a description of the
    connections variable, and `pserver` for a description of
    this variable (named `sockets`) at the server side. If
    single\_connection corresponds to the machine at which
    `precv` was called, an error is thrown.

    The value received with `precv` must be sent with `psend`
    from another machine of the cluster. Note that data can be transferred
    this way between each pair of machines, even sent by a server and
    received by a different server.

    If `precv` is called at the client machine, a corresponding
    `psend` should have been called before at the source machine,
    otherwise the client will hang.

    **See also:** pconnect, pserver, reval, psend, sclose, parallel\_generate\_srp\_data, select\_sockets.
