# Supplementary material for: Development of a Thermal Infrared Network for Volcanic and Environmental Monitoring: Hardware Design and Data Analysis Software Code
Source: Sensors (Basel). 2025 Jul 2;25(13):4141. doi: 10.3390/s25134141 (PMC12252109; doi:10.3390/s25134141)
Supplement: Supplementary file 1 [file sensors-25-04141-s001.zip › Asira.zip!/Asira/OCTAVE packages/parallel-4.0.2.tar.gz!/parallel-4.0.2/doc/html/select.html]

select (parallel\_doc)


Previous: fsave fload, Up: Further functions   [Index]

---


### 4.2 Call Unix `select` on Octave streams

Note that for cluster connections select\_sockets should be used
instead.

Loadable Function: *[n, ridx, widx, eidx] =* **select** *(read\_fids, write\_fids, except\_fids, timeout[, nfds])*
:   Calls Unix `select`, see the respective manual.

    The following interface was chosen:
    read\_fids, write\_fids, except\_fids: vectors of stream-ids.
    timeout: seconds, negative for infinite.
    nfds: optional, default is Unix’ FD\_SETSIZE (platform specific).
    An error is returned if nfds or a filedescriptor belonging to a stream-id
    plus one exceeds FD\_SETSIZE.
    Return values are:
    n: number of ready streams.
    ridx, widx, eidx: index vectors of ready streams in
    read\_fids, write\_fids, and except\_fids, respectively.
