# Supplementary material for: Development of a Thermal Infrared Network for Volcanic and Environmental Monitoring: Hardware Design and Data Analysis Software Code
Source: Sensors (Basel). 2025 Jul 2;25(13):4141. doi: 10.3390/s25134141 (PMC12252109; doi:10.3390/s25134141)
Supplement: Supplementary file 1 [file sensors-25-04141-s001.zip › Asira.zip!/Asira/OCTAVE packages/parallel-4.0.2.tar.gz!/parallel-4.0.2/doc/html/pconnect.html]

pconnect (parallel\_doc)


Next: sclose, Previous: pserver, Up: Cluster execution   [Index]

---


### 3.4 Establishing cluster connections


Loadable Function: *connections =* **pconnect** *(hosts)*

Loadable Function: *connections =* **pconnect** *(hosts, options)*
:   Connects to a network of parallel cluster servers.

    As a precondition, a server must have been started at each machine of
    the cluster, see `pserver`. Connections are not guaranteed to
    work if client and server are from `parallel` packages of
    different versions, so versions should be kept equal.

    hosts is a cell-array of strings, holding the names of all
    server machines. The machines must be unique, and their names must be
    resolvable to the correct addresses also at each server machine, not
    only at the client. This means e.g. that the name `localhost` is
    not acceptable (exception: `localhost` is acceptable as the first
    of all names).

    Alternatively, but deprecated, hosts can be given as previously,
    as a character array with a machine name in each row. If it is given
    in this way, the first row must contain the name of the client machine
    (for backwards compatibility), so that there is one row more than the
    now preferred cell-array hosts would have entries.

    `pconnect` returns an opaque variable holding the network
    connections. This variable can be indexed to obtain a subset of
    connections or even a single connection. (For backwards compatibility,
    a second index of `:` is allowed, which has no effect). At the
    first index position is the client machine, so this position does not
    correspond to a real connection. At the following index positions are
    the server machines in the same order as specified in the cell-array
    hosts. So in the whole the variable of network connections has
    one position more than the number of servers given in hosts
    (except if hosts was given in the above mentioned deprecated
    way). You can display the variable of network connections to see what
    is in it. The variable of network connections, or subsets of it, is
    passed to the other functions for parallel cluster execution
    (`reval`, `psend`, `precv`, `sclose`,
    `select_sockets` among others – see documentation of these
    functions).

    options: structure of options; field `use_tls` is
    `true` by default (TLS with SRP authentication); if set to
    `false`, there will be no encryption or authentication. Field
    `password_file` can be set to an alternative path to the file
    with authentication information (see below). Field `user` can
    specify the username for authentication; if the username is so
    specified, no file with authentication information will be used at the
    client, but the password will be queried from the user.

    The client and the server must both use or both not use TLS. If TLS is
    switched off, different measures must be taken to protect ports 12501
    and 12502 at the servers and the client against unauthorized access;
    e.g. by a firewall or by physical isolation of the network.

    For using TLS, authorization data must be present at the server
    machine. These data can conveniently be generated by
    `parallel_generate_srp_data`. By default, the client
    authentication file is created in the same run. The helptext of
    `parallel_generate_srp_data` documents the expected locations of
    the authentication data.

    The SRP password will be sent over the encrypted TLS channel from the
    client to each server, to avoid permanently storing passwords at the
    server for server-to-server data connections. Due to inevitable usage
    of external libraries, memory with sensitive data can, however, be on
    the swap device even after shutdown of the application, both at the
    client and at the server machines.

    Example (let data travel through all machines), assuming
    `pserver` was called on each remote machine and authentication
    data is present (e.g. generated with
    `parallel_generate_srp_data`):

    ```
    sockets = pconnect ({'remote.machine.1', 'remote.machine.2'});
    reval ('psend (precv (sockets(2)), sockets(1))', sockets(3));
    reval ('psend (precv (sockets(1)), sockets(3))', sockets(2));
    psend ('some data', sockets(2));
    precv (sockets(3))
    --> ans = some data
    sclose (sockets);
    ```

    **See also:** pserver, reval, psend, precv, sclose, parallel\_generate\_srp\_data, select\_sockets, rfeval.

---

Next: sclose, Previous: pserver, Up: Cluster execution   [Index]
