# Supplementary material for: Development of a Thermal Infrared Network for Volcanic and Environmental Monitoring: Hardware Design and Data Analysis Software Code
Source: Sensors (Basel). 2025 Jul 2;25(13):4141. doi: 10.3390/s25134141 (PMC12252109; doi:10.3390/s25134141)
Supplement: Supplementary file 1 [file sensors-25-04141-s001.zip › Asira.zip!/Asira/OCTAVE packages/parallel-4.0.2.tar.gz!/parallel-4.0.2/doc/html/Example.html]

Example (parallel\_doc)


Previous: select\_sockets, Up: Cluster execution   [Index]

---


### 3.16 Example

```
# From Octave prompt, generate authentication files, set user name to
# 'test'. When prompted for a password, press <enter>.
parallel_generate_srp_data ('test')

# From Octave prompt, get location of the generated files.
authpath = fullfile (a = pkg ("prefix"), "parallel-srp-data")

Copy server files to servers, authpath is assumed to be
"/home/test/octave/parallel-srp-data/", the same directory is assumed to
exist on the servers. From the system shell, do e.g.:

scp -r /home/test/octave/parallel-srp-data/server server1:/home/test/octave/parallel-srp-data/
scp -r /home/test/octave/parallel-srp-data/server server2:/home/test/octave/parallel-srp-data/

Start server at remote machines. From the system shell, do e.g.:

ssh server1 'octave --eval "pserver"'
ssh server2 'octave --eval "pserver"'

# From Octave prompt, connect the cluster.
conns = pconnect ({"server1", "server2"})

# And perform some parallel execution. Single function calls take 1
# second each.
results = netcellfun (conns,  (x) {x, pause(1)}{:}, num2cell (1:30))

# Close network.
sclose (conns)
```
