# Supplementary material for: Development of a Thermal Infrared Network for Volcanic and Environmental Monitoring: Hardware Design and Data Analysis Software Code
Source: Sensors (Basel). 2025 Jul 2;25(13):4141. doi: 10.3390/s25134141 (PMC12252109; doi:10.3390/s25134141)
Supplement: Supplementary file 1 [file sensors-25-04141-s001.zip › Asira.zip!/Asira/OCTAVE packages/parallel-4.0.2.tar.gz!/parallel-4.0.2/doc/html/Limitations.html]

Limitations (parallel\_doc)


Next: Documentation, Previous: Further functions, Up: Top   [Index]

---


## 5 Limitations of high- and medium-level functions

Note that netcellfun, netarrayfun, and rfeval, but
even parcellfun and pararrayfun, execute code in separate
Octave processes. This causes some limitations.

For the function handles passed as arguments to parcellfun,
pararrayfun, netcellfun, netarrayfun, and
rfeval, the following limitations may apply:

- In Octave versions before 5.1, anonymous functions may only be
  passed if they don’t use `varargin` and don’t contain nested
  anonymous functions.
- If a handle to a named function, written in a function file,
  is passed, this function must exist on the server machine. This is
  usually no problem for parcellfun and pararrayfun.
- Named functions defined in the command line interface or by
  scripts are not exported to the working sessions used by
  parcellfun or pararrayfun, and not automatically to the
  server for remote parallel execution.
- For handles to subfunctions or to private functions limitations
  depending on the used Octave version may apply.

Anonymous functions should be usable as usual, since the function
specification sent to the server will include the anonymous functions
context.

If parcellfun or pararrayfun (and of course the
cluster-related functions, too) are called from within a function with
subfunctions, the subfunctions won’t be visible in parallel execution.

There is no way to set global variables for the function called by
`parcellun` or `pararrayfun`. For cluster execution, setting
global variables is possible, e.g. with install\_vars; however,
`netcellfun` and `netarrayfun` won’t ’see’ global variables if
they use `parcellfun` internally (which is the default at remote
machines with more than one available processor core).

---

Next: Documentation, Previous: Further functions, Up: Top   [Index]
