# Supplementary material for: Development of a Thermal Infrared Network for Volcanic and Environmental Monitoring: Hardware Design and Data Analysis Software Code
Source: Sensors (Basel). 2025 Jul 2;25(13):4141. doi: 10.3390/s25134141 (PMC12252109; doi:10.3390/s25134141)
Supplement: Supplementary file 1 [file sensors-25-04141-s001.zip › Asira.zip!/Asira/OCTAVE packages/parallel-4.0.2.tar.gz!/parallel-4.0.2/doc/html/Installation.html]

Installation (parallel\_doc)


Next: Local execution, Previous: Top, Up: Top   [Index]

---


## 1 Installation hints

*Note:* For using a cluster of machines, identical versions of the
package are required to be installed at each machine.

Maybe your distribution provides the parallel package.

If not, or if you want a newer version, Octaves `pkg` command
allows building the package from source and installing it. Note that you
have to `load` any Octave package before you can use it. See
Octaves documentation of `pkg`.

Parallel execution over a cluster by default is protected by encryption
with authentication. This needs a version of the gnutls library in which
TLS-SRP is not disabled. For building the parallel package from source,
this library must be present during the build; some operating systems
provide development files of libraries in exta `...-dev` packages,
which are also needed during the build.

Building or using the package for cluster execution without gnutls will
work, but encryption and authentication are not available in this case.
