# Supplementary material for: Development of a Thermal Infrared Network for Volcanic and Environmental Monitoring: Hardware Design and Data Analysis Software Code
Source: Sensors (Basel). 2025 Jul 2;25(13):4141. doi: 10.3390/s25134141 (PMC12252109; doi:10.3390/s25134141)
Supplement: Supplementary file 1 [file sensors-25-04141-s001.zip › Asira.zip!/Asira/OCTAVE packages/parallel-4.0.2.tar.gz!/parallel-4.0.2/doc/html/parcellfun.html]

parcellfun (parallel\_doc)


Next: pararrayfun, Up: Local execution   [Index]

---


### 2.1 Function parcellfun


Function File: *[o1, o2, …] =* **parcellfun** *(nproc, fun, a1, a2, …)*

Function File: **parcellfun** *(nproc, fun, …, "UniformOutput", val)*

Function File: **parcellfun** *(nproc, fun, …, "ErrorHandler", errfunc)*

Function File: **parcellfun** *(nproc, fun, …, "VerboseLevel", val)*

Function File: **parcellfun** *(nproc, fun, …, "ChunksPerProc", val)*

Function File: **parcellfun** *(nproc, fun, …, "CumFunc", cumfunc)*
:   Evaluates a function for multiple argument sets using multiple
    processes in parallel.

    nproc specifies the number of processes and must be at least
    1. It will be cut to the number of available CPU cores or the size
    of the input argument(s), whichever is lower. fun is a
    function handle pointing to the requested evaluating
    function. a1, a2 etc. should be cell arrays of equal
    size. o1, o2 etc. will be set to corresponding output
    arguments.

    The UniformOutput and ErrorHandler options are supported with
    meaning identical to *cellfun*. If option VerboseLevel is set
    to 1 (default is 0), progress messages are printed to stderr. The
    ChunksPerProc option controls the number of chunks which contains
    elementary jobs. This option is particularly useful when time
    execution of function is small. Setting this option to 100 is a
    good choice in most cases.

    Instead of returning a result for each argument, parcellfun returns
    only one cumulative result if "CumFunc" is non-empty. cumfunc
    must be a function which performs an operation on two sets of
    outputs of fun and returnes as many outputs as fun. If
    nout is the number of outputs of fun, cumfunc
    gets a previous output set of fun or of cumfunc as
    first nout arguments and the current output of fun as
    last nout arguments. The performed operation must be
    mathematically commutative and associative. If the operation is
    e.g. addition, the result will be the sum(s) of the outputs of
    fun over all calls of fun. Since floating point
    addition and multiplication are only approximately associative, the
    cumulative result will not be exactly reproducible.

    Each process has its own pseudo random number generator, so when
    using this function for example to perform Monte-Carlo simulations
    one generally cannot expect results to be exactly
    reproducible. Exact reproducibility may be achievable by generating
    the random number sequence before calling parcellfun and providing
    the random numbers as arguments to fun.

    See `parallel_doc ("limitations")` for possible limitations on
    how fun can be defined.

    **See also:** parcellfun\_set\_nproc.

---

Next: pararrayfun, Up: Local execution   [Index]
