# Supplementary material for: Development of a Thermal Infrared Network for Volcanic and Environmental Monitoring: Hardware Design and Data Analysis Software Code
Source: Sensors (Basel). 2025 Jul 2;25(13):4141. doi: 10.3390/s25134141 (PMC12252109; doi:10.3390/s25134141)
Supplement: Supplementary file 1 [file sensors-25-04141-s001.zip › Asira.zip!/Asira/OCTAVE packages/parallel-4.0.2.tar.gz!/parallel-4.0.2/doc/html/Authentication.html]

Authentication (parallel\_doc)


Next: pserver, Previous: Security, Up: Cluster execution   [Index]

---


### 3.2 Generating authentication keys


Loadable Function: **parallel\_generate\_srp\_data** *(username)*

Loadable Function: **parallel\_generate\_srp\_data** *(username, options)*
:   Prompts for a password (press enter for a random password) and writes TLS-SRP authentication files into the directory given by:

    `fullfile (a = pkg ("prefix"), "parallel-srp-data")`

    Server files are placed in subdirectory `server`. By default, a
    client authentication file is placed in subdirectory
    `client`. The latter contains the given username and the
    cleartext password. You do not need this file if you prefer to be
    prompted for username and password at connection time. In this case,
    you can prevent the client authentication file from being written by
    passing as the argument options a structure with a value of
    `false` in the field `unattended`.

    For authentication, subdir `server`, and possibly subdir
    `client`, have to be placed together with their contents at the
    respective machines (but see "Notes" below). They can either be
    placed under the directory given by:

    `fullfile (OCTAVE_HOME (), "share", "octave", "parallel-srp-data")`

    or – which might be the same directory – under:

    `fullfile (a = pkg ("prefix"), "parallel-srp-data")`

    Files in the former directory will take precedence over those in the
    latter. The contents of the files `passwd` and `user_passwd`
    (if present) must be kept secret.

    Notes: One of the two server files is named "passwd". It contains one
    line per user – the line starts with the username and a ":". If this
    file pre-exists, the new line will be appended to it. You may have to
    manually merge this file with a potentially pre-existing file at the
    server machine, e.g. if you want to add a new user there. Also, you
    have to manually assure that in the version of this file at the server
    machine each username appears only once (i.e. old lines for the same
    user may have to be deleted). The username is arbitrary.

    This function zeroizes sensitive data before releasing its memory. Due
    to usage of external libraries, however, it still can’t be excluded
    that sensitive data is still on the swap device after application
    shutdown.

    **See also:** pconnect, pserver, reval, psend, precv, sclose, select\_sockets.

---

Next: pserver, Previous: Security, Up: Cluster execution   [Index]
