# Supplementary material for: Development of a Thermal Infrared Network for Volcanic and Environmental Monitoring: Hardware Design and Data Analysis Software Code
Source: Sensors (Basel). 2025 Jul 2;25(13):4141. doi: 10.3390/s25134141 (PMC12252109; doi:10.3390/s25134141)
Supplement: Supplementary file 1 [file sensors-25-04141-s001.zip › Asira.zip!/Asira/OCTAVE packages/parallel-4.0.2.tar.gz!/parallel-4.0.2/doc/html/Concept-index.html]

Concept index (parallel\_doc)


Previous: Function index, Up: Top   [Index]

---


## Concept index

|  |  |
| --- | --- |
| Jump to: | **A**   **C**   **D**   **E**   **I**   **L**   **S** |

|  |  |  |  |
| --- | --- | --- | --- |
|  | Index Entry |  | Section |
| --- | | | |
| A |  |  |  |
|  | authentication: |  | Authentication |
| --- | | | |
| C |  |  |  |
|  | cluster execution: |  | Cluster execution |
| --- | | | |
| D |  |  |  |
|  | documentation: |  | Documentation |
| --- | | | |
| E |  |  |  |
|  | example: |  | Example |
| --- | | | |
| I |  |  |  |
|  | installation: |  | Installation |
| --- | | | |
| L |  |  |  |
|  | limitations: |  | Limitations |
|  | local execution: |  | Local execution |
| --- | | | |
| S |  |  |  |
|  | security: |  | Security |
| --- | | | |

|  |  |
| --- | --- |
| Jump to: | **A**   **C**   **D**   **E**   **I**   **L**   **S** |
