# Supplementary material for: Development of a Thermal Infrared Network for Volcanic and Environmental Monitoring: Hardware Design and Data Analysis Software Code
Source: Sensors (Basel). 2025 Jul 2;25(13):4141. doi: 10.3390/s25134141 (PMC12252109; doi:10.3390/s25134141)
Supplement: Supplementary file 1 [file sensors-25-04141-s001.zip › Asira.zip!/Asira/OCTAVE packages/parallel-4.0.2.tar.gz!/parallel-4.0.2/doc/html/network_005fget_005finfo.html]

network\_get\_info (parallel\_doc)


Next: network\_set, Previous: sclose, Up: Cluster execution   [Index]

---


### 3.6 Get network information


Loadable Function: **network\_get\_info** *(connections)*
:   Return an informational structure-array with one entry for each machine specified by connections.

    This function can only be successfully called at the client
    machine. See `pconnect` for a description of the
    connections variable. connections can contain all
    connections of the network, a subset of them, or a single
    connection. For the local machine (client), if contained in
    connections, some fields of the returned structure may be
    empty.

    The fields of the returned structure are `local_machine`: true
    for the connection representing the local machine, `nproc`:
    number of usable processors of the machine, `nlocaljobs`:
    configured number of local processes on the machine, `peername`:
    name of the machine (empty for local machine), `open`: true if
    the connection is open, `network_id`: uuid of the network,
    `real_node_id`: internal id assigned to node, `0` for
    client, servers starting with `1`.

    **See also:** pconnect, pserver, reval, psend, precv, sclose, parallel\_generate\_srp\_data, select\_sockets.
