# Supplementary material for: Development of a Thermal Infrared Network for Volcanic and Environmental Monitoring: Hardware Design and Data Analysis Software Code
Source: Sensors (Basel). 2025 Jul 2;25(13):4141. doi: 10.3390/s25134141 (PMC12252109; doi:10.3390/s25134141)
Supplement: Supplementary file 1 [file sensors-25-04141-s001.zip › Asira.zip!/Asira/OCTAVE packages/parallel-4.0.2.tar.gz!/parallel-4.0.2/doc/html/pararrayfun.html]

pararrayfun (parallel\_doc)


Next: parcellfun\_set\_nproc, Previous: parcellfun, Up: Local execution   [Index]

---


### 2.2 Function pararrayfun


Function File: *[o1, o2, …] =* **pararrayfun** *(nproc, fun, a1, a2, …)*

Function File: **pararrayfun** *(nproc, fun, …, "UniformOutput", val)*

Function File: **pararrayfun** *(nproc, fun, …, "ErrorHandler", errfunc)*
:   Evaluates a function for corresponding elements of an array.
    Argument and options handling is analogical to `parcellfun`, except that
    arguments are arrays rather than cells. If cells occur as arguments, they are treated
    as arrays of singleton cells.
    Arrayfun supports one extra option compared to parcellfun: "Vectorized".
    This option must be given together with "ChunksPerProc" and it indicates
    that fun is able to operate on vectors rather than just scalars, and returns
    a vector. The same must be true for errfunc, if given.
    In this case, the array is split into chunks which are then directly served to func
    for evaluation, and the results are concatenated to output arrays.
    If "CumFunc" is also specified (see `parcellfun`), fun is
    expected to return the result of the same cumulative operation
    instead of vectors.

See also parcellfun,
(octave)arrayfun.
