# Supplementary material for: Development of a Thermal Infrared Network for Volcanic and Environmental Monitoring: Hardware Design and Data Analysis Software Code
Source: Sensors (Basel). 2025 Jul 2;25(13):4141. doi: 10.3390/s25134141 (PMC12252109; doi:10.3390/s25134141)
Supplement: Supplementary file 1 [file sensors-25-04141-s001.zip › Asira.zip!/Asira/OCTAVE packages/parallel-4.0.2.tar.gz!/parallel-4.0.2/doc/html/fsave-fload.html]

fsave fload (parallel\_doc)


Next: select, Up: Further functions   [Index]

---


### 4.1 Transfer variable within the local machine over an Octave stream


Loadable Function: **fsave** *(fid, var)*
:   Save a single variable to a binary stream, to be subsequently loaded with
    fload.

Loadable Function: *var =* **fload** *(fid)*
:   Loads a single variable of any type from a binary stream, where it was previously
    saved with fsave.
