# Supplementary material for: Development of a Thermal Infrared Network for Volcanic and Environmental Monitoring: Hardware Design and Data Analysis Software Code
Source: Sensors (Basel). 2025 Jul 2;25(13):4141. doi: 10.3390/s25134141 (PMC12252109; doi:10.3390/s25134141)
Supplement: Supplementary file 1 [file sensors-25-04141-s001.zip › Asira.zip!/Asira/OCTAVE packages/parallel-4.0.2.tar.gz!/parallel-4.0.2/doc/html/psend.html]

psend (parallel\_doc)


Next: precv, Previous: rfeval, Up: Cluster execution   [Index]

---


### 3.12 Sending Octave variables


Loadable Function: **psend** *(value, connections)*
:   Send the value in variable value to all parallel cluster machines specified by connections.

    This function can be called both at the client machine and (with
    `reval`) at a server machine. See `pconnect` for a
    description of the connections variable, and `pserver` for
    a description of this variable (named `sockets`) at the server
    side. connections can contain all connections of the network, a
    subset of them, or a single connection. The machine at which
    `psend` was called (client or server), if contained in the
    connections variable, is ignored.

    The value sent with `psend` must be received with `precv` at
    the target machine. Note that values can be sent to each machine, even
    from a server machine to a different server machine.

    If `psend` is called at the client machine, a corresponding
    `precv` should have been called before at the target machine,
    otherwise the client will hang if value contains large data
    (which can not be held by the operating systems socket buffers).

    **See also:** pconnect, pserver, reval, precv, sclose, parallel\_generate\_srp\_data, select\_sockets.
