# Supplementary material for: Development of a Thermal Infrared Network for Volcanic and Environmental Monitoring: Hardware Design and Data Analysis Software Code
Source: Sensors (Basel). 2025 Jul 2;25(13):4141. doi: 10.3390/s25134141 (PMC12252109; doi:10.3390/s25134141)
Supplement: Supplementary file 1 [file sensors-25-04141-s001.zip › Asira.zip!/Asira/OCTAVE packages/parallel-4.0.2.tar.gz!/parallel-4.0.2/doc/html/Documentation.html]

Documentation (parallel\_doc)


Next: Function index, Previous: Limitations, Up: Top   [Index]

---


## 6 Function parallel\_doc to view documentation


Function File: **parallel\_doc** *()*

Function File: **parallel\_doc** *(keyword)*
:   Show parallel package documentation.

    Runs the info viewer Octave is configured with on the documentation
    in info format of the installed parallel package. Without argument,
    the top node of the documentation is displayed. With an argument,
    the respective index entry is searched for and its node displayed.
