# Supplementary material for: Development of a Thermal Infrared Network for Volcanic and Environmental Monitoring: Hardware Design and Data Analysis Software Code
Source: Sensors (Basel). 2025 Jul 2;25(13):4141. doi: 10.3390/s25134141 (PMC12252109; doi:10.3390/s25134141)
Supplement: Supplementary file 1 [file sensors-25-04141-s001.zip › Asira.zip!/Asira/OCTAVE packages/parallel-4.0.2.tar.gz!/parallel-4.0.2/doc/html/select_005fsockets.html]

select\_sockets (parallel\_doc)


Next: Example, Previous: reval, Up: Cluster execution   [Index]

---


### 3.15 Call Unix `select` for data connections


Loadable Function: **select\_sockets** *(connections, timeout)*

Loadable Function: **select\_sockets** *(connections, timeout, nfds)*
:   Calls Unix `select` for data connections in a parallel cluster.

    This function is for advanced usage (and therefore has minimal
    documentation), typically the programming of schedulers. It can be
    called at the client or at a server.

    connections: valid connections object (see `pconnect` and
    `pserver`, possibly indexed).

    timeout: seconds, negative for infinite.

    nfds: Passed to Unix `select` as first argument, see
    documentation of Uix `select`. Default: `FD_SETSIZE`
    (platform specific).

    An error is returned if nfds or a watched filedescriptor plus one
    exceeds FD\_SETSIZE.

    Returns an index vector to connections indicating connections
    with pending input, readable with `precv`.

    If called at the client, the command connections are included into the
    UNIX `select` call and checked for error flags, and
    `select_sockets` returns an error if a flag for a remote error is
    received.

    **See also:** pconnect, pserver, reval, psend, precv, sclose, parallel\_generate\_srp\_data.
