# Supplementary material for: Development of a Thermal Infrared Network for Volcanic and Environmental Monitoring: Hardware Design and Data Analysis Software Code
Source: Sensors (Basel). 2025 Jul 2;25(13):4141. doi: 10.3390/s25134141 (PMC12252109; doi:10.3390/s25134141)
Supplement: Supplementary file 1 [file sensors-25-04141-s001.zip › Asira.zip!/Asira/OCTAVE packages/parallel-4.0.2.tar.gz!/parallel-4.0.2/doc/html/install_005fvars.html]

install\_vars (parallel\_doc)


Next: rfeval, Previous: netarrayfun, Up: Cluster execution   [Index]

---


### 3.10 Distribute Octave variables


Function File: **install\_vars** *(varname, …, connections)*
:   Install named variables at remote machines.

    The variables named in the arguments are distrubted to the remote
    machines specified by connections and installed there. The
    variables must be accessible within the calling function. If
    variables have been declared to have global scope, they will also
    have global scope at the remote machines.

    This function can only be successfully called at the client machine.
    See `pconnect` for a description of the connections
    variable. connections can contain all connections of the
    network, a subset of them, or a single connection. The local machine
    (client), if contained in connections, is ignored.

    To install, e.g., all visible variables,

    `install_vars (who (){:}, …);`

    can be used.

    Internally, this function sends the variables only to one server and
    then issues the necessary commands to distribute them to all
    specified servers over server-to-server data connections.

    **See also:** pconnect, pserver, sclose, rfeval, netcellfun.
