# Supplementary material for: Development of a Thermal Infrared Network for Volcanic and Environmental Monitoring: Hardware Design and Data Analysis Software Code
Source: Sensors (Basel). 2025 Jul 2;25(13):4141. doi: 10.3390/s25134141 (PMC12252109; doi:10.3390/s25134141)
Supplement: Supplementary file 1 [file sensors-25-04141-s001.zip › Asira.zip!/Asira/OCTAVE packages/parallel-4.0.2.tar.gz!/parallel-4.0.2/doc/html/Local-execution.html]

Local execution (parallel\_doc)


Next: Cluster execution, Previous: Installation, Up: Top   [Index]

---


## 2 Functions for local parallel execution

Explicit local parallel execution, with the intent to exploit more than
one local processor(-core), is performed by calling a user-defined
function in parallel with several different arguments. This is done in
parallel *processes*. Note that there is no way to pass global
variables to or from these processes.

The interface of the functions for local parallel execution is similar
to Octaves `cellfun` and `arrayfun` functions.

Note that some operations in Octave, particularly some matrix
operations, may already be performed in parallel threads. This may limit
the advantage yielded by explicit local parallel execution. Also, RAM
access can be a bottleneck which limits computation speed of multicore
computing.

For limitations, see Limitations.

|  |  |  |
| --- | --- | --- |
| • parcellfun |  | Function parcellfun. |
| • pararrayfun |  | Function pararrayfun. |
| • parcellfun\_set\_nproc |  | Function parcellfun\_set\_nproc:: |
