# Supplementary material for: Development of a Thermal Infrared Network for Volcanic and Environmental Monitoring: Hardware Design and Data Analysis Software Code
Source: Sensors (Basel). 2025 Jul 2;25(13):4141. doi: 10.3390/s25134141 (PMC12252109; doi:10.3390/s25134141)
Supplement: Supplementary file 1 [file sensors-25-04141-s001.zip › Asira.zip!/Asira/OCTAVE packages/parallel-4.0.2.tar.gz!/parallel-4.0.2/doc/html/Further-functions.html]

Further functions (parallel\_doc)


Next: Limitations, Previous: Cluster execution, Up: Top   [Index]

---


## 4 Functions possibly helpful in parallel execution

|  |  |  |
| --- | --- | --- |
| • fsave fload |  | Transfer variable within the local machine over an Octave stream. |
| • select |  | Call Unix `select` on Octave streams. |
