# Supplementary material for: Development of a Thermal Infrared Network for Volcanic and Environmental Monitoring: Hardware Design and Data Analysis Software Code
Source: Sensors (Basel). 2025 Jul 2;25(13):4141. doi: 10.3390/s25134141 (PMC12252109; doi:10.3390/s25134141)
Supplement: Supplementary file 1 [file sensors-25-04141-s001.zip › Asira.zip!/Asira/OCTAVE packages/parallel-4.0.2.tar.gz!/parallel-4.0.2/doc/html/reval.html]

reval (parallel\_doc)


Next: select\_sockets, Previous: precv, Up: Cluster execution   [Index]

---


### 3.14 Remote command evaluation


Loadable Function: **reval** *(commands, connections)*
:   Evaluate commands at all remote hosts specified by connections.

    This function can only be successfully called at the client
    machine. commands must be a string containing Octave commands
    suitable for execution with Octaves `eval()` function. See
    `pconnect` for a description of the connections
    variable. connections can contain all connections of the
    network, a subset of them, or a single connection. The local machine
    (client), if contained in connections, is
    ignored. commands is executed in the same way at each specified
    machine.

    **See also:** pconnect, pserver, psend, precv, sclose, parallel\_generate\_srp\_data, select\_sockets.
