# Supplementary material for: Development of a Thermal Infrared Network for Volcanic and Environmental Monitoring: Hardware Design and Data Analysis Software Code
Source: Sensors (Basel). 2025 Jul 2;25(13):4141. doi: 10.3390/s25134141 (PMC12252109; doi:10.3390/s25134141)
Supplement: Supplementary file 1 [file sensors-25-04141-s001.zip › Asira.zip!/Asira/OCTAVE packages/parallel-4.0.2.tar.gz!/parallel-4.0.2/doc/html/rfeval.html]

rfeval (parallel\_doc)


Next: psend, Previous: install\_vars, Up: Cluster execution   [Index]

---


### 3.11 Single remote function evaluation


Function File: **rfeval** *(func, …, nout, isout, connection)*
:   Evaluate a function at a remote machine.

    func is evaluated with arguments `…` and number of
    output arguments set to nout at remote machine given by
    connection. If isout is not empty, it must be a logical
    array with nout elements, which are true for each of the
    nout output arguments which are requested from the function;
    the other output arguments will be marked as not requested
    with `~` at remote execution.

    This function can only be successfully called at the client machine.
    See `pconnect` for a description of the connection
    variable. connection must contain one single connection.

    If an output argument is given to `rfeval`, the function waits
    for completion of the remote function call, retrieves the results and
    returns them. They will be returned as one cell-array with an entry
    for each output argument. If some output arguments are marked as not
    requested by setting some elements of isout to false, the
    returned cell-array will only have entries for the requested output
    arguments. For consistency, the returned cell-array can be empty. To
    assign the output arguments to single variables, you can for example
    use: `[a, b, c] = returned_cell_array{:};`.

    If no output argument is given to `rfeval`, the function does
    not retrieve the results of the remote function call but returns
    immediately. It is left to the user to retrieve the results with
    `precv`. The results will be in the same format as if returned
    by `rfeval`. Note that a cell-array, possibly empty, will always
    have to be retrieved, even if the remote function call should have
    been performed without output arguments.

    Parallel execution can be achieved by calling `rfeval` several
    times with different specified server machines before starting to
    retrieve the results.

    The specified function handle can refer to a function present at
    the executing machine or be an anonymous function. See
    `parallel_doc ("limitations")` for possible limitations.

    **See also:** pconnect, pserver, sclose, install\_vars, netcellfun.

---

Next: psend, Previous: install\_vars, Up: Cluster execution   [Index]
