# Supplementary material for: Development of a Thermal Infrared Network for Volcanic and Environmental Monitoring: Hardware Design and Data Analysis Software Code
Source: Sensors (Basel). 2025 Jul 2;25(13):4141. doi: 10.3390/s25134141 (PMC12252109; doi:10.3390/s25134141)
Supplement: Supplementary file 1 [file sensors-25-04141-s001.zip › Asira.zip!/Asira/OCTAVE packages/parallel-4.0.2.tar.gz!/parallel-4.0.2/doc/html/Security.html]

Security (parallel\_doc)


Next: Authentication, Up: Cluster execution   [Index]

---


### 3.1 Security considerations

Over a connection to a server of the parallel package arbitrary Octave
commands can be executed at the server machine. This means that any
change can be done to the server system and the resident data, only
limited by the rights of the system account under which the server runs.

By default, the server is started with authentication and encryption
enabled, to avoid unauthorized access. If the server is started with
authentication disabled (maybe to avoid the encryption overhead), it
must be cared for that no TCP connection by unauthorized persons is
possible to the server ports, possibly by running the client and all
server machines behind a firewall and assuring that only trusted persons
have access to any machine behind the firewall. This scenario might be
achievable in home-nets.

The server currently uses port 12502 for receiving commands and port
12501 for data exchange.

The client and the servers used by the client with `pconnect` must
agree on using authentication or not.

Do not start the server as root.
