# Supplementary material for: Development of a Thermal Infrared Network for Volcanic and Environmental Monitoring: Hardware Design and Data Analysis Software Code
Source: Sensors (Basel). 2025 Jul 2;25(13):4141. doi: 10.3390/s25134141 (PMC12252109; doi:10.3390/s25134141)
Supplement: Supplementary file 1 [file sensors-25-04141-s001.zip › Asira.zip!/Asira/OCTAVE packages/parallel-4.0.2.tar.gz!/parallel-4.0.2/doc/html/netarrayfun.html]

netarrayfun (parallel\_doc)


Next: install\_vars, Previous: netcellfun, Up: Cluster execution   [Index]

---


### 3.9 Function netarrayfun


Function File: **netarrayfun** *(connections, fun, …)*
:   Evaluates function fun in a parallel cluster and collects results.

    This function handles arguments and options equivalently to
    `pararrayfun` and returnes equivalent output. Differently, the
    first argument specifies server machines for parallel remote
    execution, see `pconnect` for a description of the
    connections variable. A further difference is that the option
    "ChunksPerProc" is ignored and instead the chunk size can be
    specified directly with an option "ChunkSize" (option "Vectorized"
    can be used together with option "ChunkSize" in function
    `netarrayfun`).

    The further details of operation are the same as for
    `netcellfun`, please see there.

    **See also:** netcellfun, pconnect, pserver, sclose, rfeval, install\_vars.
