# Supplementary material for: Development of a Thermal Infrared Network for Volcanic and Environmental Monitoring: Hardware Design and Data Analysis Software Code
Source: Sensors (Basel). 2025 Jul 2;25(13):4141. doi: 10.3390/s25134141 (PMC12252109; doi:10.3390/s25134141)
Supplement: Supplementary file 1 [file sensors-25-04141-s001.zip › Asira.zip!/Asira/OCTAVE packages/parallel-4.0.2.tar.gz!/parallel-4.0.2/doc/html/network_005fset.html]

network\_set (parallel\_doc)


Next: netcellfun, Previous: network\_get\_info, Up: Cluster execution   [Index]

---


### 3.7 Set network properties


Loadable Function: **network\_set** *(connections, key, val)*
:   Set the property named by key to the value val for each machine specified by connections.

    This function can only be successfully called at the client
    machine. See `pconnect` for a description of the
    connections variable. connections can contain all
    connections of the network, a subset of them, or a single
    connection.

    Possible values of key: `'nlocaljobs'`: configured number
    of local processes on the machine (usable by functions for parallel
    execution); needs a non-negative integer in val, `0` means
    not specified.

    **See also:** pconnect, pserver, reval, psend, precv, sclose, parallel\_generate\_srp\_data, select\_sockets.
