# Supplementary material for: Development of a Thermal Infrared Network for Volcanic and Environmental Monitoring: Hardware Design and Data Analysis Software Code
Source: Sensors (Basel). 2025 Jul 2;25(13):4141. doi: 10.3390/s25134141 (PMC12252109; doi:10.3390/s25134141)
Supplement: Supplementary file 1 [file sensors-25-04141-s001.zip › Asira.zip!/Asira/OCTAVE packages/parallel-4.0.2.tar.gz!/parallel-4.0.2/doc/html/sclose.html]

sclose (parallel\_doc)


Next: network\_get\_info, Previous: pconnect, Up: Cluster execution   [Index]

---


### 3.5 Closing cluster connections


Loadable Function: **sclose** *(connections)*
:   Close the parallel cluster network to which connections belongs.

    See `pconnect` for a description of the connections
    variable. All connections of the network are closed, even if
    connections contains only a subnet or a single connection.

    **See also:** pconnect, pserver, reval, psend, precv, parallel\_generate\_srp\_data, select\_sockets.
