# Supplementary material for: Development of a Thermal Infrared Network for Volcanic and Environmental Monitoring: Hardware Design and Data Analysis Software Code
Source: Sensors (Basel). 2025 Jul 2;25(13):4141. doi: 10.3390/s25134141 (PMC12252109; doi:10.3390/s25134141)
Supplement: Supplementary file 1 [file sensors-25-04141-s001.zip › Asira.zip!/Asira/OCTAVE packages/parallel-4.0.2.tar.gz!/parallel-4.0.2/doc/html/pserver.html]

pserver (parallel\_doc)


Next: pconnect, Previous: Authentication, Up: Cluster execution   [Index]

---


### 3.3 Starting servers


Function File: **pserver** *()*

Function File: **pserver** *(options)*

Function File: **pserver** *("kill")*
:   Starts or stops a server of the parallel cluster.

    options: structure of options; field `use_tls` is
    `true` by default (TLS with SRP authentication); if set to
    `false`, there will be no encryption or authentication. Field
    `auth_file` can be set to an alternative path to the file with
    authentication information (see below). Fields `cmd_port`
    (default: 12502) and `data_port` (default: 12501) can be set
    to change the ports of the command channel and the data channel,
    respectively.

    If called as `pserver ("kill")`, the server will be stopped by
    sending it a signal, taking its process id from its pid-file
    ’/tmp/.octave-<hostname>.pid’. Otherwise the server will be
    started.

    The servers exectuable file (`octave-pserver`) is searched for
    by first assuming the directory structure of a regular package
    installation, then by searching Octaves function search path for
    it, and then by the called shell in its shell search path.

    If a directory path corresponding to the current directory of the
    client exists on the server machine, it will be used as the servers
    current directory for the respective client (multiple clients are
    possible). Otherwise, `/tmp` will be used. The Octave
    functions the server is supposed to call and the files it possibly
    has to access must be available at the server machine. This can
    e.g. be achieved by having the server machine mount a network file
    system (which is outside the scope of this package documentation).

    If a connection is accepted from a client, the server collects a
    network identifier and the names of all server machines of the
    network from the client. Then, connections are automatically
    established between all machines of the network. Data exchange will
    be possible between all machines (client or server) in both
    directions. Commands can only be sent from the client to any
    server.

    The opaque variable holding the network connections, in the same
    order as in the corresponding variable returned by `pconnect`,
    is accessible under the variable name `sockets` at the server
    side. Do not overwrite or clear this variable. The own server
    machine will also be contained at some index position of this
    variable, but will not correspond to a real connection. See
    `pconnect` for further information.

    The client and the server must both use or both not use TLS. If TLS
    is switched off, different measures must be taken to protect ports
    of the command and data channels at the servers and the client
    against unauthorized access, e.g. by a firewall or by physical
    isolation of the network.

    For using TLS, authorization data must be present at the server
    machine. These data can conveniently be generated by
    `parallel_generate_srp_data`; the helptext of the latter
    function documents the expected location of these data.

    The SRP password will be sent over the encrypted TLS channel from
    the client to each server, to avoid permanently storing passwords
    at the server for server-to-server data connections. Due to
    inevitable usage of external libraries, memory with sensitive data
    can, however, be on the swap device even after shutdown of the
    application.

    The server executable file `octave-pserver` is installed and
    runs at GNU/Linux, but not at some other operating systems like
    Windows and macOS.

    **See also:** pconnect, reval, psend, precv, sclose, parallel\_generate\_srp\_data, select\_sockets.

---

Next: pconnect, Previous: Authentication, Up: Cluster execution   [Index]
