# Supplementary material for: Development of a Thermal Infrared Network for Volcanic and Environmental Monitoring: Hardware Design and Data Analysis Software Code
Source: Sensors (Basel). 2025 Jul 2;25(13):4141. doi: 10.3390/s25134141 (PMC12252109; doi:10.3390/s25134141)
Supplement: Supplementary file 1 [file sensors-25-04141-s001.zip › Asira.zip!/Asira/OCTAVE packages/parallel-4.0.2.tar.gz!/parallel-4.0.2/doc/html/index.html]

Top (parallel\_doc)


# parallel\_doc

Next: Installation   [Index]

---


# General documentation for the parallel package for Octave

The info version of this document is accessible, after package
installation, from the Octave commandline with `parallel_doc()`.

This documentation applies to version 4.0.2 of the parallel
package.

The package contains functions for explicit local parallel execution
and functions for parallel execution over a cluster of machines,
possibly combined with local parallel execution at each machine of the
cluster.

Earlier versions of the package used Octave sessions directly
generated by the `fork` system call. Since this causes problems,
the current version uses newly started Octave sessions, even for local
parallel execution. This causes some limitations, see
Limitations.

The server for cluster execution is installed at GNU/Linux, but not at
some other operating systems, e.g. not at Windows or macOS. The client
functions for cluster execution should be installed at all systems,
though.

|  |  |  |
| --- | --- | --- |
| • Installation |  | Installation hints. |
| • Local execution |  | Functions for local parallel execution. |
| • Cluster execution |  | Functions for parallel execution over a cluster of machines. |
| • Further functions |  | Functions possibly helpful in parallel execution. |
| • Limitations |  | Limitations of high- and medium-level functions. |
|  | | |
| • Documentation |  | Function parallel\_doc to view documentation. |
| ``` Indices ``` | | |
| • Function index |  | Index of functions in database. |
| • Concept index |  | Concept index. |

---

Next: Installation   [Index]
